# ISG15 and ISGylation is required for pancreatic cancer stem cell mitophagy and metabolic plasticity

**DOI:** 10.1038/s41467-020-16395-2

**Published:** 2020-05-29

**Authors:** Sonia Alcalá, Patricia Sancho, Paola Martinelli, Diego Navarro, Coral Pedrero, Laura Martín-Hijano, Sandra Valle, Julie Earl, Macarena Rodríguez-Serrano, Laura Ruiz-Cañas, Katerin Rojas, Alfredo Carrato, Laura García-Bermejo, Miguel Ángel Fernández-Moreno, Patrick C. Hermann, Bruno Sainz

**Affiliations:** 1 Department of Biochemistry, Universidad Autónoma de Madrid (UAM) and Instituto de Investigaciones Biomédicas “Alberto Sols” (IIBM), CSIC-UAM, Madrid, Spain; 2 Chronic Diseases and Cancer Area 3—Instituto Ramón y Cajal de Investigación Sanitaria (IRYCIS), Madrid, Spain; 3 IIS Aragón, Hospital Universitario Miguel Servet, Zaragoza, Spain; 4 Institute for Cancer Research, Comprehensive Cancer Center, Medical University Vienna, Vienna, Austria; 5 Medical Oncology Department, Ramón y Cajal University Hospital, Alcala University, Madrid, Spain; 6 Biomedical Research Network in Cancer (CIBERONC, CB16/12/00446), Madrid, Spain; 7 Biomarkers and Therapeutic Targets Group—IRYCIS, Madrid, Spain; 8 Centro de Investigación Biomédica en Red en Enfermedades Raras (CIBERER), Madrid, Spain; 9 Instituto de Investigación Sanitaria Hospital 12 de Octubre (imas12), Madrid, Spain; 10 Department of Internal Medicine I, Ulm University, Ulm, Germany

**Keywords:** Cancer metabolism, Cancer stem cells, Pancreatic cancer

## Abstract

Pancreatic cancer stem cells (PaCSCs) drive pancreatic cancer tumorigenesis, chemoresistance and metastasis. While eliminating this subpopulation of cells would theoretically result in tumor eradication, PaCSCs are extremely plastic and can successfully adapt to targeted therapies. In this study, we demonstrate that PaCSCs increase expression of interferon-stimulated gene 15 (ISG15) and protein ISGylation, which are essential for maintaining their metabolic plasticity. CRISPR-mediated ISG15 genomic editing reduces overall ISGylation, impairing PaCSCs self-renewal and their in vivo tumorigenic capacity. At the molecular level, ISG15 loss results in decreased mitochondrial ISGylation concomitant with increased accumulation of dysfunctional mitochondria, reduced oxidative phosphorylation (OXPHOS) and impaired mitophagy. Importantly, disruption in mitochondrial metabolism affects PaCSC metabolic plasticity, making them susceptible to prolonged inhibition with metformin in vivo. Thus, ISGylation is critical for optimal and efficient OXPHOS by ensuring the recycling of dysfunctional mitochondria, and when absent, a dysregulation in mitophagy occurs that negatively impacts PaCSC stemness.

## Abstract (web summary)

The ubiquitin-like modifier ISG15 exerts post-translational protein regulation through ISGylation. Here, the authors show that ISGylation is necessary for pancreatic cancer stem cell self-renewal and tumourigenesis by supporting the recycling of non-functional mitochondria.

## Introduction

Pancreatic ductal adenocarcinoma (PDAC) is currently the fourth most frequent cause of cancer-related deaths worldwide, and is projected to become the second deadliest cancer by 2030 (ref. ^1^). Clinically, since PDAC often goes undetected^2^, patients are typically diagnosed at late stages, when therapeutic intervention only slightly improves overall survival beyond 6 months and very rarely result in long-term (>5 years) progression-free survival^3^. Thus, in light of decades of research, effective therapies for this disease remain limited.

In order to achieve advancements, it is important to appreciate that pancreatic tumors are extremely heterogenous^4^. Among this heterogenous population of cells exists the so-called cancer stem cell (CSC), that not only drives tumor heterogeneity by giving rise to all of the other cancer cells (i.e., non-CSCs) present within the tumor, but is also responsible for post therapeutic disease relapse. Therefore, eliminating this subpopulation of stem-like tumor cells may represent the only successful strategy to treat PDAC. Unfortunately, the evolving concept that non-CSCs can convert into CSCs when the latter are directly eliminated highlights that CSC-specific-targeted therapies will likely not be effective on their own^5,6^. Likewise, CSCs themselves are extremely plastic and can efficiently adapt to intrinsic and extrinsic insults or stress^7^, reinforcing the need to better understand the factors that mediate their plasticity and the CSC state. An example of CSC plasticity, and one that may be therapeutically exploited, is metabolic plasticity^8^. We and others have shown that PaCSCs can meet their energy requirements via mitochondrial oxidative phosphorylation (OXPHOS) or, conversely, they can switch to glycolysis based on specific environmental conditions or insults^9,10^. Moreover, we identified two subpopulations of PaCSCs that coexist but with different metabolic phenotypes: one being OXPHOS-dependent with full stemness, and the other displaying a more plastic phenotype, at the expense of reduced self-renewal and tumorigenicity^10^. The latter was experimentally shown to be the subpopulation responsible for the development of in vivo resistance to metformin, an inhibitor of complex I of the mitochondrial electron transport chain (ETC)^11^. Our and other studies now confirm that these plastic cells are capable of overcoming the inhibitory effects of mitochondrial inhibitors, by switching to anaerobic glycolytic metabolism^9,10,12,13^. Thus, if CSC plasticity could be effectively targeted, therapies that modulate CSC metabolism would be more effective.

In general, cellular plasticity or cell fate transitions involve rapid changes in gene expression patterns and/or protein post-translational modifications (PTMs). Regarding the latter, PTMs have been shown to be involved in cell fate decisions; however, their relevance in CSC plasticity remains underexplored. One of the best studied PTM processes is ubiquitination, a covalent PTM that conjugates ubiquitin (Ub) to lysine residues on protein substrates, regulating their stability and function. Ubiquitination and de-ubiquitination are the major cellular processes used to balance the protein turnover of several transcription factors that regulate stem cell differentiation^14^, and the proper coordination of ubiquitylation and deubiquitylation is necessary for efficient stemness and differentiation^15^. Moreover, ubiquitination is a critical regulatory process in several metabolic processes such as mitophagy, the removal of damaged mitochondria via a selective form of autophagy. Apart from removing dysfunctional mitochondria, mitophagy is also necessary for proper cellular functions and cell fate determination^16,17^, and an increasing number of studies suggest that alterations in mitophagy can severely impact (stem) cell phenotypes and cellular plasticity^16,18^. Thus, PTMs can affect cellular plasticity or cell fate transitions at multiple levels.

In addition to Ub, Ub-like (UbL) modifiers also play an important role in PTMs and mitophagy. The best-known UbL modifier is SUMO, which has been implicated in a wide range of cellular processes, including cell identity and cancer progression^19^, as well as mitochondrial dynamics^20^. With more and more studies linking Ub and UbL modifiers to cell fate determination and metabolic plasticity, it is highly plausible that Ub- and UbL-mediated PTMs may also participate in CSC identity and plasticity. In the present study we determine the expression of UbL modifiers in PaCSCs and discover that the UbL modifier Interferon-stimulated gene 15 (ISG15), specifically its PTM process known as ISGylation, is upregulated in PaCSCs and is a necessary key process for PaCSC stemness, tumorigenesis and metabolic plasticity.

## Results

### ISG15 is enriched in PaCSCs and predicts patient survival

**Fig. 1:**
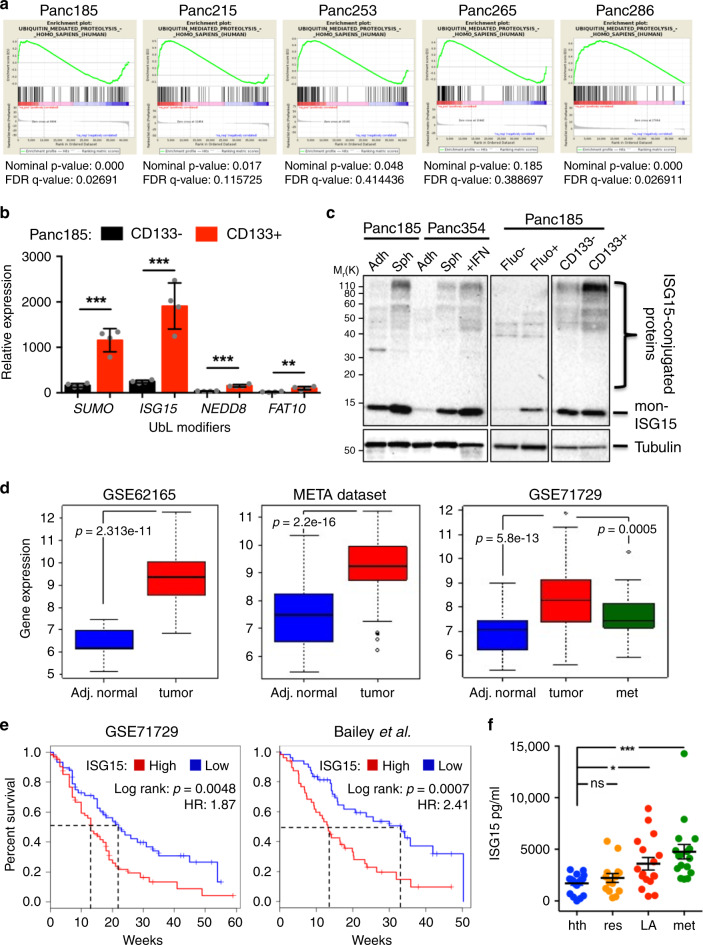
Ub and UbL pathways are enriched in PaCSCs and predict survival. **a** Ubiquitin pathway enrichment plots from RNAseq analysis (ArrayExpress: E-MTAB-3808) of sphere and adherent cultures (CSCs and non-CSCs, respectively) derived from five different primary PDX PDAC cultures. **b** Mean relative mRNA levels ± sd of UbL modifiers *ISG15*, *SUMO*, *NEDD8*, and *FAT10* in CD133 + and CD133– cells sorted from Panc185 spheres. Data are normalized to β-Actin mRNA expression. (*n* = 4 biologically independent sortings; **p = 0.0058; ***p < 0.001, as determined by Student’s *t*-test). **c** Western blot (WB) analysis of monomeric (mon)-ISG15 and ISG15-conjugated proteins in non-CSCs [adherent (adh), Fluo- and CD133–] versus CSCs [spheres (sph), Fluo+ or CD133 + ] from indicated PDX-derived cultures. Interferon (IFN)-treated Panc354 cells was used as positive control and tubulin as a loading control. Molecular weight markers kDa M_r_(K) are shown. **d** Box and Whisker Plots showing the differential expression of *ISG15* in normal adjacent (Adj.) tissue versus PDAC tumors and metastasis (met) in three independent transcriptomic data series: GSE62165 (13 Adj. normal, 118 tumors), META data set (70 Adj. normal, 108 tumors), GSE71729 (45 Adj. normal, 145 tumors, 61 mets). Rectangles show the first quartile, the median, and the third quartile. The two whiskers indicate the minimum and maximum values, and outliers are depicted as circles (unpaired two-sided Student’s *t*-test). **e** Kaplan–Meier curves showing the overall survival of PDAC patients in two independent data series: GSE71729 (*n* = 145) and Bailey (*n* = 96), stratified according to the median value of ISG15 expression. HR hazard ratio. A Log-rank test was performed for survival analysis and a Cox Proportional Hazard regression model to calculate HRs. **f** Mean ISG15 protein levels (pg/mL) ± sem present in serum form healthy (hth) (*n* = 21), resectable (res) (*n* = 14), locally advanced (LA) (*n* = 17), and metastasis (met) (*n* = 19) patients. *p < 0.05; ***p < 0.001; ns, not significant, as determined by one-way ANOVA with Bonferroni’s multiple comparisons test.

RNA sequencing (RNA-seq ArrayExpress: E-MTAB-3808) of PaCSCs previously showed upregulation of genes involved in OXPHOS, linking mitochondrial respiration to PaCSC stemness^10^. Further probing of the PaCSC transcriptome revealed enrichment of the Ub-mediated proteolysis pathway in PaCSCs (Fig. 1a). Since modulation of Ub and Ub-related enzymes in CSCs has been previously described and extensively studied^21^, we asked whether the less well-studied family of UbL modifiers, in the context of CSCs, were also differentially expressed. RTqPCR analysis revealed that *ISG15*, *SUMO*, *NEDD8*, and *FAT10* were significantly increased in CD133 + versus CD133– cells (traditional CSC marker), highlighting that increased transcription of UbL genes takes place in PaCSCs (Fig. 1b).

We recently published that ISG15 secreted by tumor-associated macrophages (TAMs) can activate PaCSCs via a paracrine mechanism of action^22^; however, the expression and role of endogenous ISG15 and ISGylation in PaCSCs has not been analyzed to date. Enrichment for PaCSCs using various approaches, including chemoresistance, growth as spheres or fluorescence-activated cell sorting (FACS) for the CSC markers CD133 or autofluorescence^23^, revealed increased *ISG15* messenger RNA (mRNA) levels, increased monomeric ISG15 (mon-ISG15) protein levels, and increased protein ISGylation in PaCSCs versus non-PaCSCs (Fig. 1c and Supplementary Fig. 1a–c), indicating a CSC-specific enrichment. ISG15 expression is regulated by Type I IFNα/β receptor (IFNAR)-mediated signaling and similar to ubiquitination, ISGylation is regulated by an E1-E2-E3 enzymatic cascade^24^. We have previously shown that Type I IFN signaling is activated in PaCSCs, and PaCSCs secrete functional IFN-β^22^. Accordingly, we observed that CSC-enriched sphere cultures expressed higher levels of the ISG15 transcriptional regulators pSTAT1 and IRF9 (Supplementary Fig. 1d), which are downstream of the IFNAR. Higher mRNA levels of the E1-activating enzyme Ube1L, E2-conjugating enzyme Ube2L6 and E3 ligase Herc5 were also observed (Supplementary Fig. 1e), indicating that the ISG15/ISGylation pathway is activated in PaCSCs.

Using the publicly available transcriptome data sets (GSE62165 (ref. ^25^), META data set^26^, and GSE71729 (ref. ^27^)), *ISG15* transcriptional levels were evaluated. Importantly, since ISG15 is also expressed by TAMs in the tumor microenvironment (TME)^22^, the META data set [consisting of four published PDAC gene expression studies (*n* = 108) including data sets with tumor cellularity >35% (GSE32688) or micro-dissected samples (GSE15471)] and the Moffitt series [which applies a virtual microdissection approach to identify distinct tumor- and stroma-specific signatures] allowed for the identification of genes preferentially overexpressed in tumor epithelia. For all data sets, *ISG15* mRNA levels were significantly elevated in tumor samples or metastases versus adjacent normal tissue (Fig. 1d and Supplementary Fig. 2a, b). In addition, tumors of the basal subtype, having a worse prognosis^28^, expressed significantly higher levels of *ISG15* compared to classical subtype tumors, but no significant difference in *ISG15* expression was observed across stromal subtypes, although a marked increase was appreciated in activated stroma (Supplementary Fig. 2c, d). For the GSE71729 (ref. ^27^) and Bailey^28^ series, well-annotated clinical data is available and was used to show in both data sets a clear deviation and significant decrease in median overall survival for *ISG15* high-expressing patients compared to *ISG15* low-expressing patients (Fig. 1e). Lastly, quantification of secreted ISG15 in serum revealed significantly increased levels in PDAC patients versus healthy controls, and a clear correlation with disease progression (Fig. 1f). Altogether, these results confirm the clinical relevance of ISG15 in PDAC.

### ISG15 expression is linked to mitochondria-related pathways

**Fig. 2:**
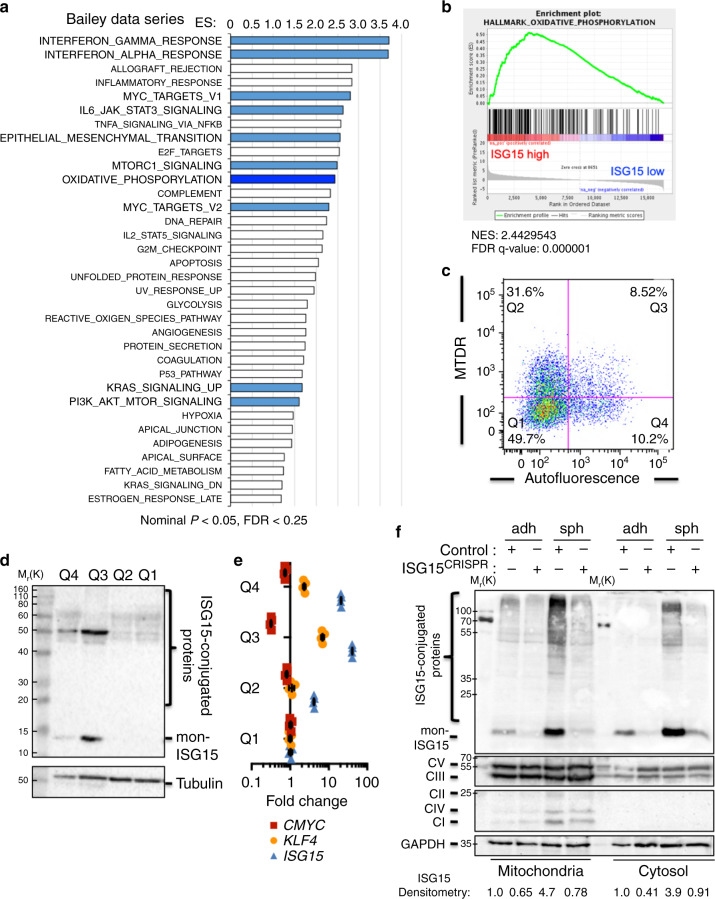
ISG15 expression is linked to mitochondria-related pathways. **a** Gene sets enriched in the transcriptional profile of tumors belonging to the top *ISG15* high-expression group, compared with the bottom expression group in the Bailey data series. Shown are the NES (normalized enrichment score) values for each pathway using the Hallmark genesets, meeting the significance criteria: nominal *p*-value of <0.05, FDR < 25%. **b** Enrichment plot for OXPHOS signaling in *ISG15* high versus low. **c** Autofluorescence (CSC) and Mitotracker Deep Red (MTDR, approximation of mitochondrial mass) were combined to sort the four gated populations (Q1–Q4) from Panc185 spheres. **d** WB analysis of ISG15 protein expression in the four FACS sorted populations in **c**. Tubulin was included as a loading control. **e** RTqPCR analysis of *CMYC*, *KLF4* and *ISG15* gene mean fold change ± sd in the four FACS sorted populations in **c** (*n* = 3 replicates from one independent sorting experiment). Values compared to Q1, set as 1.0. **f** WB analysis of ISG15 protein expression in mitochondrial and cytosolic fraction from Panc185 Control or CRISPR-Cas9 ISG15-edited (ISG15^CRISPR^) CSCs (sph) and non-CSCs (adh). The membrane was additionally blotted for mitochondria OXPHOS complex proteins using the Mitoprofile Total OXPHOS antibody cocktail in addition to GAPDH (loading control). Shown are bands corresponding to Complex (C)V, CIII, CII, CIV, and CI. Total ISG15 expression [ISG15-conjugated and monomeric (mon-) proteins] for each sample was quantified by densitometric analysis and fold changes are shown, relative to control adherent (adh), set as 1.0 for both mitochondria and cytosolic fractions.

Next, GSEA comparing the samples belonging to the top and bottom quartiles of ISG15 expression was performed using the Bailey and META data set series. Using the Hallmark genesets collection, we observed significantly and commonly enriched IFN and stem-associated pathways across both series, including TGF-β, mTOR, KRas, IL-6/JAK/STAT3, and PI3K/AKT/MTOR, as well as epithelial to mesenchymal transition (EMT) signaling (Fig. 2a and Supplementary Fig. 3a, b). Interestingly, OXPHOS-associated genes were also significantly enriched (Fig. 2a, b and Supplementary Fig. 3a, b). Since ISG15 has been previously associated with mitochondria^29,30^, and based on our published findings associating PaCSC stemness with mitochondrial respiration^10^, we FACS separated PDX-derived cells based on the expression of the CSC marker autofluorescence^23^ and mitochondrial mass using MTDR (Fig. 2c). WB analysis revealed that double-positive cells had the highest levels of mono-ISG15 and ISGylated proteins (Fig. 2d). In addition, double-positive cells expressed high *ISG15* and *KLF4* and low *CMYC* mRNA levels (Fig. 2e), correlating with established PaCSC molecular phenotypes^10,31^. Since these results suggested a possible link between ISG15 and mitochondria, mitochondria from adherent and sphere-derived cultures were enriched for and ISG15/ISGylation, in addition to mitochondria-specific proteins, were determined by WB analysis. As suspected, mitochondria from PaCSC-enriched cultures contained more mono-ISG15 and ISG15-conjugated proteins compared to mitochondria from non-CSC-enriched adherent cultures (Fig. 2f). A similar increase was observed in the cytosolic fraction, consistent with our initial observations (Fig. 1c and Supplementary Fig. 1c). Finally, when *ISG15* was edited using CRISPR-Cas9, a marked decrease in mono-ISG15 and ISG15-conjugated proteins in both the mitochondrial and cytosolic fractions was achieved (Fig. 2f).

### Loss of ISG15/ISGylation affects CSC functional properties

**Fig. 3:**
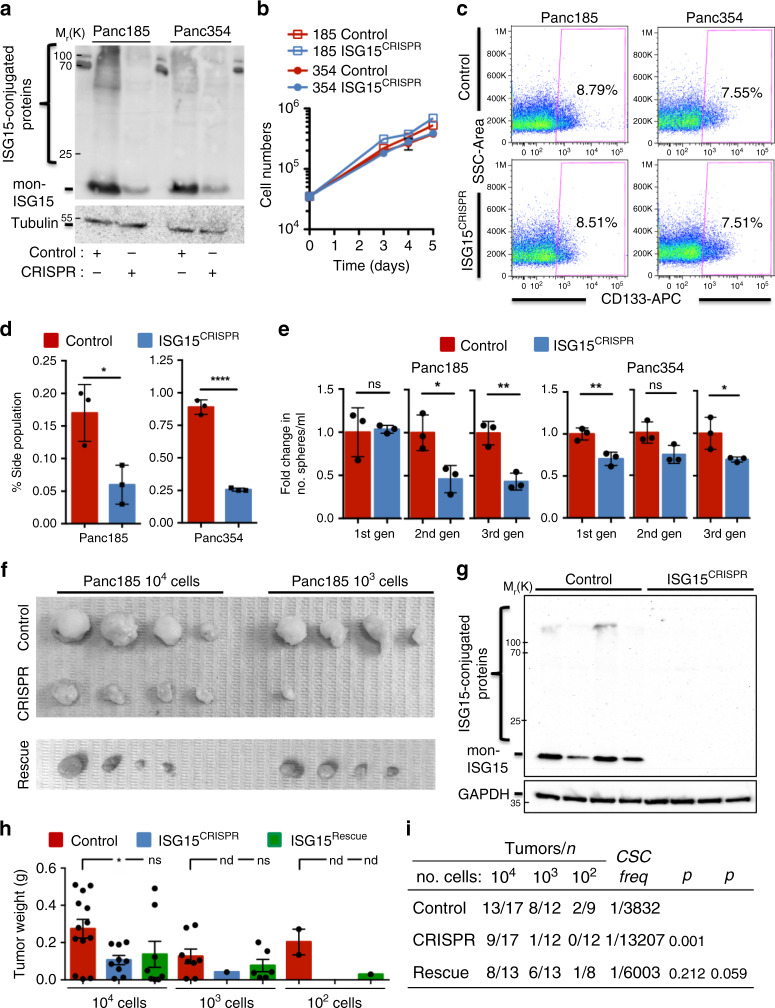
Loss of ISG15/ISGylation affects PaCSC functional properties. **a** WB analysis of mon-ISG15 and ISG15-conjugated proteins after CRISPR-Cas9﻿ ISG15 editing (CRISPR) in two different primary PDAC PDX cultures. Tubulin was used as loading control. **b** Proliferation of control and ISG15^CRISPR^ Panc185 and Panc354 cells, graphed as mean cell numbers ± sd determined at the indicated days post seeding (*n* = 3 biologically independent samples). **c** Representative flow cytometry plots for CD133 expression in control and ISG15^CRISPR^ Panc185 and Panc354 cells. **d** Mean percent of Hoechst non-retaining cells (i.e., side population) ± sd in control and ISG15^CRISPR^ Panc185 and Panc354 cells (*n* = 3 biologically independent samples; *p = 0.0227; ****p < 0.0001, as determined by Student’s *t*-test). **e** Mean fold change ± sd of 1st, 2nd, and 3rd generation (gen) sphere formation capacity of control versus ISG15^CRISPR^ cells (Panc185: *n* = 3 biologically independent samples; *p = 0.0230; **p = 0.0044; ns not significant, as determined by Student’s *t*-test; and Panc354: *n* = 3 biologically independent samples; *p = 0.0482; **p = 0.0090; ns, not significant, as determined by Student’s *t-*test). Control set as 1.0. **f** Images o**f** tumors obtained after injection of 10^4^ and 10^3^ Panc185 control, ISG15^CRISPR^ and ISG15^Rescue^ (ISG15^CRISPR^ + ISG15-V5-GFP, E1, E2, and E3 overexpression) (Rescue) cells. **g** WB analysis of mon-ISG15 and ISG15-conjugated proteins in freshly digested 10^4^ tumors obtained from **f**. GAPDH was used as loading control. **h**–**i** Average tumor weights ± sem (*n* = indicated in **i**; *p = 0.0172; ns, not significant, as determined by Student’s *t*-test; nd, not determined) (**h**) and total number of tumors obtained from control, ISG15^CRISPR^ and ISG15^Rescue^ injections from three independent in vivo ELDA animal experiments (**i**). CSCs frequencies were calculated using the Extreme Limiting Dilution Analysis software.

To assess the molecular and functional consequences of *ISG15* loss on PaCSCs, we used CRISPR-Cas9 to target *ISG15* in Panc185 and Panc354 and established stably-edited polyclonal cultures (in order to maintain the heterogeneity of the primary culture) (Fig. 3a). While the loss of ISG15 had no significant effect on proliferation, the expression of pluripotency-associated transcripts, or pERK1/2 protein levels (Fig. 3b and Supplementary Fig. 4a, b), the expression of well-established CSC markers was modulated, but varied between cell lines. The expression of the well-established marker CD133 was not affected upon ISG15 loss in Panc185 or Panc354 (Fig. 3c), while other markers, such as ALDH1, SSEA1, SSEA4 and CD24 were reduced but not always in both cell lines (Supplementary Fig. 4c). These data indicate fluctuations but not elimination of the CSC population(s) upon edition of ISG15. In contrast, when we employed a functional CSC readout assay (i.e., side population) we observed a consistent and significant reduction in both ISG15^CRISPR^ cultures (Fig. 3d). To further functionally characterize the consequence of ISG15 loss on the PaCSC population, we assessed sphere formation capacity and measured significantly lower self-renewal in ISG15^CRISPR^ cells across multiple generations (Fig. 3e).

ISG15 has both autocrine and paracrine functions^32^. We reported that PaCSCs can secrete free ISG15, which may act directly on PaCSCs via an autocrine mechanism of action, or on other TME cells via paracrine signaling^22^. Indeed, when PaCSCs were treated with recombinant ISG15 (rISG15), we previously observed increased self-renewal capacity, pERK1/2 signaling and intracellular ISGylation^22^; however, whether rISG15 entered directly into the cell to ISGylate proteins or acted through a receptor-mediated signaling cascade was not determined. Importantly, neither the self-renewal capacity of Panc185-ISG15^CRISPR^ cells, intracellular ISGylation or pERK activation was restored following treatment with rISG15 (Supplementary Fig. 5a–d), indicating that rISG15 signaling is likely receptor-mediated and its ability to enhance ISGylation is dependent on endogenous ISG15. Thus, loss of ISGylation rather than loss of ISG15 autocrine signaling negatively impacts PaCSC functional properties, as free ISG15 cannot rescue ISG15^CRISPR^ cells.

Tumorigenesis is a hallmark of CSCs, and consistent with reduced sphere formation capacity, Panc185-ISG15^CRISPR^ cells exhibited significantly reduced tumor growth and a marked reduction in tumor take when <10,000 cells were injected (Fig. 3f). Of note, the Panc185-ISG15^CRISPR^ tumors that did form with 10,000 or 1000 cells were not a result of outgrowth of ISG15-positive cells as demonstrated by WB analysis of human ISG15 performed on explanted tumors (Fig. 3g and Supplementary Fig. 6a). Importantly, similar results were obtained with Panc354-ISG15^CRISPR^ cells (Supplementary Fig. 6b, c). We then performed in vivo extreme limiting dilution assays (ELDA) with 10,000, 1000 and 100 cells, and calculated a 3.45-fold reduction in CSC numbers in the absence of ISG15 (Fig. 3i). This reduction in tumor numbers, sizes and weights coincided with reduced kinetics in tumor take, with ISG15^CRISPR^ tumors appearing later in time (Supplementary Fig. 6d). Finally, the reduced tumorigenic potential of Panc185-ISG15^CRISPR^ cells could be partially rescued by overexpression of a V5-tagged ISG15 construct (Fig. 3f, i and Supplementary Fig. 6e). Altogether, these findings indicate a central role for ISG15/ISGylation in the maintenance of PaCSCs in vivo.

To identify key cellular pathways altered by ISG15 loss, RNAseq analysis was performed on ISG15^CRISPR^ sphere-derived cells. We observed downregulation of genes belonging to key CSC pathways, including MYC, OXPHOS, MTORC1, P53, IL-6/JAK/STAT3, as well as EMT signaling (Supplementary Fig. 7a, b). Interestingly, genes associated with KRas, Hedgehog, TGF-β and WNT signaling were significantly increased (Supplementary Fig. 7a). RNA-seq analysis of ISG15^CRISPR^ cells highlighted a link between ISG15 and EMT, mitochondria and mitochondrial metabolism. We have previously shown that macrophage conditioned media (MCM) from M2-polarized macrophages can induce a mesenchymal transition in PaCSCs at the morphological and transcriptional level^22^. Along these lines we observed that treatment of PDAC cells with MCM also increases *ISG15* mRNA levels, suggesting that *ISG15* expression may play a role in PDAC EMT, invasion and/or migration (Supplementary Fig. 8a). EMT-associated morphological, transcriptional and migratory changes were evaluated in control and ISG15^CRISPR^ cells in the presence of EMT promoting factors. Loss of ISG15 had no effect on the capacity of cells to assume a more elongated mesenchymal morphology, nor was there a consistent effect on the expression of EMT genes or migration in the presence of MCM or oncostatin M (Supplementary Fig. 8b–e). Thus, ISG15 seems not to be an EMT master regulator and its loss does not abrogate the capacity of PDAC cells to undergo EMT.

### Loss of ISG15/ISGylation alters mitochondrial metabolism

**Fig. 4:**
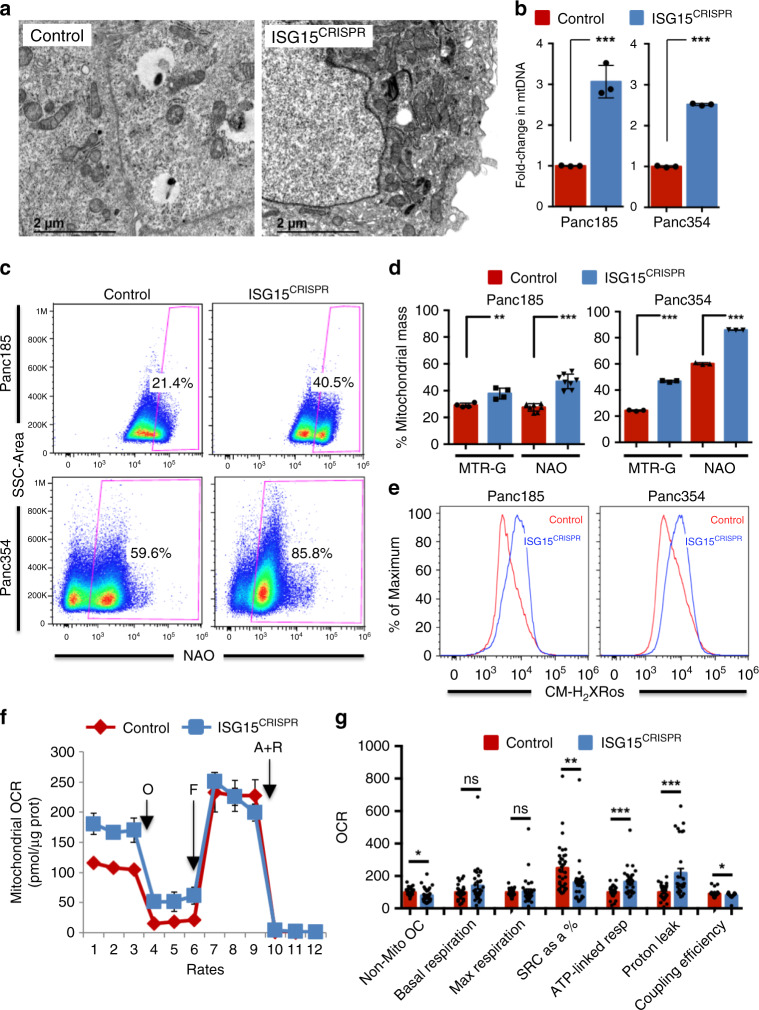
Loss of ISG15/ISGylation alters mitochondrial state and metabolism. **a** Transmission electron micrographs of control and ISG15^CRISPR^ Panc185 cells. Scale bars = 2 µM. **b** RTqPCR analysis of fold-change in mitochondrial DNA (mtDNA) gene 12s mean copies ± sd in control and ISG15^CRISPR^ Panc185 and Panc354 cells. Data are normalized to β-Actin expression and control set as 1.0. (*n* = 3 biologically independent samples; ***p < 0.0001, as determined by Student’s *t*-test). **c**, **d** Representative histograms of flow cytometric analysis of NAO (10-N-Nonyl acridine orange) in control and ISG15^CRISPR^ Panc185 and Panc354 cells **c**, and mean percentages ± sd in mitochondrial mass as a function of Mitotracker Green (MTR-G) and NAO (10-N-Nonyl acridine orange) staining **d** (Panc185: MTR-G *n* = 4 biologically independent samples **p = 0.0092 and NAO *n* = 8 biologically independent samples ***p < 0.001, as determined by Student’s *t*-test; and Panc354: MTR-G and NAO *n* = 3 biologically independent samples ***p < 0.001, as determined by Student’s *t*-test). **e** Representative histograms of Mitotracker CM-H_2_XRos in control and ISG15^CRISPR^ Panc185 and Panc354 cells. **f** Representative plot of mean Oxygen Consumption Rate (OCR) levels ± sem, normalized to protein content, for control and ISG15^CRISPR^ Panc185 cells, which were treated with O (oligomycin), F (FCCP), A (antimycinA) and R (rotenone) into culture medium. Continuous OCR values (pmoles/min/µg protein) are shown. **g** Measured and mean calculated parameters of OCR ± sem (*n* = 6 measurements per time point examined over five independent experiments; *p = 0.0148; **p = 0.0088; ***p < 0.001; *p = 0.0366; ns, not significant, as determined by Student’s *t*-test).

We next turned our attention to the mitochondria. Transmission electron microscopy (TEM) images of PaCSC-enriched sphere cultures consistently revealed more mitochondria per cell in ISG15^CRISPR^ cells compared to control cells, suggesting an increase in mitochondria numbers upon ISG15 loss (Fig. 4a). The latter was quantified by RTqPCR analysis of mtDNA, in which ISG15^CRISPR^ cells contained 2- to 3.5-fold more mtDNA compared to control cells (Fig. 4b). Next, total mitochondrial mass was determined using 10-N-Nonyl acridine orange (NAO), MitoTracker™ Green (MTR-G) (Fig. 4c, d and Supplementary Fig. 9a), or MitoTracker™ Deep Red (MTDR) (Supplementary Fig. 9b, c), and membrane potential was assessed using MitoTracker™Red (MTR) MTR CM-H_2_XRos (Fig. 4e) or MTR CMX-Ros (Supplementary Fig. 9d). While flow cytometric analysis using NAO or MTDR and CM-H_2_XRos revealed significantly higher mitochondrial mass and membrane potential, respectively, in ISG15^CRISPR^ cells, when the ratio of mitochondrial membrane potential/mass was calculated to normalize for the higher NAO or MTDR staining in ISG15^CRISPR^ cells, the membrane potential in PaCSC-enriched sphere cultures was lower or not significantly higher (Supplementary Fig. 9e, f). Thus, while ISG15 loss increases mitochondrial numbers and mass, the overall potential of these mitochondria does not increase as a consequence, suggesting dysfunctional mitochondria.

To functionally validate these findings, we measured the oxygen consumption rates (OCR) of control and ISG15^CRISPR^ spheres in the presence or absence of distinct inhibitors of mitochondrial function (Fig. 4f, g). Firstly, non-mitochondrial OC was significantly lower, and baseline OCR was slightly increased in sphere-derived ISG15^CRISPR^ cells compared to their control counterparts (Fig. 4f, g). We attributed the latter to increased activity but not to increased levels of OXPHOS complexes (Supplementary Fig. 10a, b). Secondly, maximal respiration (i.e., FCCP-stimulated respiration) was not significantly higher in control versus ISG15^CRISPR^ spheres (Fig. 4g). Using these parameters to determine the spare respiratory capacity (SRC) (i.e., the difference between maximal respiration and basal OCR), we observed that SRC was significantly reduced in ISG15^CRISPR^ cells, indicating that ISG15^CRIPSR^ cells are less able to overcome ATP demands under different types of mitochondrial stress, including oxidative stress, as previously reported by ref. ^33^. Taken together, these data indicated that while ISG15^CRISPR^ cells contain more mitochondria, they are highly dysfunctional. In support of this claim, ATP-linked respiration was significantly higher in ISG15^CRISPR^ cells. This could be a consequence of the cells attempting to confront an increased energy demand caused by oxidative damage/stress, which is characterized by the overproduction of reactive oxygen species (ROS) that can damage the mitochondrial respiratory chain, alter membrane permeability, and reduce ATP production, resulting in higher ATP-linked respiration as a compensatory mechanism^34^. ATP and ROS were measured in control and ISG15^CRISPR^ spheres, and in line with the aforementioned hypothesis, ATP levels were not significantly higher in light of increased ATP-linked OCR (Supplementary Fig. 11a). However, ROS levels were significantly higher in ISG15^CRISPR^ cells (Supplementary Fig. 11b), but no increase in apoptosis was observed in these cells at any time point analyzed (Supplementary Fig. 11c). Of note, while the increase in ROS may have affected MTR-G levels shown in Fig. 4c^35^, NAO is not affected by ROS, confirming an increase in mitochondrial mass in ISG15^CRISPR^ cells. In addition, proton leak was also significantly higher in ISG15^CRISPR^ cells (Fig. 4g), which is an additional indicator of decreased mitochondrial efficiency, and a consequence of mitochondria responding to increasing energy demands under oxidative damage/stress. Moreover, the decrease in coupling efficiency observed in ISG15^CRISPR^ cells is a reflection of the observed increase in proton leak^33^ (Fig. 4g). Lastly, we also observed decreased glycolysis and significantly reduced glycolytic capacity in ISG15^CRISPR^ cells (Supplementary Fig. 12a, b). Altogether, these data strongly indicate that loss of ISG15/ISGylation contributes to the accumulation of dysfunctional mitochondria and impacts the PaCSC mitochondrial and metabolic state.

### Loss of ISG15/ISGylation reduces mitophagy

**Fig. 5:**
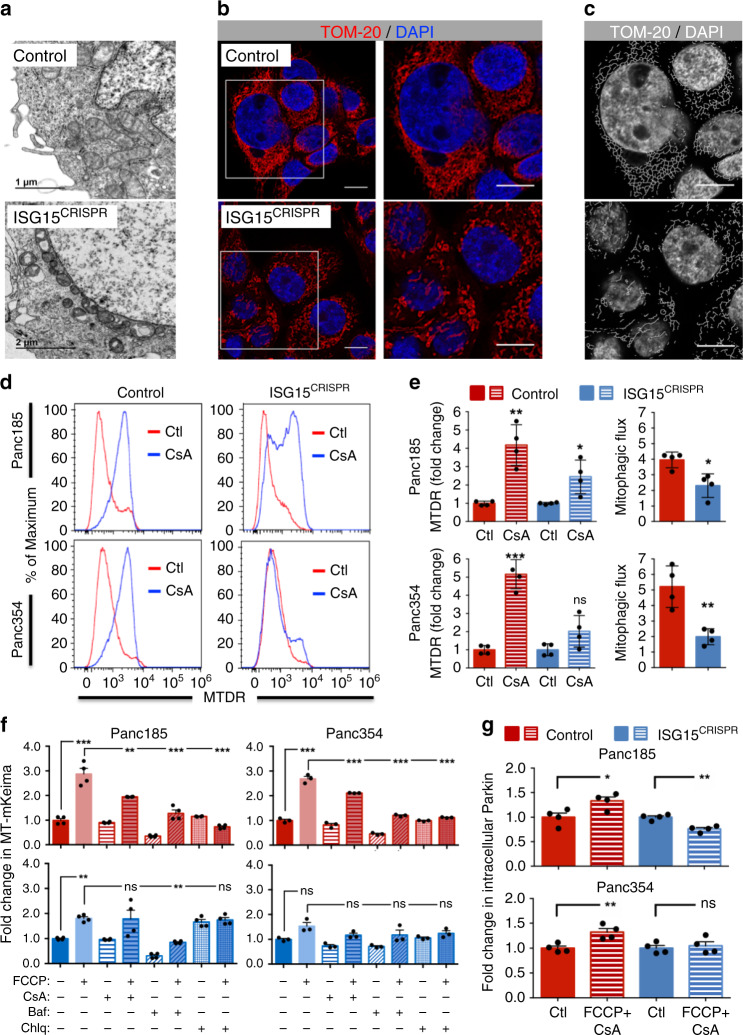
Loss of ISG15/ISGylation reduces mitophagy. **a** Transmission electron micrographs of control and ISG15^CRISPR^ Panc354 cells. Scale bars = 1 or 2 µM. **b** Immunofluorescent images of TOM-20 in control and ISG15^CRISPR^ Panc354 cells. Nuclei stained with DAPI in blue. Scale bars = 10 µM. **c** Mitochondrial network from **b** displayed as a skeleton network using ImageJ skeleton filter. Scale bar = 10 µM. **d** Representative histograms of MTDR expression in control and ISG15^CRISPR^ Panc185 and Panc354 cells after 6 h of treatment with CsA (5 µM) or without (Ctl). **e** Mean fold change in MTDR levels ± sd in control (Ctl)- and CsA-treated cultures (left) and mitophagic flux determined as the ratio of MTDR accumulation after CsA treatment compared to its respective control (right) in control and ISG15^CRISPR^ Panc185 and Panc354 cells (Panc185: *n* = 4 biologically independent samples; **p = 0.0013; *p = 0.0191; *p = 0.0111, as determined by Student’s *t*-test; and Panc354: *n* = 4 biologically independent samples; ***p < 0.001; ns, not significant; **p = 0.0041, as determined by Student’s *t*-test). **f** Mean fold change in Keima protein fluorescence levels ± sem in control (red) and ISG15^CRISPR^ (blue) Panc185 and Panc354 cells. Cells were untreated or treated with FCCP (16 µM), with or without co-treatment with the autophagy inhibitors CsA (5 µM), Bafilomycin (Baf; 150 nM) or Chloroquine (Chlq; 50 µM). Fluorescence in control-treated cells is set to 1.0. (*n* = 4 biologically independent samples for Panc185 and *n* = 3 biologically independent samples for Panc354; **p < 0.01; ***p < 0.001, ns, not significant, as determined by one-way ANOVA with Dunnett’s multiple comparison test). **g** Summary of average fold change in parkin levels ± sem, determined by flow cytometric analysis, in control and ISG15^CRISPR^ Panc185 and Panc354 cells untreated (Ctl) or treated with FCCP (16 µM) plus CsA (5 µM) for 4 h (Panc185: *n* = 4 biologically independent samples; *p = 0.0283; **p = 0.0013, as determined by Student’s *t*-test; and Panc354: *n* = 4 biologically independent samples **p = 0.0084; ns, not significant, as determined by Student’s *t*-test).

The accumulation of dysfunctional mitochondria was confirmed by several approaches. First, TEM images confirmed that mitochondria in ISG15^CRISPR^ cells were perinuclear, smaller and contained deformed cristae compared to controls that contained larger mitochondria with enlarged inner compartments and well-defined cristae (Fig. 5a and Supplementary Fig. 13a). Immunofluorescence analysis of TOM-20-stained cells revealed severe mitochondrial fragmentation indicating increased mitochondrial fission (Fig. 5b, c and Supplementary Fig. 13b), which is a precursor to mitophagy and an innate cellular response to aged or dysfunctional mitochondria^36^. We reasoned that mitophagy-dependent elimination of damaged mitochondria was impaired in the absence of ISG15. Thus, mitophagic flux was assessed using cyclosporine A (CsA), an inhibitor of mitochondrial cyclophilin D that is commonly used to suppress mitophagy^37–41^, together with MTDR staining, the latter of which should increase upon inhibition of mitophagy (Fig. 5d, e and Supplementary Fig. 13c). In control cells, CsA treatment for 6 h increased MTDR, which was divided by basal MTDR levels to calculate the mitophagic flux, as previously described^37^. In ISG15^CRISPR^ cells, however, both the increase in MTDR following CsA treatment and the mitophagic flux were significantly reduced, confirming dysfunctional mitophagy (Fig. 5d, e). To more rigorously evaluate mitophagy under forced conditions, FCCP was used to induce mitophagy^42^ and CsA, bafilomycin or chloroquine was added to inhibit mitophagy at different levels/steps. For these experiments, we transfected control and ISG15^CRIPSR^ cells with pMitoKeima, a plasmid expressing the fluorescent protein Keima tagged with a mitochondria targeting signal peptide sequence, whose excitation spectrum is altered by pH, indicative of mitophagosome fusion with a lysosome. Keima fluorescence was measured by flow cytometry in sorted pMitoKeima-positive cells, as previously described^43^. In line with the mitophagic flux results, we observed that FCCP efficiently induced mitophagy in control cells and when combined with the aforementioned inhibitors, Keima fluorescence dropped. In the absence of ISG15, FCCP minimally induced mitophagy and the effect of the inhibitors was marginal to none (Fig. 5f), confirming that mitophagy is impaired in the absence of ISG15.

Mitophagy is a complex process governed by several proteins, including PTEN-induced putative kinase 1 (*PINK1*) and E3 ubiquitin-protein ligase parkin (*PRKN*)^44^. Notably, a 2016 study by Im et al.^29^ showed that ISGylation of parkin positively regulates its ubiquitin E3 ligase activity. First, using a validated anti-parkin antibody (Supplementary Fig. 14a), parkin overexpression in PaCSCs was confirmed by flow cytometry (Supplementary Fig. 14b). Next, parkin expression and intracellular localization in the absence of ISG15 was evaluated by IF. There was no apparent visual difference in the levels or localization of parkin in Panc185 control or ISG15^CRISPR^ cells (Supplementary Fig. 14c, left); however, in Panc354-ISG15^CRISPR^ cells, the intracellular distribution and levels of parkin appeared reduced compared to control cells (Supplementary Fig. 14c, right). We confirmed the latter by flow cytometry (Supplementary Fig. 14d). We hypothesized that the observed differences were due to accelerated parkin protein turnover in Panc354-ISG15^CRISPR^ cells, and experimentally showed that for both Panc185 and Panc354, parkin degradation was accelerated in the absence of ISG15 (Supplementary Fig. 14e). To more rigorously evaluate parkin protein dynamics under forced conditions, FCCP was used to induce both mitophagy and parkin mitochondrial recruitment^42^, and CsA was added to inhibit mitophagy-mediated elimination of mitochondria-bound parkin. Flow cytometric analysis confirmed an increase in parkin expression in control cells treated with both FCCP and CsA; however, in ISG15^CRISPR^ cells, parkin expression was either unchanged or significantly decreased (Fig. 5g), confirming reduced induction and accumulation of parkin on mitochondria in ISG15^CRISPR^ cells.

### Autophagosomes and autophagic flux increase in the absence of ISG15/ISGylation

**Fig. 6:**
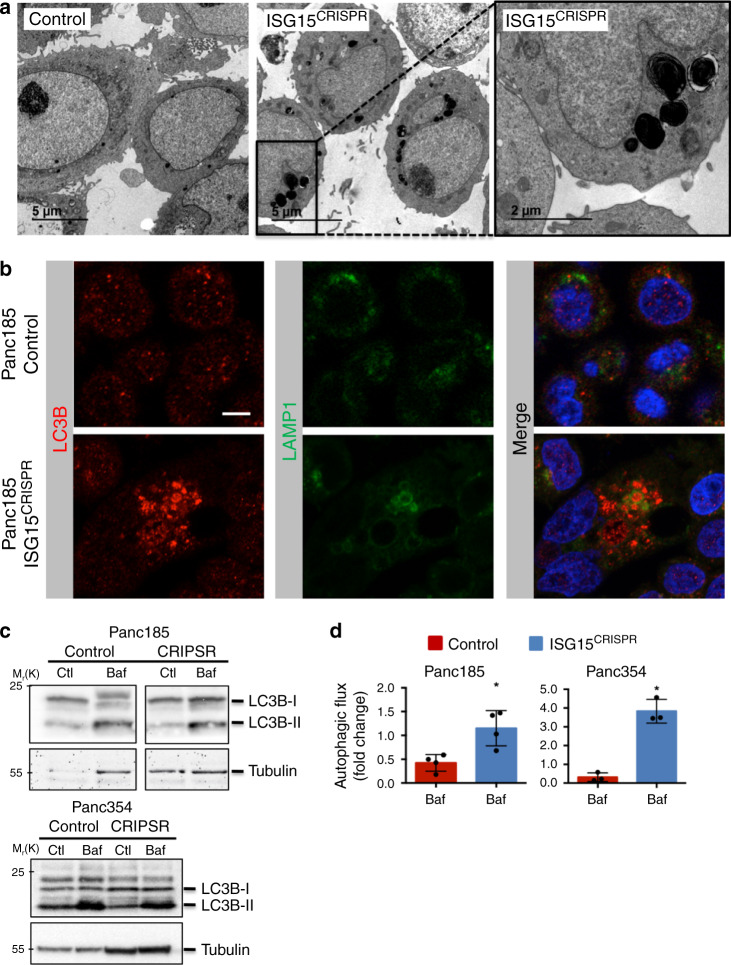
Autophagosomes and autophagic flux increase in the absence of ISG15/ISGylation. **a** Transmission electron micrographs of control and ISG15^CRISPR^ Panc185 cells. Scale bars = 5 or 2 µM. **b** Immunofluorescent images of LC3B (red) and LAMP1 (green) in control and ISG15^CRISPR^ Panc185 cells. Nuclei stained with DAPI in blue. Scale bar = 10 µM. **c** WB analysis of LC3B in control and ISG15^CRISPR^ cultures untreated (Ctl) or treated with the autophagy inhibitor Bafilomycin (Baf) for 5 h. Tubulin was used as loading control. **d** Mean fold change ± sd in autophagic flux determined by densitometric quantification of LC3B-II accumulation after autophagy inhibitor addition, subtracting their respective control expression levels (Panc185: *n* = 4 independent experiments; *p = 0.0162, as determined by Student’s *t*-test; and Panc354: *n* = 3 independent experiments; *p = 0.0143, as determined by Student’s *t*-test).

TEM micrographs of control and ISG15^CRISPR^ PaCSCs also revealed the presence of numerous cytoplasmic vesicles that contained highly electron-dense materials (Fig. 6a and Supplementary Fig. 15a) in the absence of ISG15. As the structures were reminiscent of phagolysosomes, we performed IF analysis for the autophagy marker microtubule-associated protein light chain 3 (LC3) and the lysosome marker LAMP1, and observed LC3B and LAMP1 association and enrichment in ISG15^CRISPR^ cells (Fig. 6b). In addition, we used lysotracker and acridine orange (lysosome-specific probes) and observed a consistent and reproducible increase in lysosomes in the absence of ISG15 (Supplementary Fig. 15b). While these qualitative approaches merely suggested altered autophagy in ISG15^CRISPR^ cells, the autophagic flux in control and ISG15^CRISPR^ cells was calculated based on the amount of LC3B-II accumulation in the presence or absence of the lysosomotropic reagent bafilomycin A1 (Baf), which prevents the fusion of the autophagosome to the lysosome and their subsequent degradation. LC3B-II accumulation in the presence of Baf was greater in ISG15^CRISPR^ cells compared to control cells (Fig. 6c and Supplementary Fig. 15c), which translated to significantly higher autophagic flux (Fig. 6d) and indicated that ISG15^CRISPR^ cells have modulated autophagy.

### Loss of ISG15/ISGylation prevents PaCSC metabolic plasticity

**Fig. 7:**
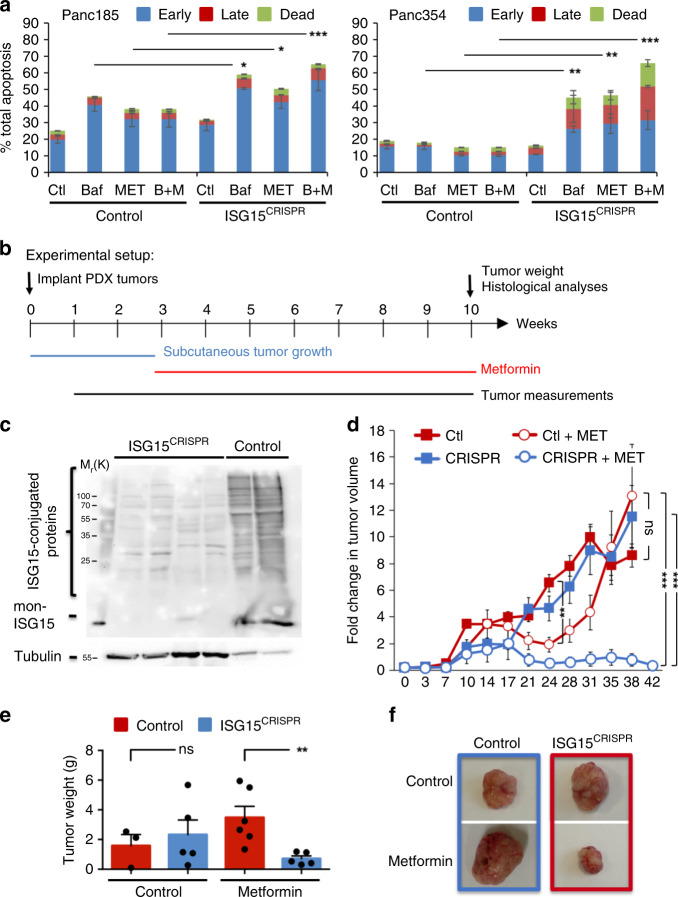
Loss of ISG15/ISGylation prevents PaCSC metabolic plasticity. **a** Quantification of the average percentage ± sd of cells in early apoptosis (Annexin-V + ), late apoptosis (Annexin-V + /DAPI + ) and dead (DAPI + ) from control and ISG15^CRISPR^ sphere cultures after 3 days of treatment with Bafilomycin (1 nM, Baf, B) and/or Metformin (10 µM, MET, M) (*n* = 3 biologically independent samples; *p < 0.05; **p < 0.01; ***p < 0.001, as determined by Student’s *t-*test). **b** Experimental set-up for in vivo experiments using subcutaneously implanted control and ISG15^CRISPR^ tumors (xenografts) and treatment with Metformin (MET). **c** WB analysis of mon-ISG15 and ISG15-conjugated proteins in freshly digested donor tumors obtained from control and ISG15^CRISPR^ injected cells, used for tumor implantations in **b**. **d** Average fold change in tumor volume ± sem in control and ISG15^CRISPR^ xenografts with or without Metformin (MET) treatment over the course of 42 days (*n* = 6 tumors/group examined over two independent in vivo animal experiments; ***p < 0.001; ns, not significant, as determined by Student’s *t*-test). **e** Average tumor weights ± sem obtained at sacrifice from **d** (**p = 0.0099; ns, not significant, as determined by Student’s *t*-test). **f** Representative images of tumors at the end of the experiment.

We previously showed that while PaCSCs are initially susceptible to the mitochondrial inhibitor metformin, metabolic plasticity allows for metformin resistant cells to emerge^10^. Having observed defective mitophagy and (perhaps compensatory) increased autophagy, we set out to determine the susceptibility of ISG15^CRISPR^ cells to inhibitors of both autophagy and mitochondrial respiration. Following treatment of PaCSC-enriched sphere cultures with Bafilomycin and/or Metformin, we observed that cells lacking ISG15 expression showed increased sensitivity to autophagy and mitochondrial respiration inhibitors (Fig. 7a). As plasticity is best assessed in vivo, we implanted nude mice with control and ISG15^CRISPR^ xenografts and assessed their susceptibility to grow over the course of 6 weeks in the presence of Metformin (Fig. 7b, c). In line with our previously published work^10^, the growth of control tumors was initially impaired in mice receiving metformin, but resistance quickly occurred and control tumors became resistant to metformin treatment. To our surprise, in the presence of metformin, ISG15^CRISPR^ xenograft growth was halted, no resistance developed and sustained disease was achieved throughout the course of the study, indicating that loss of ISG15 restrains PaCSC metabolic plasticity (Fig. 7d–f).

## Discussion

Mitochondrial function is critical and as with stem cells, likely plays an important role in CSC biology^8^, by controlling numerous cellular signaling pathways^45^ and regulating stemness properties and cellular fate^16^. Herein, we build upon the role and association between mitochondrial dynamics/state and PaCSCs, and report a previously unrecognized link between ISG15/ISGylation, mitophagy and metabolic plasticity. While an increasing number of studies suggest that alterations in mitochondrial dynamics and mitophagy can severely impact (stem) cell phenotypes^16–18,46^, less is known regarding the role of mitophagy in CSCs; however, it appears as though mitophagy is also a critical biological process for CSCs^47^. Ou and colleagues^48^ recently showed in hepatocellular carcinoma that mitophagy induction increases the hepatic CSC population while its inhibition decreases it. Likewise, Pei et al.^49^ demonstrated that mitophagy is required for the self-renewal of human acute myeloid leukemia stem cells. Thus, the role of mitophagy in CSCs may be more biologically important than previously recognized.

Mitochondrial dynamics and metabolism are governed by PTMs, of which the UbL modifier ISG15 appears to be a critical regulator in PaCSCs. In the present study we show that (1) ISG15 expression is significantly higher in PDAC tumors versus adjacent healthy tissue, (2) ISG15 expression is higher in basal tumors and can be used to predict survival, (3) circulating ISG15 correlates with disease staging, and (4) PDAC patients with high ISG15 levels showed increased expression of genes associated with CSCs pathways, including EMT and OXPHOS. Indeed, across panels of PDX-derived primary cultures, and regardless of the separation/enrichment method utilized, ISG15 levels and ISGylation were consistently upregulated in PaCSCs, which we determined to be due to innate activation of the IFN pathway (JAK/STAT signaling) in PaCSCs. This is not the first time that IFN signaling has been associated with PDAC tumors^50^ or PaCSCs^22^, highlighting the importance of IFN signaling in PDAC. Of note, we observed a distinct pattern of ISGylation in PaCSCs sorted for autofluorescence compared to other separation methods. While monomeric ISG15 increased in autofluorescent-positive PaCSCs, ISG15-conjugated proteins were less abundant, suggesting that while the levels of ISG15/ISGylation is higher in PaCSCs as a whole, their likely exists subpopulations of PaCSCs that, when compared, differ at the level of monomeric ISG15, ISG15-conjugated proteins, or both.

Importantly, inhibition of ISG15/ISGylation impaired the self-renewal and tumorigenic potential of PaCSCs, and this effect could be partially rescued by overexpressing ISG15, but not by treating PaCSCs with rISG15, indicating that ISGylation and not autocrine monISG15 signaling is driving the observed effects. Interestingly, loss of ISG15/ISGylation also had differing effects on the expression of CSC markers, such as CD133, SSEA1, CD24, or ALDH1. For example, CD133 levels were not affected, while ALDH1 levels were only reduced in Panc354 cells. However, when cultures were subjected to functional CSC assays, such as Hoechst retention (i.e., side population), sphere formation or tumorigenesis, ISG15 was determined to be necessary. We conclude that ISG15 loss does not eliminate the CSC population, but rather abolishes their functional properties rather than their molecular phenotypes (expression of CSC markers or pluripotency-associated genes). Thus, unless placed in an environment that requires a functional stem-like behavior, the loss of ISG15 has no dominant phenotype.

RNAseq analysis revealed a significant decrease in key stem pathways upon loss of ISG15/ISGylation, including EMT and OXPHOS. EMT has been linked to stemness, and Desai et al.^51^ have shown that aberrant activation of the ISG15 pathway confers a motile phenotype to breast cancer cells. Likewise, Farrell et al.^52^ showed that ISG15 overexpression in Madin-Darby kidney epithelial cells promotes cell scattering and an elongated morphology. In this study, however, no conclusive link between ISG15, EMT and PaCSC stemness was observed. Instead, a correlation to mitochondrial metabolism, specifically OXPHOS, was established and confirmed through a series of metabolic-based assays. While loss of ISG15 increased mitochondrial numbers, mass and potential, OCR parameters were severely impaired in the absence of ISG15. Specifically, the SRC capacity was significantly lower in ISG15^CRISPR^ cells, indicating that these mitochondria were metabolically and energetically defective (i.e., dysfunctional).

Cells use mito-fusion, mito-fission, and mitophagy to constantly remodel the mitochondrial network and to isolate and selectively eliminate aged, superfluous or unhealthy/damaged mitochondria^16,44,53^. The proper coordination of these interconnected events guarantees homeostatic balance^16,53^. Mitophagy is a complex process and involves many cellular factors^44^, with PINK1 and PARKIN representing the two most important mediators of mitophagy. We observed extreme mitochondrial fission and fragmentation in the absence of ISG15, indicating that the precursor events to mitophagy were activated; however, the completion of the mitophagic process was impaired, resulting in the inability of PaCSCs to eliminate their dysfunctional and unhealthy mitochondria. It is important to note that autophagy was increased, indicating that mitophagy-specific factors are impaired in cell lacking ISG15. While the mechanism(s) behind how ISG15 participates in mitophagy have not been elucidated in this study, we observed a potential link between ISGylation, parkin and mitophagy. We show that PaCSC mitochondria are ISGylated, PaCSCs express higher levels of parkin compared to non-CSCs, and in the absence of ISG15, the long-term stability of parkin is modulated. During mitophagy, parkin binds to dysfunctional mitochondria and it has been previously shown that ISGylation of parkin positively regulates its ubiquitin E3 ligase activity^29^. In our experiments, we show that during induced mitophagy with FCCP, parkin levels do not increase in the absence of ISG15. Thus, we hypothesize that parkin cannot effectively complete the mitophagic process either due to its instability or decreased E3 ligase activity in cells lacking ISG15. Further experiments are necessary to validate this hypothesis. Nonetheless, understanding that mitophagy is an important quality control mechanism necessary for the recycling of OXPHOS-active mitochondria, PaCSCs preferentially use mitochondrial OXPHOS to meet their energy requirements and oncogenic transformation increases cellular stress and mitochondrial damage, then it is plausible to assume that activation of mitophagy and the clearance of dysfunctional mitochondria is an important requisite for the maintenance of the PaCSC pool. Thus, its inhibition would, at a minimum, affect CSC functionality and perhaps other key processes such as plasticity.

In addition to OXPHOS, glycolysis as well as glycolytic capacity and reserve were also significantly impaired in PaCSCs in the absence of ISG15, suggesting that the overall metabolic plasticity of PaCSCs (aerobic and anaerobic respiration) was affected by ISG15/ISGylation loss. Indeed, the latter was shown in vivo using metformin. While we have previously shown that PaCSCs are sensitive to metformin, resistance inevitably occurs due to the presence of plastic CSCs capable of shifting their metabolism towards glycolysis^10^. In the absence of ISG15, however, tumors were unable to proliferate and acquire resistance to metformin, which confirms that loss of ISG15 impedes the metabolic plasticity of PaCSCs. Thus, this study highlights a direct link between ISG15/ISGylation and metabolic plasticity in PaCSCs, and strongly argues for further research into the development of inhibitors of ISG15/ISGylation for PDAC. It is important to note that our silencing strategy does not discriminate between mono-ISG15 and ISGylation, thus further studies are still needed to determine whether the observed effects are due to mono-ISG15, ISGylation or both. Finally, it is likely that other UbL modifiers are also important for the biology of PaCSCs and that ISG15/ISGylation may also be affecting other key processes due to additional PTMs; therefore, the study of UbL modifiers and UbL-mediated PTMs in CSCs in general is also warranted by the work detailed in this study.

## Methods

### Cell lines and primary human pancreatic cancer cells

HPDE cells were kindly provided by Francisco Real (CNIO) and maintained in DMEM/F12 (Gibco) supplemented with B27 1:50 (Invitrogen, Thermo Fisher Scientific, Europe), 20 ng/mL bFGF (Pan-Biotech, GmbH, Aidenbach, Germany) and 50 U/mL penicillin/streptomycin (Invitrogen). PDAC patient-derived xenografts (PDAC PDX) were obtained from Dr. Manuel Hidalgo under a Material Transfer Agreement with the Spanish National Cancer Center (CNIO), Madrid, Spain (Reference no. I409181220BSMH). To establish low-passage primary PDAC PDX-derived in vitro cultures, xenografts were minced, enzymatically digested with collagenase (Stem Cell Technologies) for 60 min at 37 °C and after centrifugation for 5 min at 500 × *g*, the cell pellets were resuspended and cultured in RPMI (Invitrogen) supplemented with 10% fetal bovine serum (FBS; Invitrogen), 50 units/mL penicillin/streptomycin and fungizone (Invitrogen). PDAC PDX-derived cultures are referred to by a random number designation (e.g., Panc185 or Panc354). Primary cultures were tested for Mycoplasma at least every 4 weeks.

### Macrophages conditioned medium

For macrophage conditioned medium (MCM), human blood was obtained from healthy donors, and peripheral blood mononuclear cells (PBMCs) were isolated from buffy coats following standard protocols^22^. This study received the approval of the Committee of Ethics and Clinical Investigation of the Universidad Autónoma de Madrid, Spain. Monocytes were cultured under adherent conditions in RPMI medium (Invitrogen) supplemented with 10% FBS (Invitrogen) or human AB serum (Sigma, Spain) in a humidified atmosphere with 5% CO_2_ at 37 °C. Adherent monocytes were cultured for 7 to 10 days to allow for differentiation into macrophages.

### Gene expression data sets and GSEA analyses

The gene expression data sets used in this study are publicly available. The data set from Janky et al.^25^ was downloaded from GEO (GSE62165); the data set from Moffitt et al.^27^ was downloaded from GEO (GSE71729); the data set from Bailey et al.^28^ was included in a supplementary figure of their published work; and the META data set, containing data sets GSE15471, GSE16515, GSE22780, and GSE32688, was generated as described in ref. ^26^. The samples included in the top and bottom quartile of expression of ISG15 were compared in GSEA, using the Hallmark gene-set database. The GSEA module of the Genepattern suite from the Broad Institute was used, with 1000 permutations and FDR < 25% was considered statistically significant.

### Kaplan–Meier analysis of gene expression microarray

To analyze the prognostic value of ISG15 mRNA expression, patients were stratified based on the median value of ISG15 and survival analysis was performed with R. The two patient cohorts were compared by a Kaplan–Meier survival plot, and the hazard ratio with 95% confidence intervals and Log-rank *p*-value were calculated. The Cox Proportional Hazard regression model was used to calculate the hazard ratio. RStudio version 1.1.442 and R version 3.5.1 were used to generate Box and Whisker plots and Kaplan–Meier survival plots.

### Patient samples and ISG15 ELISA

Blood samples from PDAC patients and healthy donors were provided by the BioBank Hospital Ramón y Cajal-IRYCIS (PT13/0010/0002), integrated in the Spanish National Biobanks Network (ISCIII Biobank Register No. B.0000678) and by Dr. Alfredo Carrato from the “Collection of samples of the Familial Pancreas Cancer Registry” of the Carlos III Institute (ISCIII ref no.: C.0003953). Samples were processed following standard operating procedures with the appropriate approval of the Ethical and Scientific Committees (Control no. No. Control: DE-BIOB-73 AC65, RG.BIOB-57, and RG.BIOB-54), with informed consent and according to Declaration of Helsinki principles. ISG15 levels in serum samples were determined using the human ISG15 ELISA kit (E-EL-H0203, Elabscience) as per the manufacturer’s instructions.

### RNA-sequencing analysis

Total RNA was isolated by the guanidine thiocyanate (GTC) method using standard protocols^54^. PolyA+ RNA fraction was processed as in Illumina’s TruSeq RNA Sample Preparation v2 Protocol. The resulting purified complementary DNA (cDNA) library was applied to an Illumina flow cell for cluster generation (TruSeq cluster generation kit v5) and sequenced on the Genome Analyzer IIx with SBS TruSeq v5 reagents by following manufacturer’s protocols. RNA-seq data sets were analyzed using the tool Nextpresso^55^. Nextpresso is comprises four basic levels: 1. Quality check, 2. Read cleaning and/or down-sampling, 3. alignment, and 4. analysis (gene/isoform expression quantification, differential expression, gene-set enrichment analysis and fusion prediction). Gene signatures (Hallmark genesets) were downloaded from GSEA—Molecular Signature Database for Gene set enrichment analysis.

### Sphere formation assay

Pancreatic CSC spheres were generated by culturing primary pancreatic cancer cells (5000-20,000 cells/mL) in ultra-low attachment plates (Corning) using FBS-free DMEM/F12 (Invitrogen) supplemented with B27 1:50 (Invitrogen), 20 ng/mL bFGF (PAN-Biotech) and 50 U/mL penicillin/streptomycin (Thermo Fisher Scientific). Seven days later, spheres were harvested for subsequent assays or counted with an inverted EVOS FL microscope (Thermo Fisher Scientific) using a 10x objective with phase contrast. For serial passaging, spheres were harvested using a 40-µm cell strainer, trypsinized into single cells and re-cultured for another 7 days. Sphere counts are represented as the fold change in spheres number/mL.

### Electron microscopy analysis

PaCSC-enriched spheres were trypsinized and centrifuge for 5 min at 400 × *g*. Cell pellets were fixed using a solution of 2.5% glutaraldehyde in cacodylate buffer 0.1 M for 60 min. Cell pellets were post-fixed in osmium tetroxide, dehydrated through ascending concentrations of ethanol and embedded in epoxy resin. Ultra-thin sections were obtained at 0.1 μm, counterstained with uranyl acetate and lead citrate prior to image acquisition with a JEOL JEM1010 (100 kV) transmission electron microscope equipped with a Gatan Orius 200 SC camera. Images were processed using DigitalMicrograph (Gatan, Inc).

### Western blot analysis

Cells were harvested in RIPA buffer (Sigma) supplemented with a protease inhibitor cocktail (Roche Applied Science, Indianapolis, IN). Fifty micrograms of protein were resolved by sodium dodecyl sulfate–polyacrylamide gel electrophoresis (SDS-PAGE) and transferred to PVDF membranes (Amersham Pharmacia, Piscataway, NJ). Membranes were sequentially blocked with 1x TBS containing 5% BSA (w/v) and 0.5% Tween20 (v/v), incubated with a 1:500–1:1000 dilution of indicated antibodies (Supplementary Table 1) overnight at 4 °C, washed five times with 1x TBS containing 0.5% Tween20 (v/v), incubated with horseradish peroxidase-conjugated goat anti-rabbit or goat anti-mouse antibody (Amersham), and washed again to remove unbound antibody. Bound antibody complexes were detected with SuperSignal chemiluminescent substrate (Amersham) and images were obtained using MyECL Imager (Thermo Fisher Scientific). Densitometry histograms were obtained by measuring the intensity of the bands and normalized by their housekeeping loading control by ImageJ software. For positive ISG15 induction, Interferon (IFN) was purchased from Peprotech and used at 10 U/mL for 24 h. Blots are accompanied by the locations of molecular weight/size markers (M_r_(K)), as determined using commercially available protein ladders (Novex®Sharp Pre-Stained Protein Ladder Cat no. LC5800 or PageRuler™ Prestained Protein Ladders Cat nos. 26616 or 26619, all from ThermoFisher Scientific). Uncropped and unprocessed scans of all the main blots presented can be found in Supplementary Fig. 16.

For autophagy flux analysis, cells were treated with the lysosomotropic reagent Bafilomycin A1 (150 nM, Calbiochem) for 5 h or Chloroquine (50 µM, Sigma) for 72 h (ref. ^56^). After treatments, cells were harvested and analyzed by WB as described above. Autophagy flux compares the LC3B-II levels with and without the autophagy inhibitors and is calculated as the difference between of LC3B-II in the presence and absence of the indicated inhibitor, normalized to the indicated housekeeping protein.

### Flow cytometry and FACS

Cells were resuspended in Flow buffer [1x phosphate-buffered saline (PBS); 3% FBS (v/v); 3 mM EDTA (v/v)] before analysis with a 4-laser Attune NxT Acoustic Cytometer (Thermo Fisher Scientific). For cell surface marker expression, refer to antibodies listed in Supplementary Table 1. For Parkin intracellular staining, after treating cells with 5 µM CsA (Sigma, C 3662) and 16 µM FCCP (Sigma, C 2920) for 4 h, cells were incubated 30 min with Live/Dead Fixable Dead Cell Stain Kit (MAN0006891, Molecular Probes) for dead cell exclusion. Cells were fixed with 4% PFA in PBS for 10 min, washed and after o/n incubation at 4 °C, cells were permeabilized with methanol 90% in PBS for 30 min and then incubated with an α-hu-parkin antibody (ThermoFisher Scientific) as detailed in Supplementary Table 1. The specificity of the α-hu-parkin antibody for WB and Flow cytometric analysis was validated using 293T cells transiently transfected with a plasmid (pCDN3.1-Parkin) expressing the full coding sequence of parkin (NM_004562.3), kindly provided by Drs. Raúl Sánchez Lanzas and José González Castaño, Universidad Autónoma de Madrid, Madrid, Spain.

For Annexin-V staining, floating and attached cells were pooled and resuspended in 1x Annexin-V staining buffer containing Annexin-V-FITC diluted 1:20 (Biotium, Freemont, CA) and incubated for 20 min at room temperature prior to flow cytometric analysis. To evaluate chemoresistance to Bafilomycin (Baf) and Metformin (MET), cells were treated with 1 nM and/or 10 µM, respectively, for 3 days before Annexin-V staining. For autofluorescent detection, cells were excited with blue laser 488 nm and selected as intersection with emission filters 530/40 (BL1) and 580/30 (BL2) or, in case of sorting, emission filter for FITC. For all assays, 2 mg/mL DAPI (Sigma) was used to exclude dead cells. Data were analyzed with FlowJo 9.3 software (Tree Star Inc., Ashland, OR.). For cell sorting, a FACS Vantage SE Flow Cytometer was used and data analyzed with BD FACSDiVa software. For detection of ALDH1-positive cells, the Red-shifted Fluorescent Live Cell Probe for ALDH (AldeRed™ 588-A ALDH Detection Assay, SCR150, Sigma), was used according to the manufacturer’s instructions.

For mitochondrial mass and mitochondrial membrane potential measurement, Mitotacker Green (MTR-G, M7514, Invitrogen), 10-N-Nonyl acridine orange (NAO, A7847, Sigma Aldrich) Mitotracker Deep Red (MTDR, M22426, Invitrogen), CMX-ROS (M7512, Invitrogen) and CM-H_2_XRos (M7513, Invitrogen) were used. Probes were incubated with cells for 20 min at 37 °C at a concentration of 0.1 µM, 0.1 µM, 2 nM, 10 nM, and 100 nM, respectively, and fluorescence was detected using the filters (Ex488nm/Em530/30) BL1 for MTR-G and NAO, (Ex638nm/Em670/14) RL1 for MT-DR, (Ex561nm/Em585/16) YL1 for CMX-ROS and (Ex561nm/Em620/15) YL2 for CM-H_2_XRos. For ROS production measurement, MitoSOX (M36008, Invitrogen) was used at 1 µM for 10 min at 37 °C and detected with laser (Ex561nm/Em585/16) YL1. For lysosomes quantification, Acridine Orange (A3568, Thermo Fisher Scientific) was incubated at 1 μg/mL for 15 min at 37 °C and detected using BL1 filter (Ex488nm/Em530/30). Alternatively, Lysotracker Deep Red (L12492, Molecular probes, Life Technologies) was used at 1 nM for 30 min at 37 °C and detected using RL1 filter (Ex638nm/Em670/14).

To determine mitophagic flux by flow cytometry, after treatment of cells with CsA (Sigma, C 3662) at 5 µM for 5 h, cells were trypsinized, stained with 1 nM MTDR for 30 min and analyzed by Flow Cytometry. Mitophagy flux compares MTDR levels with and without the mitophagy inhibitor CsA and is calculated as the ratio of MTDR accumulation after CsA treatment compared to its respective control (adapted from ref. ^37^). Examples of gating strategies for all of the aforementioned cytometry-based analyses are presented in Supplementary Fig. 17.

### Side population

Human primary PDAC cells derived from sphere cultures at a concentration of 10^6^ cells/mL were incubated for 30 min in the absence or presence of the ABCG2 transporter inhibitor fumitremorgin C (5 μg/mL; Sigma) and subsequently stained with Hoechst 33342 (5 μg/mL; Sigma) at 37 °C for 2 h. Cells were washed and resuspended in cold 1x PBS. Propidium iodide (Sigma) was used to exclude dead cells. SP cells were analyzed using a CytoFLEX Beckman Coulter using filters NUV450 and NUV657 as previously described^23^.

### Recombinant ISG15 experiments

Cells were seeded as spheres, as previously described, and treated with 100 ng/mL of recombinant ISG15 (rISG15, purchased from Abcam ab173004 and resuspended to a concentration of 1 µg/µl in water). After 7 days, spheres were harvested in RIPA buffer for western blotting assay or photographed and counted.

### Immunostainings and confocal analysis

For immunofluorescence (IF) confocal microscopy, cells were seeded on glass coverslips in RPMI (Invitrogen) containing 10% FBS (Thermo Fisher Scientific) at 37 °C, 5% CO_2_. After indicated treatments and indicated time points, the medium was removed, cells were fixed with 4% PFA in PBS for 20 min at room temperature, washed with PBS, permeabilized with TritonX-100 1% in PBS for 15 min, blocked with 1% bovine serum albumin (BSA) in PBS for 1 h at room temperature and then incubated with specific antibodies (see Supplementary Table 1) in a solution of 1% BSA in PBS. The fluorescent images were collected with a laser scanning confocal microscope Zeiss 710 and analyzed using the software Leica 2009.

### Lentivirus production and cell transduction

Lentiviral particles were produced by transfection of 293T cells (Invitrogen) following a polyethylenimine (PEI)-based protocol, as previously described^57^. Briefly, 5 × 10^6^ 293T cells were co-transfected with 1 μg packaging plasmid psPAX2, 1 μg envelope plasmid pVSVG and 2 μg of the indicated backbone plasmid: pRRL_SIN_CMV_ISG15-V5_IRES_eGFP, pLX304_UBE1L (E1, EX-OL01733-LX304, GeneCopoeia) pRRL_SIN_CMV_UBE2L6_IRES_mCherry (E2), pReceiver-Lv105_Herc5 (E3, EX-Z9167-Lv105, GeneCopoeia), pCAS9 or 3 different ISG15-CRISPR plasmids (Target sequences: 27 GCTGGCGGGCAACGAATTCC, 275 GCAGCAGCACCTACGAGGTA and 318 CGCTCACTTGCTGCTTCAGG cloned into pLenti-U6-sgRNA-PGK-Neo; human ISG15 sgRNA CRISPR Lenti-vector set, K1101201, ABM, Richmond, BC, Canada) or control scrambled sgRNA CRISPR Lenti-vector (K018, ABM). After 8 h, the transfection medium was replaced with complete RPMI media and recombinant lentiviruses were harvested 48 h and 72 h later. Virus particle-containing supernatants were filtered through 0.45 µM PVDF membrane filters, aliquoted and stored at −80 °C until needed. For lentivirus transduction, PDAC cells were seeded in 6-well plates at a concentration of 3–5 × 10^5^ cells/well. One milliliter of the respective lentivirus was directly overlaid onto cells in the presence of polybrene (Sigma) at a final concentration of 8 μg/mL. After 16 h, medium was changed and after 48 h antibiotic selection was initiated. Stably transduced cells were obtained after GFP- or mCherry-positive cell sorting using a FACS Vantage SE Flow Cytometer for ISG15-V5 or UBE2L6, respectively, or after antibiotic cell selection using Blasticidin (5–10 µg/mL; Invitrogen) resistance (UBE1L or Cas9), Puromycin (1 µg/mL; Sigma) resistance (Herc5) or G418 (500 µg/mL; Invitrogen) resistance (ISG15-CRISPR or Control-CRISPR).

### pMT-mKeima-Red

The pMT-mKeima-Red plasmid was purchased from Medical & Biological Laboratories (MBL) CO., LTD., Japan (Cat no. AM-V0251) and used according to the manufacturer’s instructions. Keima-Red fluorescence observed with a short wavelength (440 nm) excitation is indicative of a neutral environment while fluorescence observed with long wavelength (586 nm) excitation is indicative of Keima being present in an acidic environment, which occurs upon mitophagosome fusion with a lysosome. For the detection of mitophagy with Keima-Red by flow cytometry, the protocol published by Um et al.^43^ was followed. Briefly, control and ISG15^CRISPR^ cells were transfected with 1 µg of pMT-mKeima-Red using the Neon Electroporation Transfection system (Thermo Fisher Scientific), according to the manufacturer’s instructions. Transfected cells were selected for with Hygromycin (200 µg/mL) until stable cell lines were established. Cells were subsequently sorted for Keima-Red fluorescense (Em 620 nm) using a FACS Vantage SE Flow Cytometer and data analyzed with BD FACSDiVa software. For mitophagy experiments, mitophagy was induced with 16 µM FCCP (Sigma, C 2920) and blocked with 5 µM CsA (Sigma, C 3662), Bafilomycin A1 (150 nM, Calbiochem) or Chloroquine (50 µM, Sigma) for 24 h. Cells were analyzed with (Ex561/Em620/15) YL2 filter (Attune NxT Acoustic Cytometer, Thermo Fisher Scientific) to measure Keima-Red fluorescence in an acidic environment, which occurs upon mitophagosome fusion with a lysosome. Data were analyzed with FlowJo 9.3 software (Tree Star Inc., Ashland, OR).

### Mitochondria and cytosol fractionation

Cells were culture in adherence or as spheres as described above. After 7 days, cells were harvested and mitochondria and cytosol fractioned according to the Mitochondria Isolation Kit protocol (89874, ThermoFisher Scientific). After isolation, mitochondria pellets were resuspended in SDS-PAGE buffer and analyzed by WB as described above.

### In vivo tumorigenicity assays

Mice were housed according to institutional guidelines and all experimental procedures were performed in compliance with the institutional guidelines for the welfare of experimental animals approved by the Universidad Autónoma de Madrid Ethics Committee (CEI 60-1057-A068) and La Comunidad de Madrid (PROEX 335/14) and in accordance with the guidelines for Ethical Conduct in the Care and Use of Animals as stated in The International Guiding Principles for Biomedical Research involving Animals, developed by the Council for International Organizations of Medical Sciences (CIOMS). Briefly, mice were housed according to the following guidelines: a 12 h light/12 h dark cycle, with no access during the dark cycle; temperatures of 65–75 °F (~18–23 °C) with 40–60% humidity; a standard diet with fat content ranging from 4 to 11%; sterilized water was accessible at all times; for handling, mice were manipulated gently and as little as possible; noises, vibrations and odors were minimized to prevent stress and decreased breeding performance; and enrichment was always used per the facility’s guidelines to help alleviate stress and improve breeding.

Female 6- to 8-week-old NU-Foxn1nu nude mice (Envigo, Spain) were subcutaneously injected with 10^2^, 10^3^, or 10^4^ PDAC cells expressing Cas9/Control sgRNA, Cas9/ISG15-CRISPR or Cas9/ISG15-CRISPR/eGFP-ISG15-V5 in 50 µl Matrigel (Corning) per injection. Tumor growth was monitored bi-weekly for up to 4 months. Mice were sacrificed and tumors were weighed, photographed, and part of each tumor was fixed in 4% PFA and processed for histological analysis or mechanically digested in RIPA buffer (Sigma) supplemented with protease inhibitor cocktail (Roche). Protein lysates were assessed for human ISG15 levels by WB analysis as described above.

For metformin experiments, tumors were initially established by injecting 10^6^ PDAC cells expressing Cas9/Control sgRNA or Cas9/ISG15-CRISPR in 50 µl Matrigel (Corning) per injection in 6- to 8-week-old NU-Foxn1nu nude mice (Envigo). Four to 5 weeks post injections, donor tumors were excised, cut into identical pieces of ~50 mm^3^ and implanted subcutaneously into the left and right flanks of NU-Foxn1nu nude mice (Envigo). Three weeks following subcutaneous tumor growth, tumors were measured, mice were randomized into treatment groups (5 mice per group) and metformin (Alfa Aesar, ThermoFisher Scientific) treatment was initiated (1 mg/mL in drinking water) and continued for 6 weeks. Tumor volumes were determined twice per week using a digital caliper, and drinking water was changed every 2 days. At the time of sacrifice, tumors were excised, weighed, photographed and fixed in 4% PFA and processed for histological analysis.

### RNA preparation and real-time qPCR

Total RNA was isolated by the GTC method using standard protocols^54^. One microgram of purified RNA was used for cDNA synthesis using the Thermo Scientific Maxima First Strand cDNA Synthesis Kit (ThermoFisher Scientific) according to manufacturer´s instructions, followed by SYBR green RTqPCR (PowerUp™ SYBR™ Green Master Mix, ThermoFisher Scientific) using an Applied Biosystems StepOnePlus™ real-time thermocycler (ThermoFisher Scientific). Thermal cycling consisted of an initial 10 min denaturation step at 95 °C followed by 40 cycles of denaturation (15 s at 95 °C) and annealing/extension (1 min at 60 °C). mRNA copy numbers were determined relative to standard curves comprised of serial dilutions of plasmids containing the target coding sequences and normalized to ß-actin levels. Primers used are listed in Supplementary Table 2.

### Proliferation assay

For proliferation assay, 35,000 cells/well were seeded in 24-well plates in RPMI containing 10% FBS at 37 °C, 5% CO_2_ and triplicate wells were counted every 24 h for 5 consecutive days.

### Oxygen consumption rate (OCR) and extracellular acidification rate (ECAR) measurements

Sphere-derived cells were plated in XF96 Cell Culture Microplates (Seahorse Bioscience) previously coated with Cell-Tak (BD Biosciences) at a cellular density of 30,000 cells/well. For OCR determination, cells were incubated in base assay medium (D5030, Sigma) supplemented with 2 mM glutamine, 10 mM glucose, and 1 mM pyruvate for 1 h, prior to the measurements using the XF Cell Mito Stress Kit (Seahorse Bioscience). After an OCR baseline measurement, the minimum oxygen consumption was determined adding 1 µM oligomycin (O) and the maximal respiration rate was assessed by adding 1 µM FCCP (F). At the end of the experiment the non-mitochondrial oxygen consumption was evaluated adding both 1 µM rotenone (R) and antimycin (A). Experiments were run in a XF96 analyzer (Seahorse Bioscience), and raw data were normalized to protein content.

### ATP determination assay

Lysate pellets of cells from control and Panc185-ISG15^CRISPR^ cells were collected to evaluate the changes in the levels of ATP. The analysis was performed using the ATP Bioluminiscense Assay Kit CLS II (Cat. no. 11699695001, Roche) according to the manufacturer’s instructions. Bioluminiscence was determined using a Synergy™ HT Multi-Mode Microplate Reader (BioTek, Winooski, Vermont, USA).

### EMT induction assays

For EMT induction, Oncostatin M (OSM) (R&D systems) was used at 100–200 ng/mL every 48 h during 4 days.

### Wound-healing assay

Cells were cultured until confluence and then wounded using a 200 µl yellow pipette tip. Cells were treated with Oncostatin M (OSM) (R&D systems) at 100–200 ng/mL every 48 h during 4 days. Three wounds were made for each sample, and migration distance was photographed and measured at time 0 h and every 12 h until 48 h.

### Protein half-life assay

The half-life of Parkin was evaluated incubating the cells with 25 µg/mL of cycloheximide (CHX) dissolved in absolute ethanol. After indicated time points, cells were harvested in RIPA buffer (Sigma) supplemented with a protease inhibitor cocktail (Roche Applied Science, Indianapolis, IN). Fifty micrograms of protein were resolved by SDS-PAGE and western blotting was performed as described above.

### Statistical and reproducibility

Pair-wise multiple comparisons were performed with one-way ANOVA (two-sided) with Bonferroni or Dunnett adjustment. Unless stated otherwise, unpaired two-sided (Confidence interval of 95%) Student’s *t*-test were used to determine differences between means of groups. *p*-values < 0.05 were considered statistically significant. All analyses were performed using GraphPad Prism version 6.0c (San Diego California USA).

The number of biologically independent samples are indicated in the figure legends. Repeated independent experiments per each panel with similar results are shown below. *n* = 1 (Figs. 1f, h, 2d–f, 3b, d, 4a, 5a, 6a, b, 7a, c, Supplementary Figs. 1d–e, 4b, 5c, d, 6a, b, e, 13a, 15a); *n* = 2 (Figs. 1b, c, 3e, g, 4b–e, 5f, g, 7d, e, Supplementary Figs. 1a–c, 5a, 6c, d, 8e, 10a, b, 11a, b, 14b, d, 15b); *n* = 3 (Figs. 3a, 5b–e, 6c, d, Supplementary Figs. 4a, 5b, 8a–d, 9a–f, 13b, c, 14a, c, e, 15c), *n* = 5 (Fig. 4f–g, Supplementary Figs. 11c, 12a, b).

### Reporting summary

Further information on research design is available in the Nature Research Reporting Summary linked to this article.

## Supplementary information

Supplementary Information

Reporting Summary

## Data Availability

RNAseq data from Control and ISG15^CRISPR^ Panc185 cells, generated in this study, have been deposited in the ArrayExpress database^58^ at EMBL-EBI (www.ebi.ac.uk/arrayexpress) under accession number E-MTAB-8984. Unique identifiers for the publicly available data sets used are indicated, and source data other than those provided in the Article or Supplementary Information are available from the corresponding author upon reasonable request.
