# Supplementary material for: ISG15 and ISGylation is required for pancreatic cancer stem cell mitophagy and metabolic plasticity
Source: Nat Commun. 2020 May 29;11:2682. doi: 10.1038/s41467-020-16395-2 (PMC7260233; doi:10.1038/s41467-020-16395-2)
Supplement: Supplementary file 1 — Supplementary Information [file 41467_2020_16395_MOESM1_ESM.pdf]

Loss of ISG15 and ISGylation Reduces Pancreatic Cancer Stem Cell Mitophagy, Stemness and Metabolic Plasticity

Sonia Alcalá, Patricia Sancho, Paola Martinelli, Diego Navarro, Coral Pedrero, Sandra Valle, Laura Martín-Hijano, Julie Earl, Macarena Rodríguez-Serrano, Laura Ruiz Cañas, Katerin Rojas, Alfredo Carrato, Laura García-Bermejo, Miguel Ángel Fernández-Moreno, Patrick C. Hermann, and Bruno Sainz, Jr.

Supplementary Table 1. Antibodies

1ª Abs-Epitope	Source	Dilution	Application	Manufacturer
α-hu-CD133/1-APC	Mouse monoclonal	1:10	FC	Miltenyi Biotec (Cat no. 130-090-826)
α-hu-ISG15	Rabbit	1:500	WB	ProteinTech (Cat no. 15981-1-AP)
α-β-ACTIN	Mouse monoclonal	1:5000	WB	ThermoFisher (Cat no.MA1-140)
α-GAPDH	Mouse monoclonal	1:5000	WB	ThermoFisher (Cat no.MA5-15738)
α-hu-LC3BI/II	Rabbit	1:500/1:100	WB/IF	Sigma (Cat no. L7543)
α-hu-LAMP1 (H43A)	Mouse monoclonal	1:100	IF	Santa Cruz (Cat no. sc-20011)
α-hu-PARKIN	Rabbit	1:500/1:200/100	WB/IF/FC	ThermoFisher (Cat no.PA5-13398)
α-hu-phospho-ERK1/2	Rabbit	1:500	WB	Cell Signalling (Cat no. 9101S)
α-hu-ERK1/2	Rabbit	1:500	WB	Cell Signalling (Cat no. 9102S)
α-TUBULIN	Mouse monoclonal	1:5000	WB	Elabscience (E-AB-20033)
α-VINCULIN	Mouse monoclonal	1:1000	WB	Sigma (SAB4200080)
α-hu-SSEA1-APC	Mouse monoclonal	1:10	FC	Miltenyi Biotec (Cat no.130-104-990)
α-hu-SSEA4-APC	Mouse monoclonal	1:50	FC	BioLegend (Cat no. 330418)
α-hu-CD24-PE	Mouse monoclonal	1:10	FC	Miltenyi Biotec (Cat no.130-108-381)
α-V5	Mouse monoclonal	1:1000	WB	Invitrogen (Cat no. R960-25)
α-hu-phospho-STAT1	Rabbit	1:1000	WB	Cell Signalling (Cat no. 9177)
α-hu-STAT1	Rabbit	1:1000	WB	Cell Signalling (Cat no. 9172)
α-hu-IRF9	Mouse monoclonal	1:500	WB	Santa Cruz (Cat no. sc-365893)
TOM-20	Mouse monoclonal	1:200	IF	Santa Cruz (Cat no. sc-17764)
α-HIS Tag	Mouse monoclonal	1:1000	WB	ThermoFisher (Cat no. MA1-21315)
Total OXPHOS Human WB Antibody Cocktail	Mouse monoclonal	1:500	WB	Abcam (Cat no. ab110411)

2ª Abs-Epitope	Source	Dilution	Application	Manufacturer
α-mouse-HRP	Sheep	1:5,000	WB	Amersham (Cat no. NA9310-1ML)
α-rabbit-HRP	Donkey	1:5,000	WB	Amersham (Cat no. NA9340-1ML)
α-mouse Alexa 647	Goat	1:500	IF	Invitrogen (Cat. no. A32728)
α-rabbit Alexa 555	Goat	1:500	IF/FC	Invitrogen (Cat. no. A32732)

WB = Western blot; FC = Flow cytometry; IF = Immunofluorescence

Supplementary Table 2. RTqPCR human primer sequences

Gene	Application	Primer sense	Primer antisense
ACTB	RTqPCR	*GCGAGCACACGAGCCTCGCCTT*	*CATCATCCATGGTGAGCTGGCGG*
ISG15	RTqPCR	*CACAGCCATGGGCTGGGACCTG*	*GCACGCCATCTTCTGGGTGA*
SUMO	RTqPCR	*TCCCTGCAGCCGCGGTGT*	*GGGGGTCTCCGCACCACT*
NEDD8	RTqPCR	*ATTACAAGATTCTAGGTGGTT*	*GAGTGAGAGGATATGTGATG*
FAT10	RTqPCR	*CAATGCTTCCTGCCTCTGTG*	*TGCCTCTTTGCCTCATCACC*
CMYC	RTqPCR	*CCCGCTTCTCTGAAAGGCTCTC*	*CTCTGCTGCTGCTGCTGGTAG*
KLF4	RTqPCR	*ACCCACACAGGTGAGAAACC*	*ATGTGTAAGGCGAGGTGGTC*
12S (mtDNA)	RTqPCR	*CCACGGGAAACAGCAGTGAT*	*CTATTGACTTGGGTTAATCGTGTGA*
E1 (UBE1L)	RTqPCR	*CTTCCAGGTGGTGGTGCTGACT*	*GGGTTCTGCCTCTGTGGGGTC*
E2 (UBE2L6)	RTqPCR	*ATGGCGAGCATGCAGTGGT*	*TGGTAGGGAGGTTGGTCGGGT*
E3 (HERC5)	RTqPCR	*TAGCTGAGGCTGCGGTTCCCC*	*CGTTGCGCCGCGACTTCCTC*
OCT3/4	RTqPCR	*CTTGCTGCAGAAGTGGGTGGAGGAA*	*CTGCAGTGTGGGTTTCGGGCA*
SOX2	RTqPCR	*AGAACCCCAAGATGCACAAC*	*CGGGGCCGGTATTTATAATC*
LOXL2	RTqPCR	*GGCACCGTGTGCGATGACGA*	*GCTGCAAGGGTCGCCTCGTT*
SNAI1	RTqPCR	*CTCCCTGTCAGATGAGGAC*	*CCAGGCTGAGGTATTCCTTG*
SNAI2	RTqPCR	*GGGGAGAAGCCTTTTTCTTG*	*TCCTCATGTTTGTGCAGGAG*
VIMENTIN	RTqPCR	*GAGAACTTTGCCGTTGAAGC*	*GCTTCCTGTAGGTGGCAATC*
ZEB1	RTqPCR	*CCAGGTGTAAGCGCAGAAA*	*CCACAATATGCAGTTTGTCTTCA*
CDH1	RTqPCR	*TGCCCAGAAAATGAAAAAGGC*	*GTGTATGTGGCAATGCGTTC*

RTqPCR = Real-Time quantitative PCR

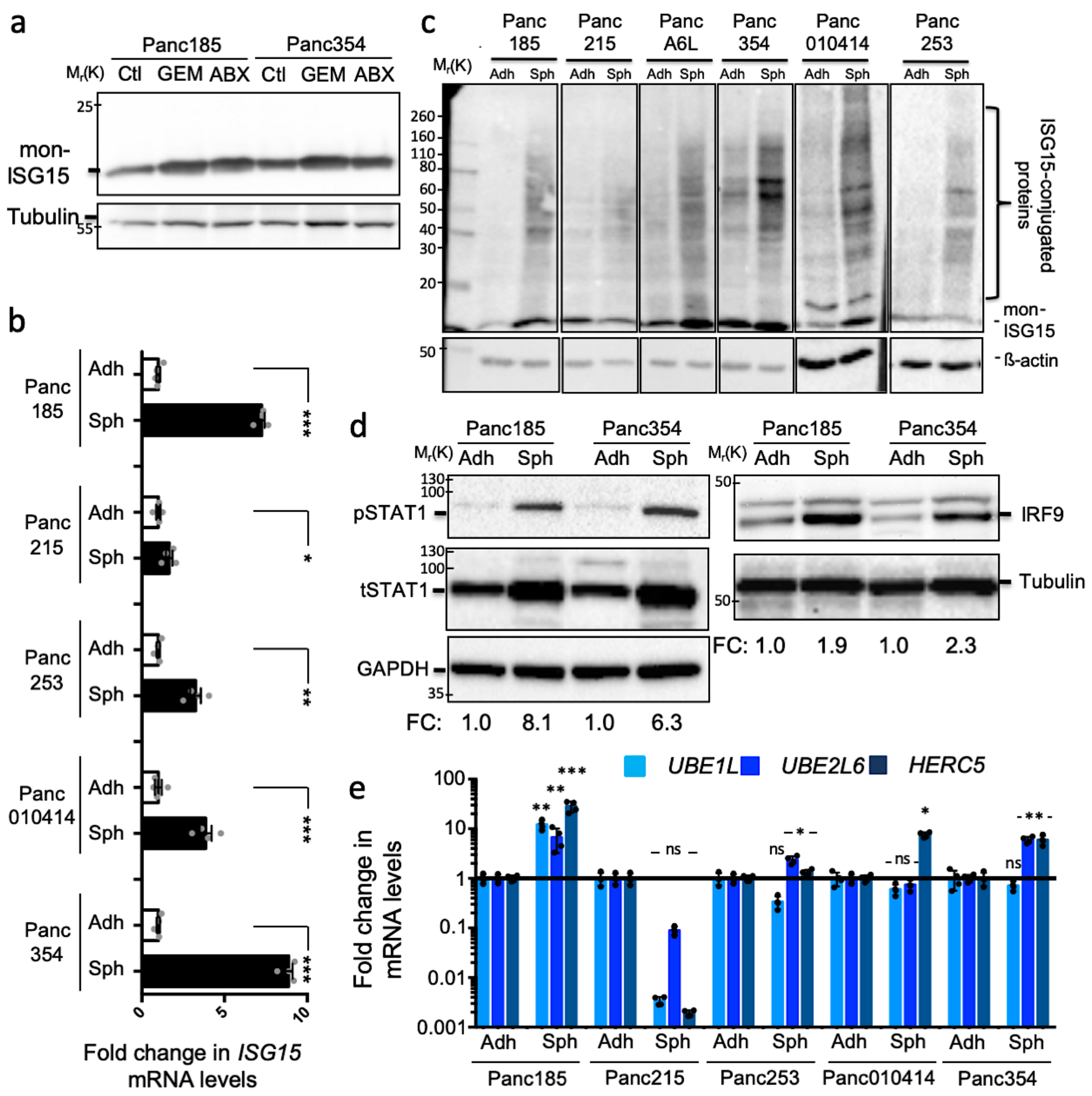

Supplementary Figure 1. ISG15 and ISGylation are enriched in PaCSCs. **a** WB analysis of mon-ISG15 from 2 different PDAC primary PDX cultures untreated (Ctl) or treated with Gemcitabine (GEM) or Abraxane (ABX). Tubulin was used as loading control. **b** Mean fold change in *ISG15* mRNA levels ± sd, determined by RTqPCR analysis, in 5 different PDAC primary PDX cells cultured as spheres (sph) or adherent (adh) (n=4 biologically independent samples; *p = 0.0322; **p = 0.011; ***p <0.001, Student's t-test). Adh set as 1.0. **c** WB analysis of monISG15 and ISG15-conjugated proteins in 6 different PDAC primary PDX cells cultured as adh or sph (CSCs and non-CSCs). β-Actin was used as loading control. **d** WB analysis of phosphoSTAT1 (pSTAT1), total STAT1 (tSTAT1) and IRF9 in 2 different PDAC primary PDX cells cultured as adh or sph (CSCs vs non-CSCs). GAPDH and Tubulin were used as loading controls. Densitometry calculations for pSTAT1/tSTAT1 or IRF9, normalized to GAPDH and Tubulin loading controls, respectively, are displayed as fold-change (FC) relative to adh, set as 1.0. **e** Mean fold change ± sd in *UBE1L*, *UBE2L6* and *HERC5* mRNA levels for 5 different PDAC primary PDX cells cultured as adh or sph (CSCs vs non-CSCs) (n=4 biologically independent samples; Student's t-test). Adh set as 1.0. *p < 0.05; **p < 0.01; ***p <0.001. ns, not significant.

a **GSE32688**

Name	ISG15	Ajcc stage	grade	tumor content
human PDAC 24 (mRNA)	6.840948002	2B	2	35
human PDAC 4 (mRNA)	8.373240463	2B	2	80
human PDAC 14 (mRNA)	8.804124817	2A	2	60
human PDAC 16 (mRNA)	9.340107457	2B	2	50
human PDAC 18 (mRNA)	9.467226147	2B	3	50
human PDAC 11 (mRNA)	9.477224133	2B	2	60
human PDAC 20 (mRNA)	9.542828999	2B	2	40
human PDAC 6 (mRNA)	9.684329646	2B	3	80
human PDAC 9 (mRNA)	9.702548619	2B	3	75
human PDAC 21 (mRNA)	9.716605927	2B	2	40
human PDAC 8 (mRNA)	9.727921009	2B	3	75
human PDAC 15 (mRNA)	9.751052064	2A	3	50
human PDAC 12 (mRNA)	9.854153083	2A	2	60
human PDAC 10 (mRNA)	9.9835262	2B	2	70
human PDAC 3 (mRNA)	10.03087305	2A	3	80
human PDAC 19 (mRNA)	10.15056329	2A	2	45
human PDAC 5 (mRNA)	10.32393688	2B	2	80
human PDAC 23 (mRNA)	10.46083698	2B	2	35
human PDAC 22 (mRNA)	10.68614311	2B	3	35
human PDAC 17 (mRNA)	10.69031661	2B	2	50
human PDAC 7 (mRNA)	11.32618711	1B	2	75
human PDAC 25 (mRNA)	11.35583477	2A	2	35
human PDAC 2 (mRNA)	11.428807	1B	2	90
human PDAC 13 (mRNA)	11.5505585	2B	3	60
human PDAC 1 (mRNA)	11.71256166	2B	2	90

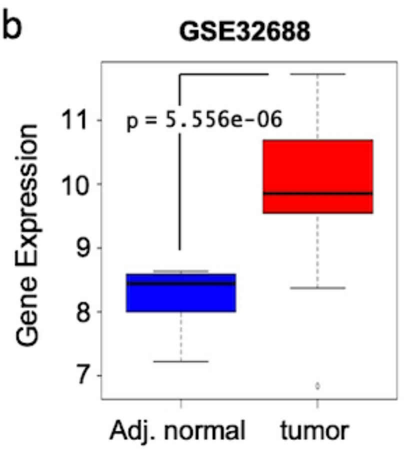

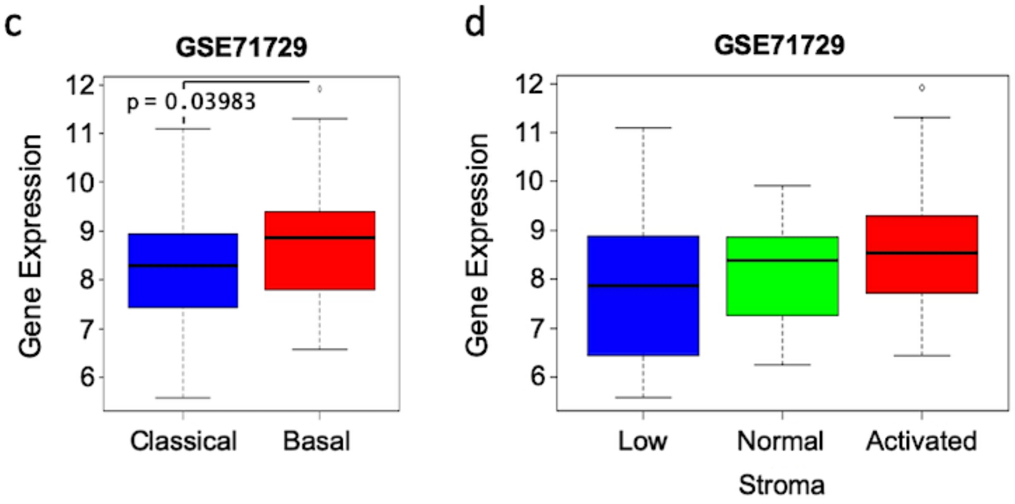

Supplementary Figure 2. ISG15 expression in epithelial cells and within tumor subtypes. a ISG15 mRNA levels in 25 different PDAC samples from GSE32688 dataset and their corresponding AJCC stage, grade and tumor content. **b** Box and Whisker Plots showing the differential expression of *ISG15* in normal adjacent (Adj.) tissue versus PDAC tumors in the GSE32688 dataset (7 Adj. normal, 25 tumors). **c-d** Box and Whisker Plots showing the differential expression of *ISG15* in tumors subtyped as Classical versus Basal **c** or classified as containing Low, Normal or Activated stroma **d** in the Moffitt *et al.* GSE71729 dataset. For box plots, rectangles show the first quartile, the median, and the third quartile. The two whiskers indicate the minimum and maximum values, and outliers are depicted as circles (unpaired two-sided Student's t-test).

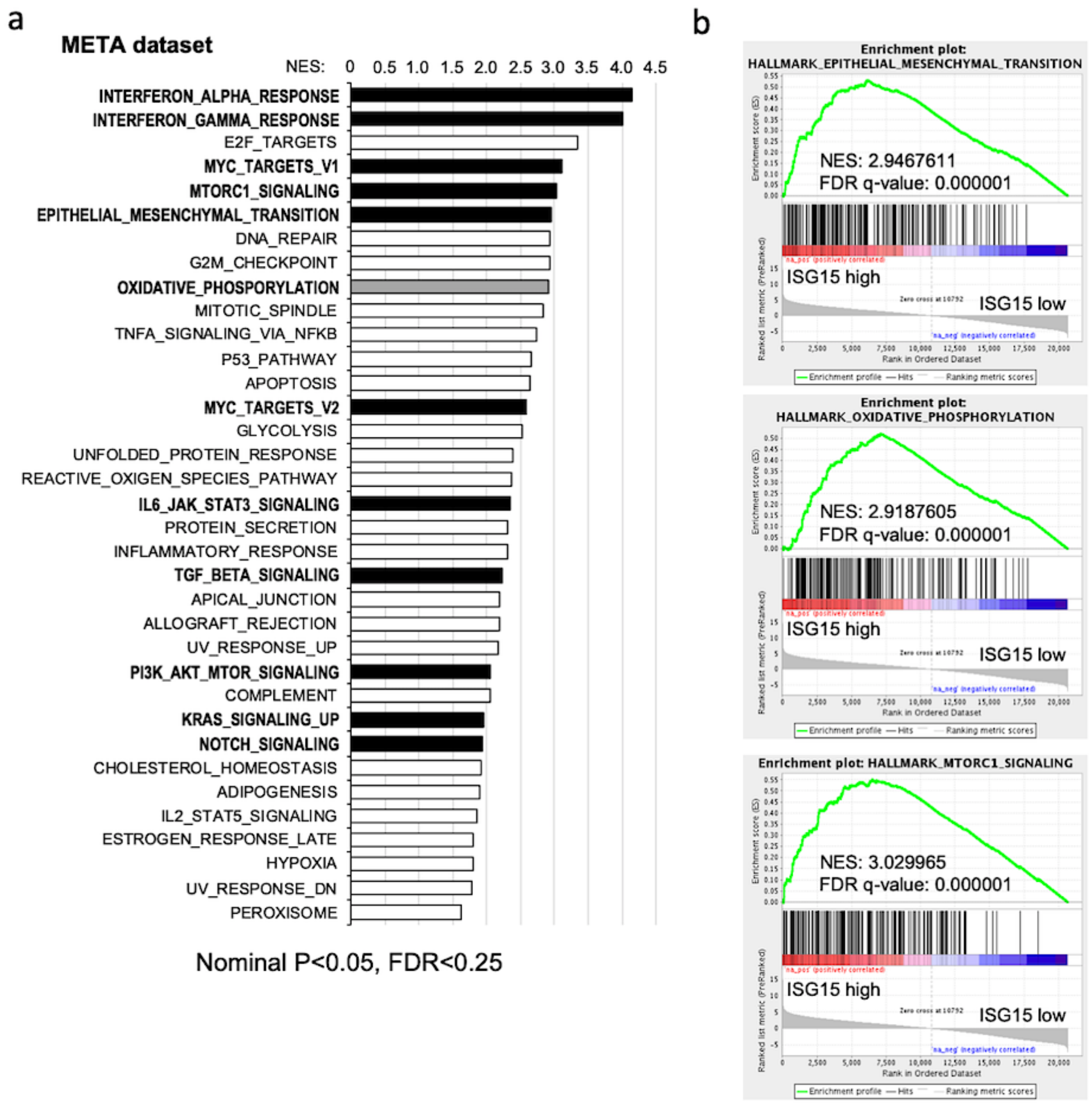

Supplementary Figure 3. Pathways enriched in ISG15 high PDAC tumors. a Gene sets enriched in the transcriptional profile of tumors belonging to the top *ISG15* high expression group, compared with the bottom expression group in the META dataset series. Shown are the NES (normalized enrichment score) values for each pathway using the Hallmark genesets, meeting the significance criteria: nominal p value of <0.05, FDR<25%. **b** Representative enrichment plots for EMT, OXPHOS and MTORC1 signaling.

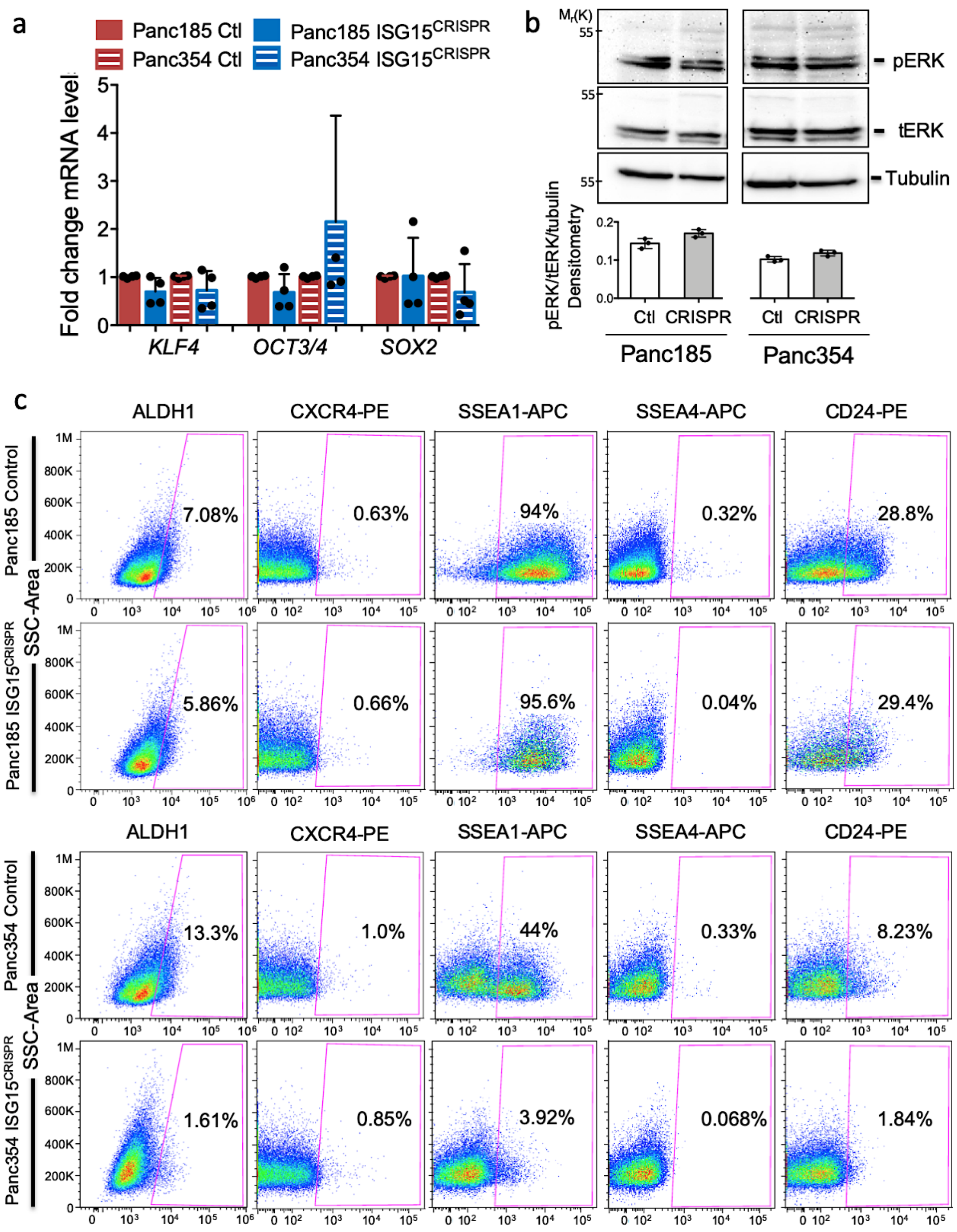

Supplementary Figure 4. Loss of ISG15/ISGylation does not affect PaCSC molecular phenotypes. a Mean fold change ± sd in *KLF4*, *OCT3/4* and *SOX2* mRNA levels in PDAC PDX primary cells comparing control and ISG15^{CRISPR} Panc185 and Panc354 cells. Data was normalized to β-actin expression. (n=4 biologically independent samples). Ctl set as 1.0. **b** WB analysis of phospho-ERK1/2 (pERK) and total ERK1/2 (tERK) protein expression in control and ISG15^{CRISPR} Panc185 and Panc354 cells (upper) and mean densitometry calculations normalized to Tubulin loading control ± sd (n=3 biologically independent blots) (lower). **c** Representative flow cytometry plots of ALDH-1, CXCR4, SSEA1, SSEA4 and CD24 expression in control and ISG15^{CRISPR} Panc185 and Panc354 cells.

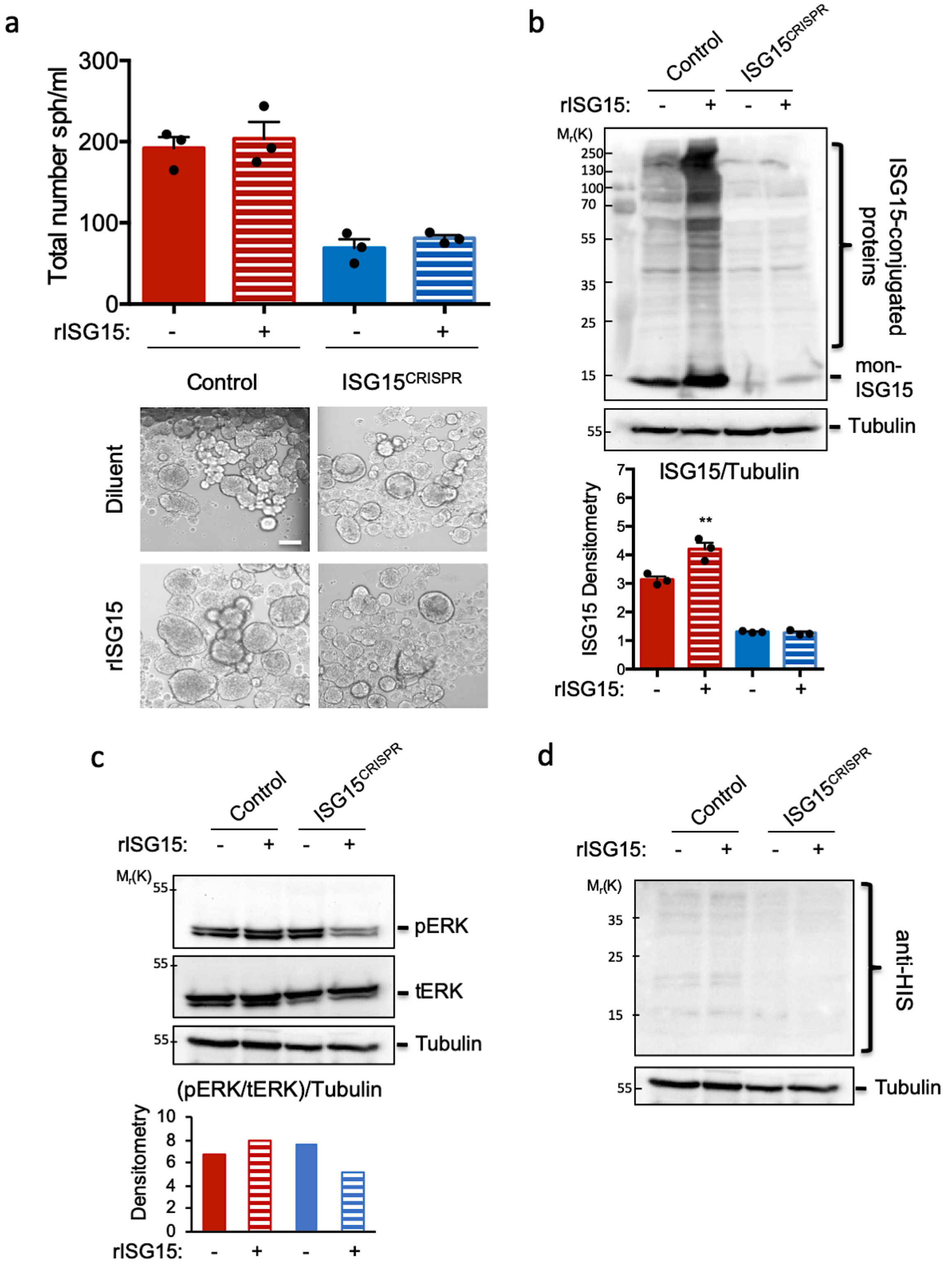

Supplementary Figure 5. rISG15 cannot rescue the self-renewal capacity of ISG15^{CRISPR} cells. a Sphere forming capacity (mean number of sph/ml) ± sd of Panc185 control and ISG15^{CRISPR} cells with or without rISG15-HIS (100ng/ml) treatment (n=3 biologically independent samples) (upper), and representative images of spheres formed 7 days post incubation (lower). Scale bar = 10µM. **b** WB analysis of monISG15 and ISG15-conjugated proteins (upper) and mean densitometric values ± sd normalized to Tubulin loading control (lower) (n=3 biologically independent samples; One-way ANOVA with Bonferroni's multiple comparisons test; **p = 0.0063). **c** WB analysis of phospho-ERK1/2 (pERK) and total ERK1/2 (tERK) (upper) and densitometry calculations normalized to Tubulin loading control (lower). **d** Analysis of Histidine (HIS) labeled proteins for the WB shown in **c**.

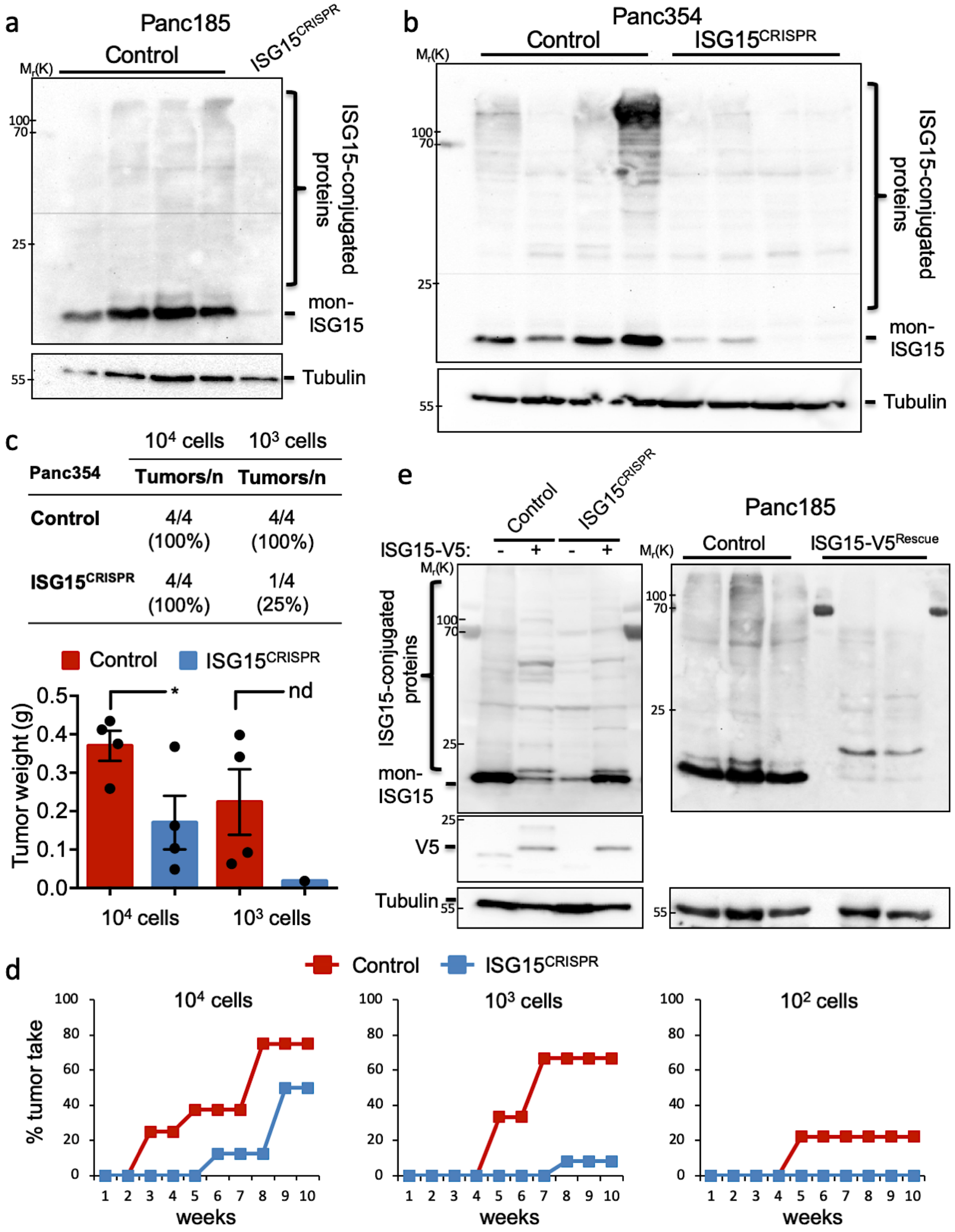

Supplementary Figure 6. Loss of ISG15/ISGylation affects tumorigenesis. a WB analysis of monISG15 and ISG15-conjugated proteins in freshly digested tumors obtained from tumors formed 10 weeks after injection of 10^3 Panc185 control and $ISG15^{CRISPR}$ cells *in vivo*. Tubulin was used as loading control. **b** WB analysis of monISG15 and ISG15-conjugated proteins in freshly digested tumors obtained from tumors formed 10 weeks after injection of 10^4 Panc354 control and $ISG15^{CRISPR}$ cells *in vivo*. Tubulin was used as loading control. **c** Quantification of tumors obtained from control and $ISG15^{CRISPR}$ cells, shown are the number of tumors/number of injections (n) and percentages (upper). Average tumor weights ± sem obtained in indicated groups (lower) (* = p= 0.0233, nd, not determined, Student's t-test). **d** Summary of in vivo tumor take and growth over 10 weeks for Panc185 control (ctl) and $ISG15^{CRISPR}$ cells injected at the indicated concentrations. **e** WB analysis of monISG15 and ISG15-conjugated proteins in Panc185 control and $ISG15^{CRISPR}$ cells uninfected or infected with the ISG15-V5 expressing lentivirus ($ISG15^{Rescue}$) prior to injection *in vivo* (left), and in tumors formed 10 weeks after injection of 10^4 Panc185 control or $ISG15^{CRISPR}$ cells infected with the ISG15-V5 expressing lentivirus ($ISG15\text{-}V5^{Rescue}$) *in vivo* (right). Detection of ISG15 in $ISG15\text{-}V5^{Rescue}$ cells was confirmed by hybridization with an anti-V5 antibody (middle). Tubulin was used as loading control (down).

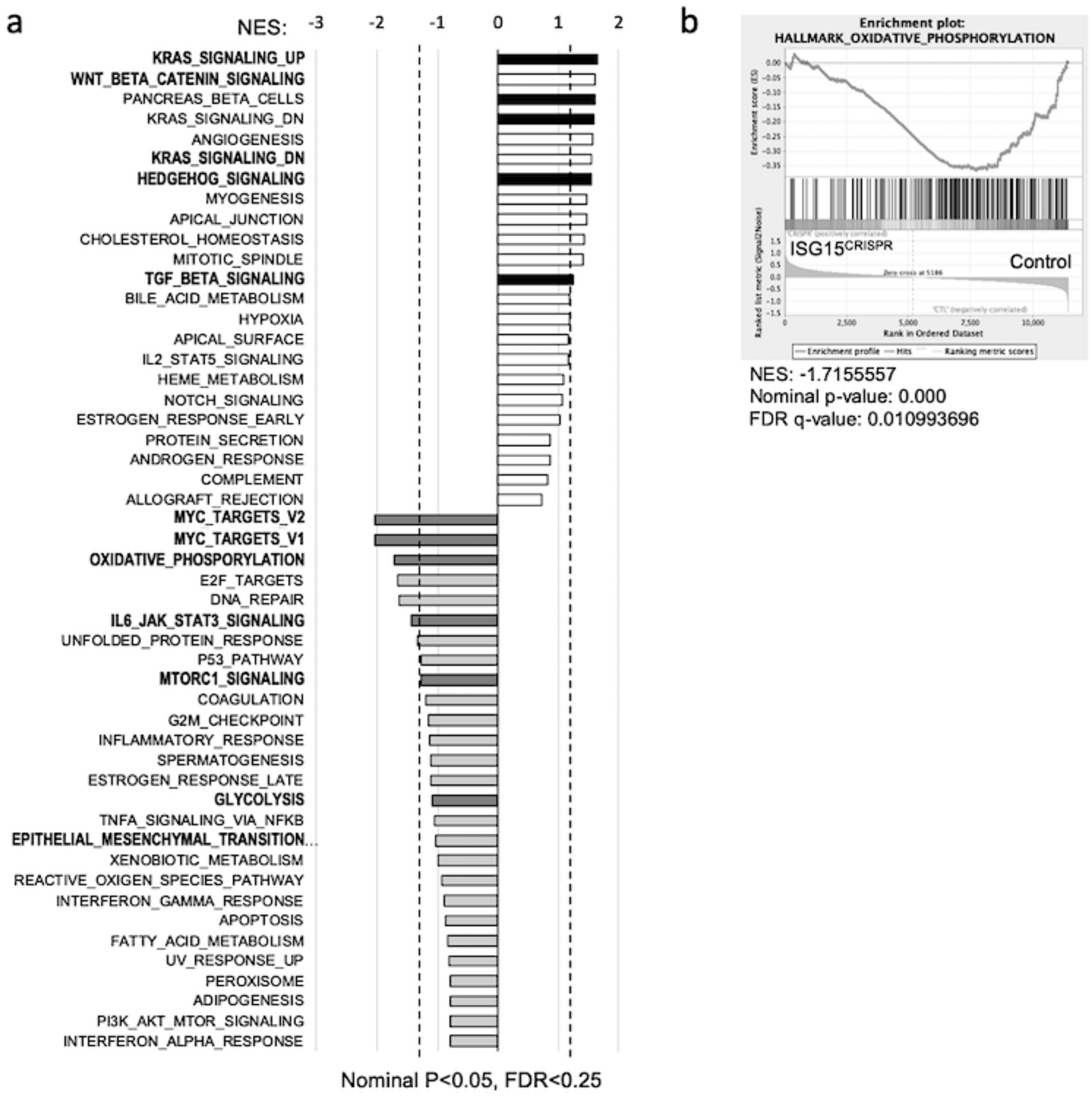

Supplementary Figure 7. Loss of ISG15 reduces OXPHOS signaling. a Gene sets enriched in the transcriptional profile of Panc185 control sphere-derived cells compared with ISG15^{CRISPR} sphere-derived cells. Dashed lines represent nominal p value of <0.05, FDR<25%, which is considered statistically significant. Shown are the NES (normalized enrichment score) values for each pathway using the Hallmark genesets. **b** Example enrichment plot for OXPHOS signaling.

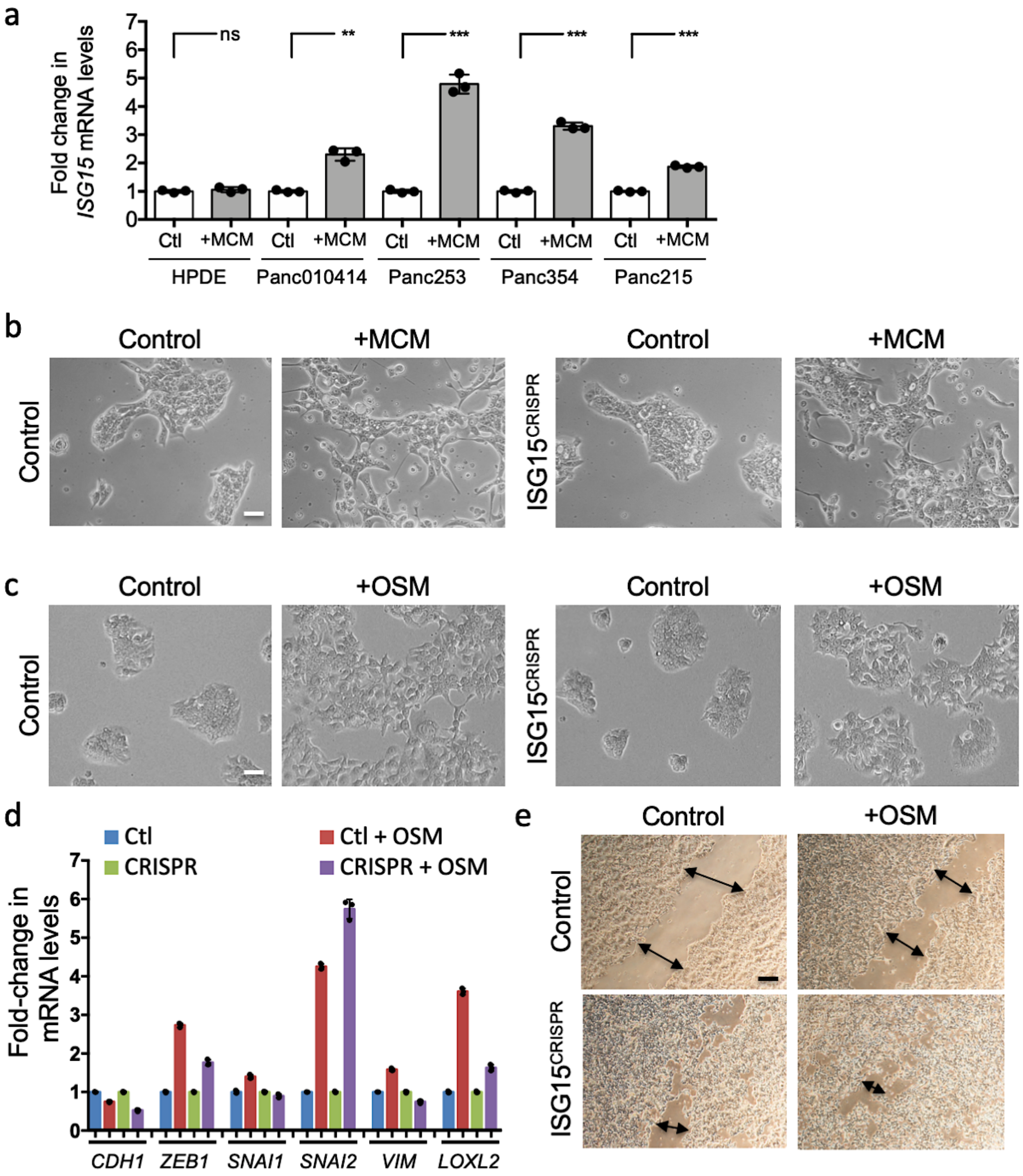

Supplementary Figure 8. Loss of ISG15/ISGylation does not affect EMT in PDAC cells. a Mean fold change ± sd in *ISG15* mRNA levels in 4 different PDAC primary PDX cultures and HPDE cells treated or untreated for 48 h with macrophage conditioned medium (MCM). (n=3 biologically independent samples; Student's t-test, **p = 0.005; ***p <0.001; ns, not significant). **b-c** Representative light micrographs of Panc354 control and ISG15^{CRISPR} cells untreated or treated with MCM **b** or 100 ng/ml of OSM **c**. Scale bar = 10µM. **d** Mean fold change in mRNA levels ± sd of indicated EMT-associated genes in Panc354 control and ISG15^{CRISPR} cells untreated or treated with 100 ng/ml of OSM for 48 h. (n=3 biologically independent samples). **e** Representative light micrographs of wound closure in Panc354 control and ISG15^{CRISPR} cells untreated and treated with 100 ng/ml of OSM for 48 h. Scale bar = 10µM.

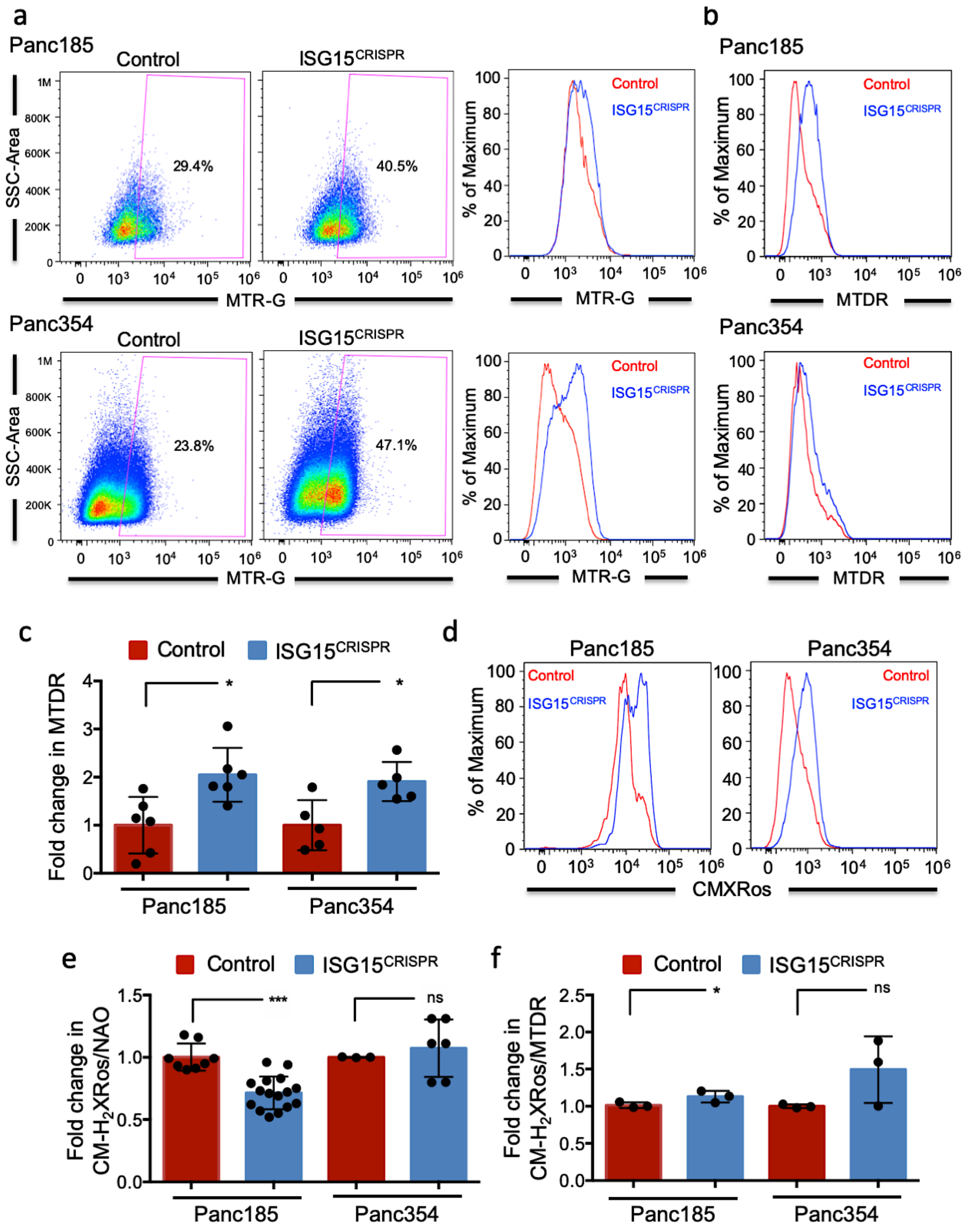

Supplementary Figure 9. Effect of ISG15 loss on mitochondrial mass and membrane potential. a Representative flow cytometric plots (left) and histograms (right) of Mitotracker Green (MTR-G) in control and ISG15^{CRISPR} Panc185 and Panc354 cells. **b-c** Representative histograms of flow cytometric analysis of Mitotracker Deep Red (MTDR) in control and ISG15^{CRISPR} Panc185 and Panc354 cells **b**, and mean fold changes ± sd in MTDR **c**, (Panc185: n=6 biologically independent samples; *p=0.0101; and Panc354: n=5 biologically independent samples; *p=0.0155; Student's t-test). **d** Representative histograms of flow cytometric analysis of CMXRos in control and ISG15^{CRISPR} Panc185 and Panc354 cells. **e-f** Mean fold changes in CM-H2XRos/NAO ratios ± sd (Panc185: n=8 or 16 biologically independent samples; ***p<0.001 Student's t-test; and Panc354: n=3 or 6 biologically independent samples; ns, not significant Student's t-test) **e** or CM-H2XRos/MTDR ratios ± sd (n=3 biologically independent samples; *p = 0.0417; ns, not significant Student's t-test) **f**.

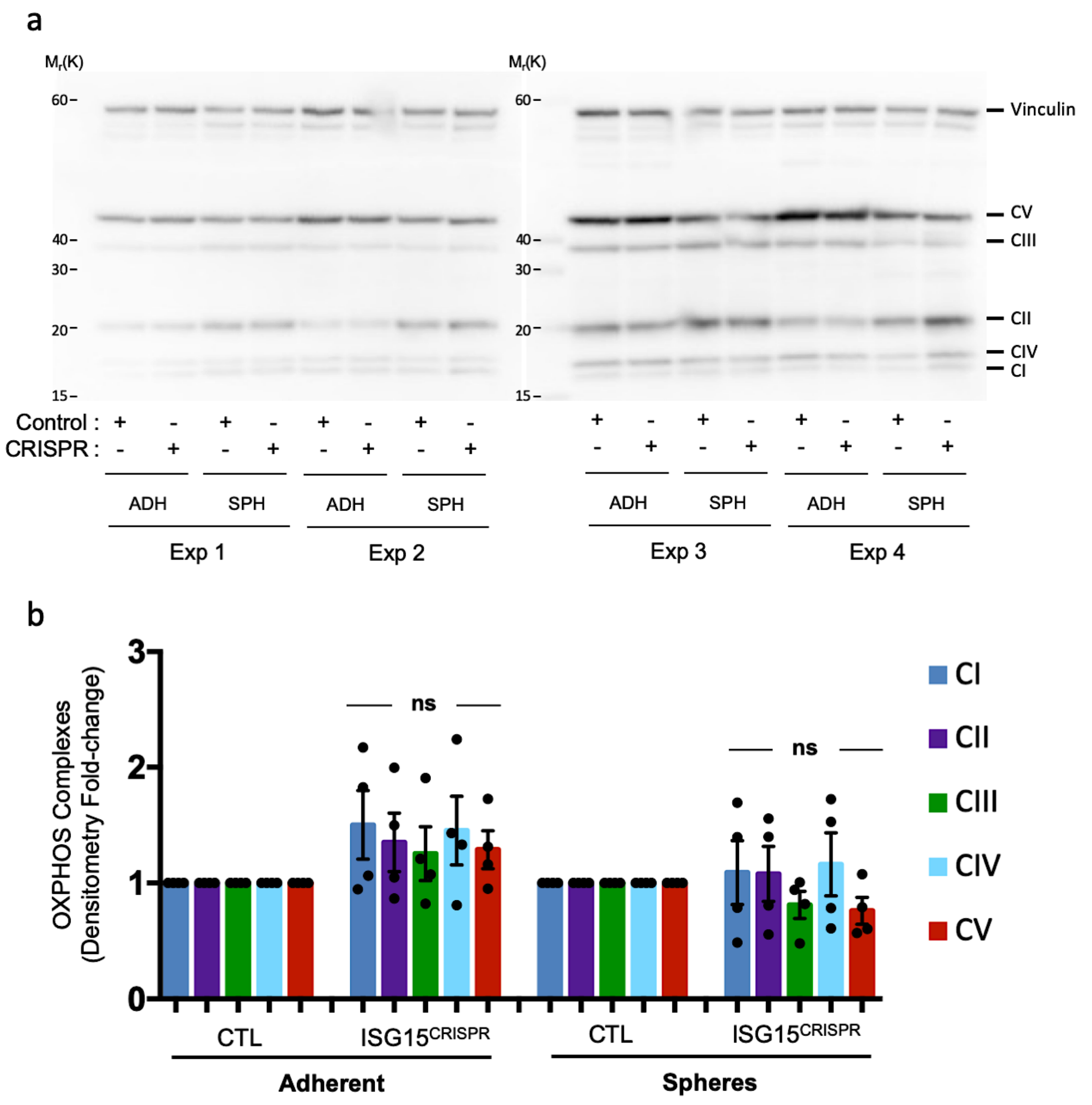

Supplementary Figure 10. Effect of ISG15 loss on OXPHOS complexes. a WB analysis of OXPHOS complexes determined using Total OXPHOS Human WB Antibody Cocktail in Panc185 control and ISG15^{CRISPR} cells cultured as adherent (non-CSCs) or spheres (CSCs) from 4 independent experiments. **b** Mean fold changes ± sd in densitometric analyses from **a** (n=4 biologically independent blots). Ctl set as 1.0. ns, not significant, determined with Student's t-test.

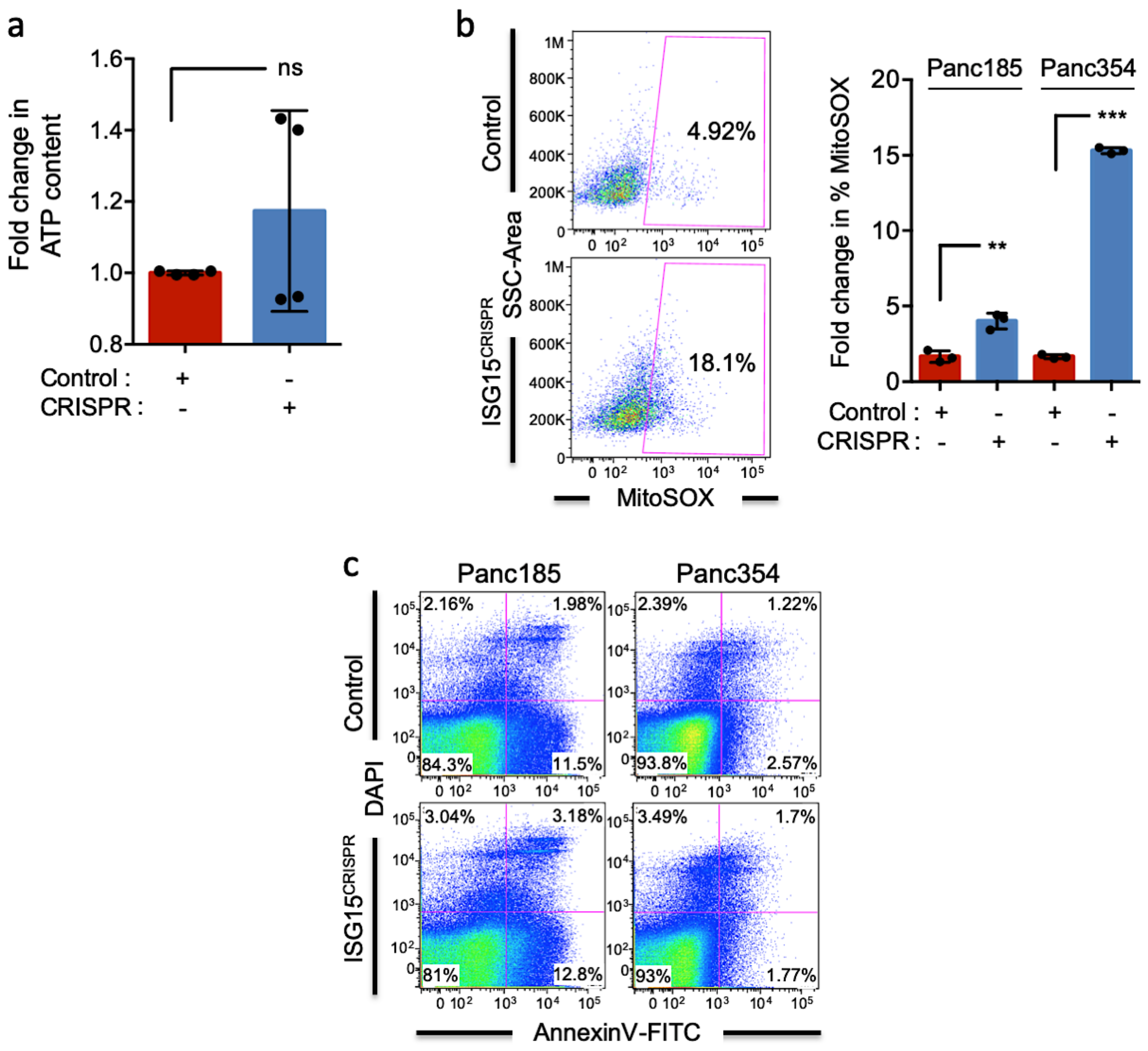

Supplementary Figure 11. Effect of ISG15/ISGylation loss on ATP content and ROS. a Mean fold change ± sd in ATP levels in Panc185 control and ISG15^{CRISPR} sphere-derived cells. (n=4 biologically independent samples; ns, not significant, Student's t-test). **b** Representative flow cytometric analysis of MitoSOX in Panc185 control and ISG15^{CRISPR} cells (left), and mean fold change ± sd in % MitoSOX levels (right) (n=3 biologically independent samples; **p = 0.0016; ***p <0.001 Student's t-test). **c** Representative flow cytometric analysis of AnnexinV and DAPI staining in control and ISG15^{CRISPR} Panc185 or Panc354 cells.

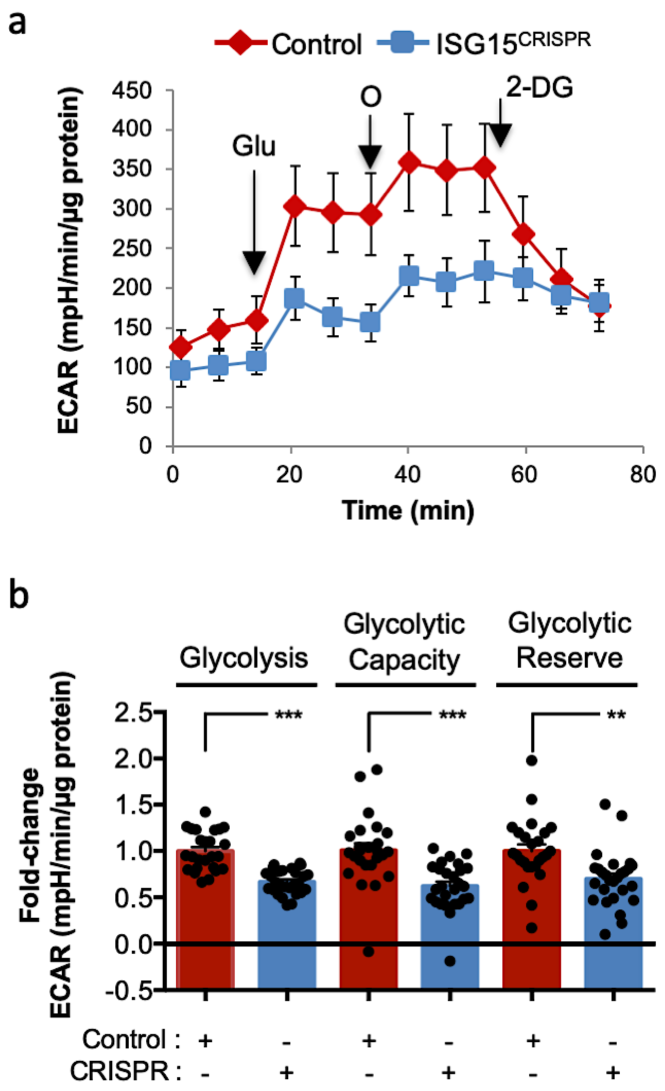

Supplementary Figure 12. Loss of ISG15/ISGylation impacts glycolytic reprogramming. a Extracellular acidification rate (ECAR) measurements ± sem in Panc185 control and ISG15^{CRISPR} cells subjected to sequential injections of glucose (Glu), Oligomycin (O) and 2-deoxyglucose (2DG). **b** Mean fold change ± sem in glycolysis after Glu injection, glycolytic capacity after Glu and O sequential injection and glycolytic reserve after Glu, O and 2-DG sequential injection in control and ISG15^{CRISPR} cells. (6 measurements per time point examined over 5 independent experiments; $**p = 0.0017$; $***p < 0.001$ Student's t-test).

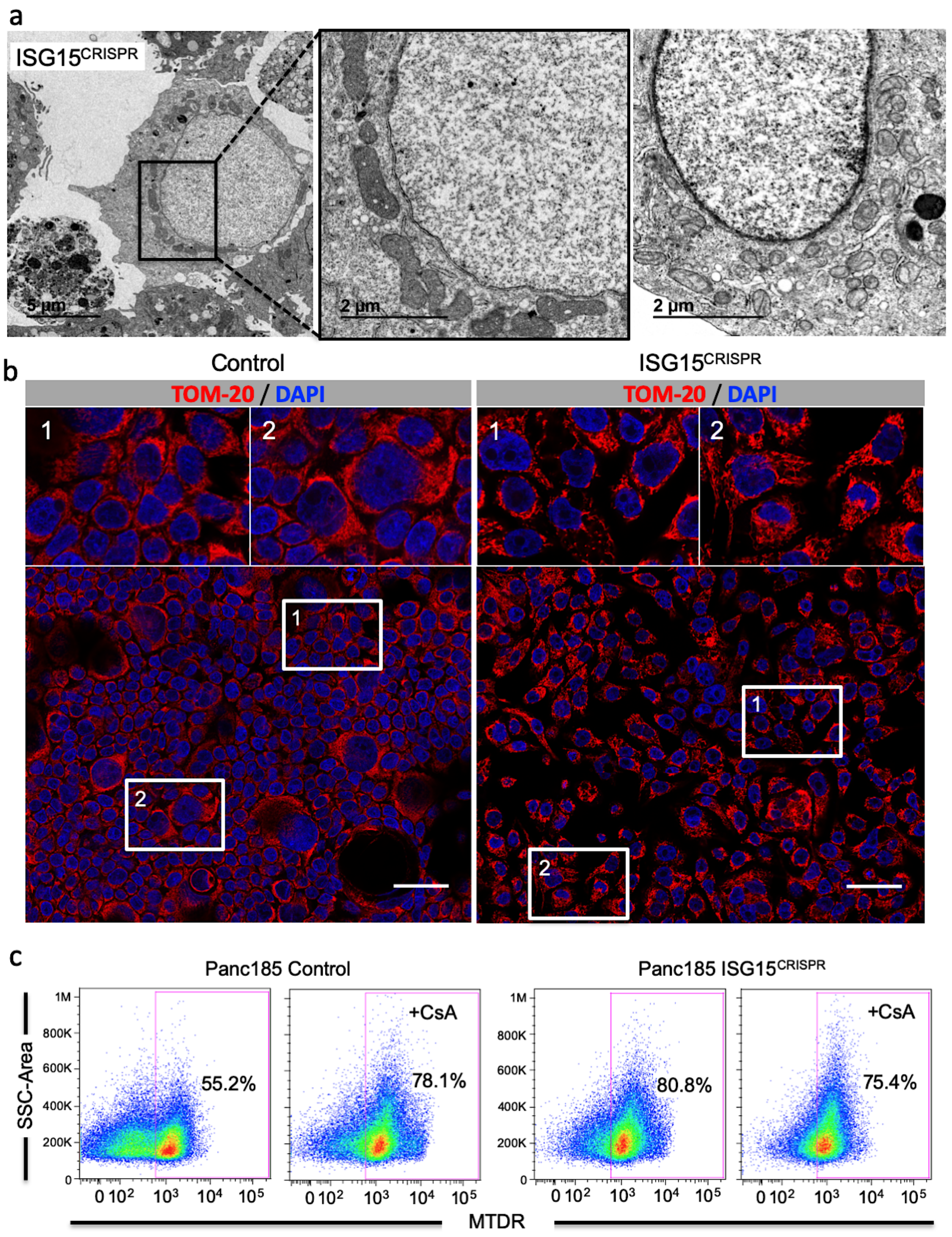

Supplementary Figure 13. Loss of ISG15/ISGylation promotes mitochondrial fission and impairs mitophagy. **a** Transmission electron micrographs of Panc354 ISG15^{CRISPR} cells. Scale bars = 5 or 2 µM. **b** Representative IF confocal micrographs of TOM-20 in control and ISG15^{CRISPR} Panc354 cells. Nuclei stained with DAPI in blue. Zoomed insets are numbered and outlined in white. Scale bar = 20 µm. **c** Representative flow cytometry analysis of Mitotracker Deep Red (MTDR) in Panc185 control and ISG15^{CRISPR} untreated or treated with CsA (5µM) for 5 h.

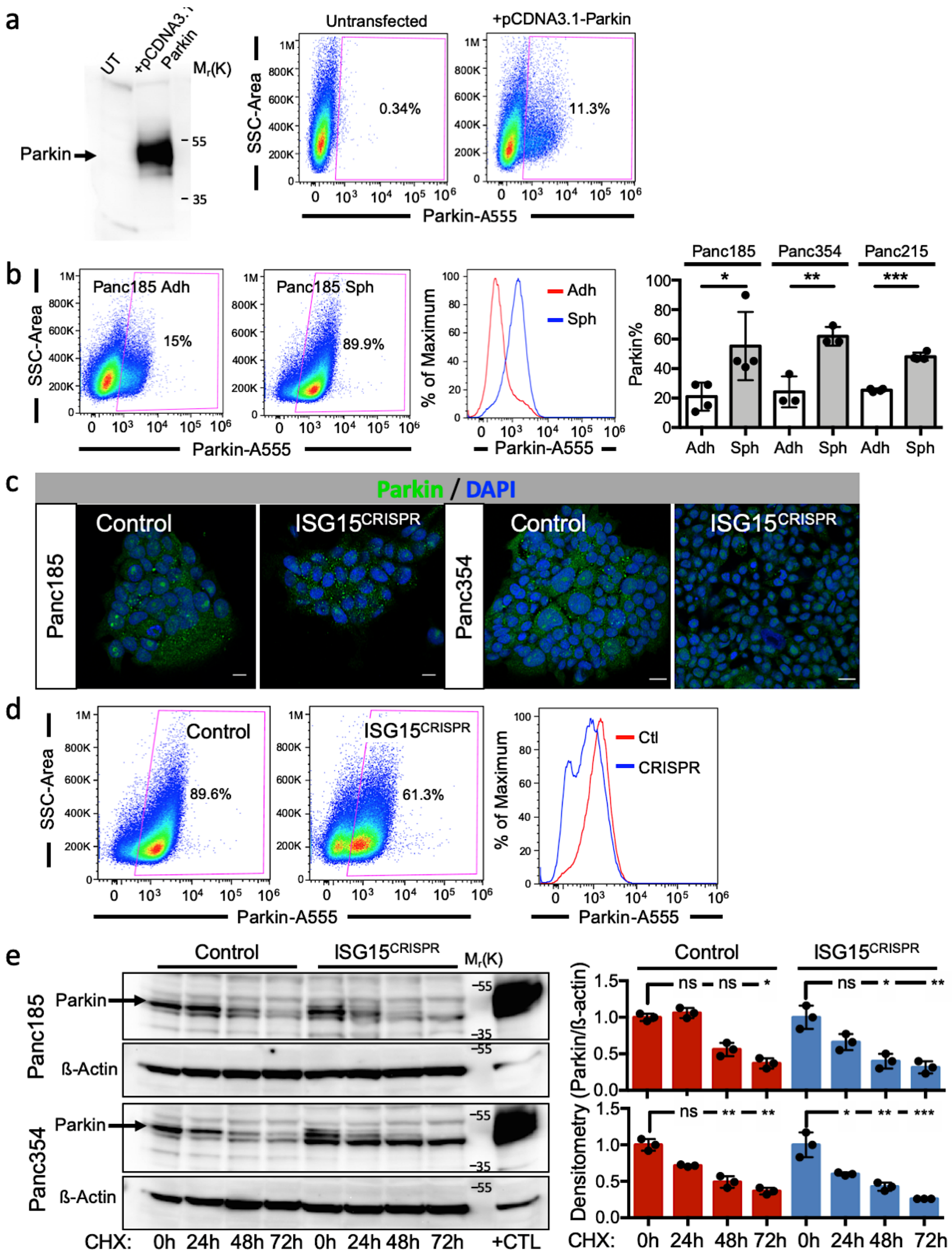

Supplementary Figure 14. Parkin is enriched in PaCSCs and its stability is regulated by ISG15. a WB (left) and flow cytometric analysis (right) of 293T cells un-transfected or transiently transfected with pCDNA3.1-Parkin. **b** Representative flow cytometric dot blots (left) and histogram analysis (middle) of intracellular parkin levels in Panc185 adherent (non-CSCs) and spheres (CSCs), and summary of mean intracellular parkin levels ± sd in adherent (non-CSCs) and spheres (CSCs) in three PDX PDAC cultures (right) (Panc185: n=4 biologically independent samples *p = 0.0337; Panc354: n=3 biologically independent samples **p= 0.0059; and Panc215: n=3 biologically independent samples ***p<0.001; Student's t-test). **c** Representative IF confocal micrographs of parkin and DAPI in control and ISG15^{CRISPR} Panc185 and Panc354 cells. Scale bar = 20 µm. **d** Flow cytometric dot blots (left) and histogram analysis (right) of intracellular parkin levels in Panc354 control and ISG15^{CRISPR} sphere-derived cultures. **e** WB analysis of Parkin in control and ISG15^{CRISPR} Panc185 and Panc354 cells treated with CHX for indicated times. ßactin was used as loading control (left). Mean fold changes ± sd in Parkin/ßactin densitometric levels (n=3 biologically independent blots; *p < 0.05; **p < 0.01; ***p <0.001; ns, not significant as determined by One-way ANOVA with Bonferroni's multiple comparisons test). 0h set as 1.0.

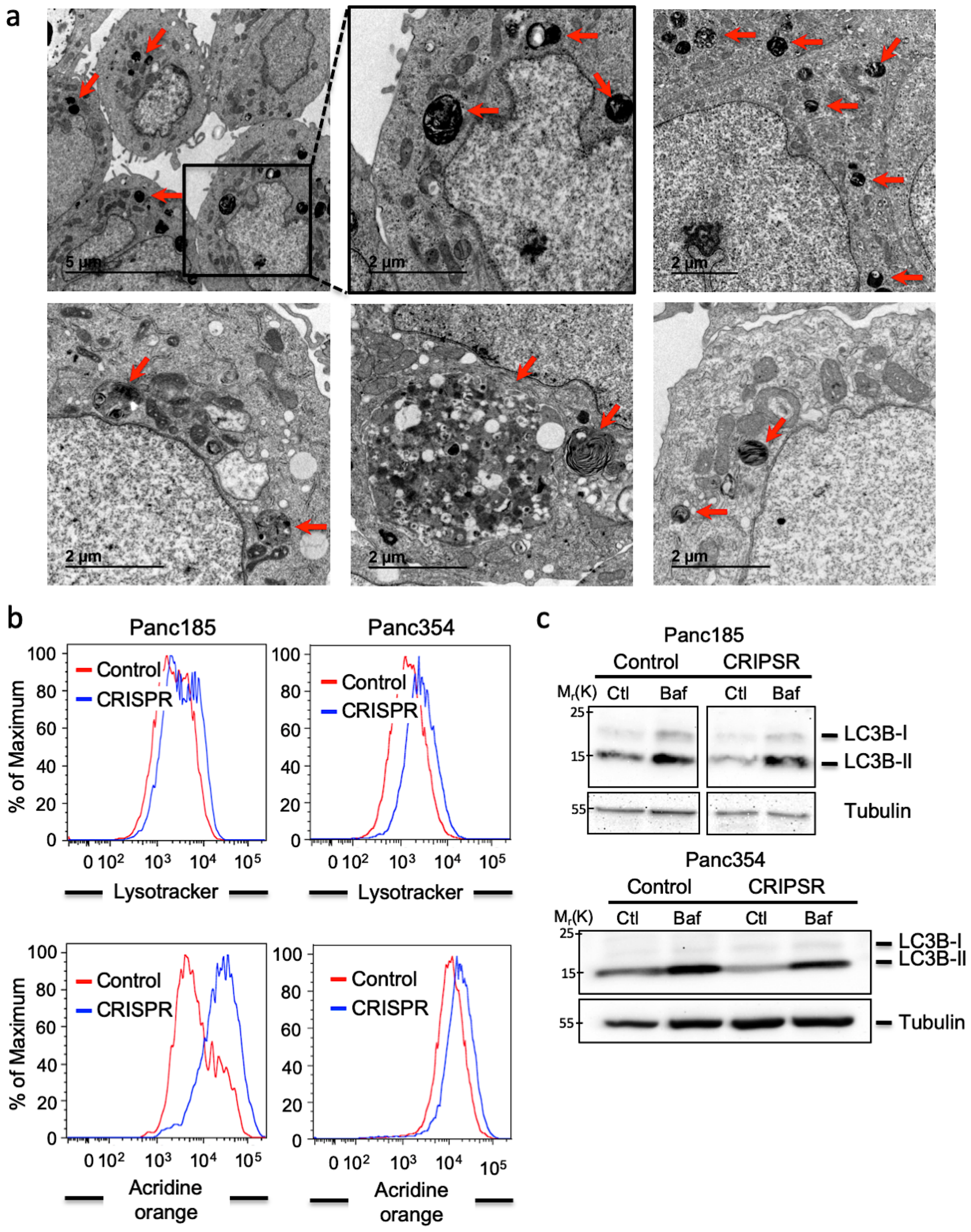

Supplementary Figure 15. Increased autophagosomes and lysosomes in the absence of ISG15/ISGylation. a Transmission electron micrographs of Panc354 ISG15^{CRISPR} cells. Red arrows indicate autophagosomes. Scale bars = 5 and 2 µM. **b** Representative flow cytometric analysis of Lysotracker and Acridine Orange in control and ISG15^{CRISPR} Panc185 and Panc354 cells. (**C**) Additional WB analyses of LC3B in control and ISG15^{CRISPR} Panc185 and Panc354 cultures untreated (control, Ctl) or treated with the autophagy inhibitor Bafilomycin (Baf) for 5 h. Tubulin was used as loading control.

Figure 1C

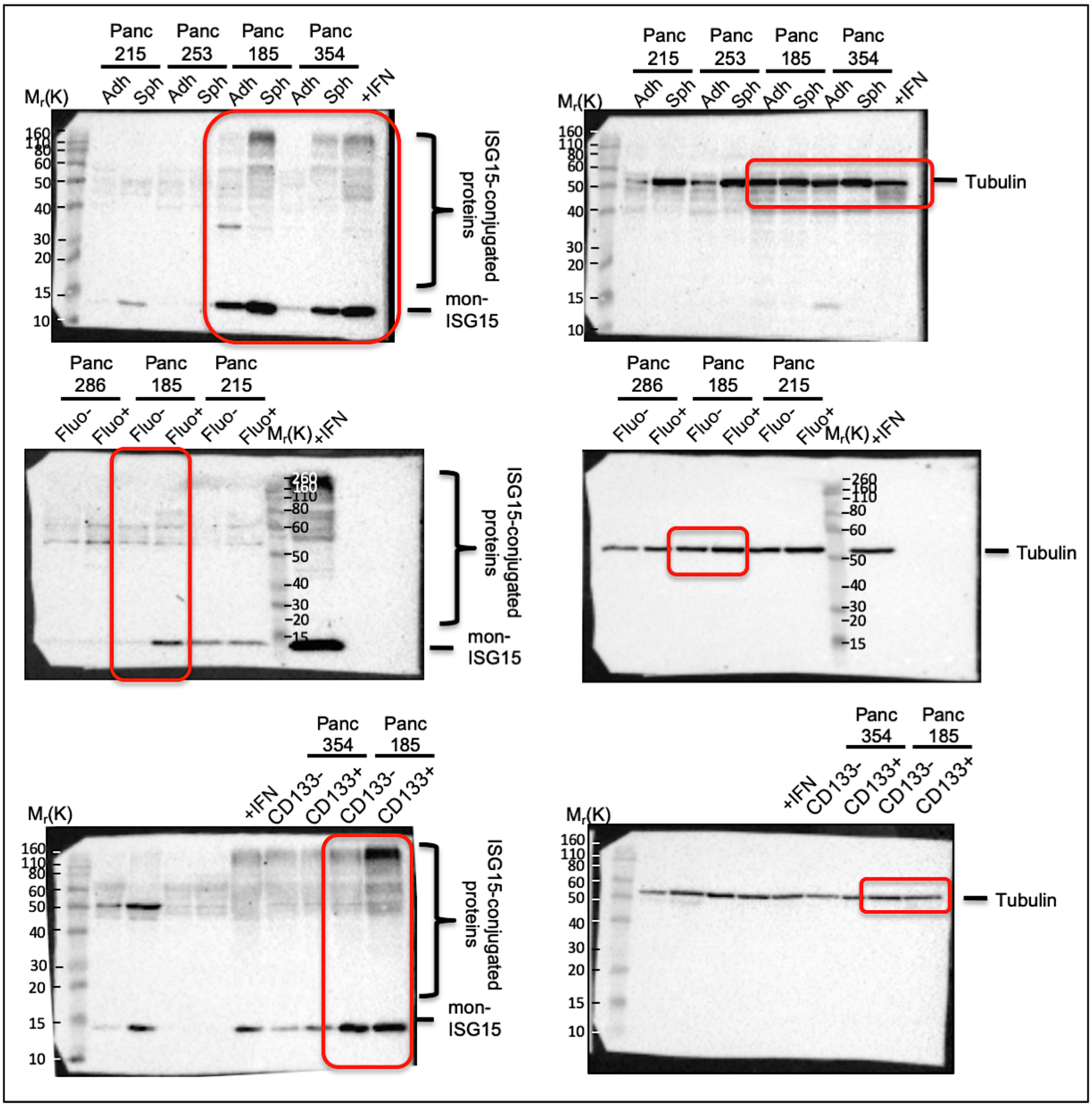

Supplementary Figure 16. Unprocessed scans of immunoblots

Figure 2D

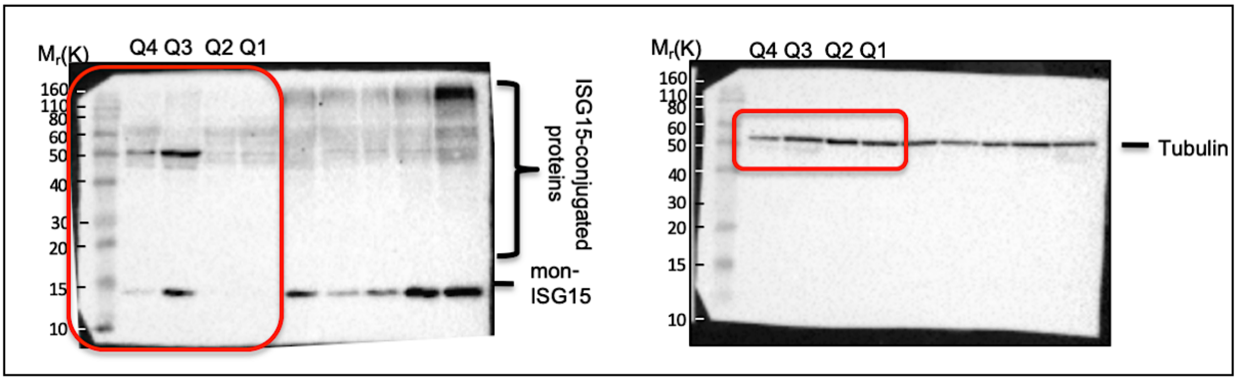

Figure 2F

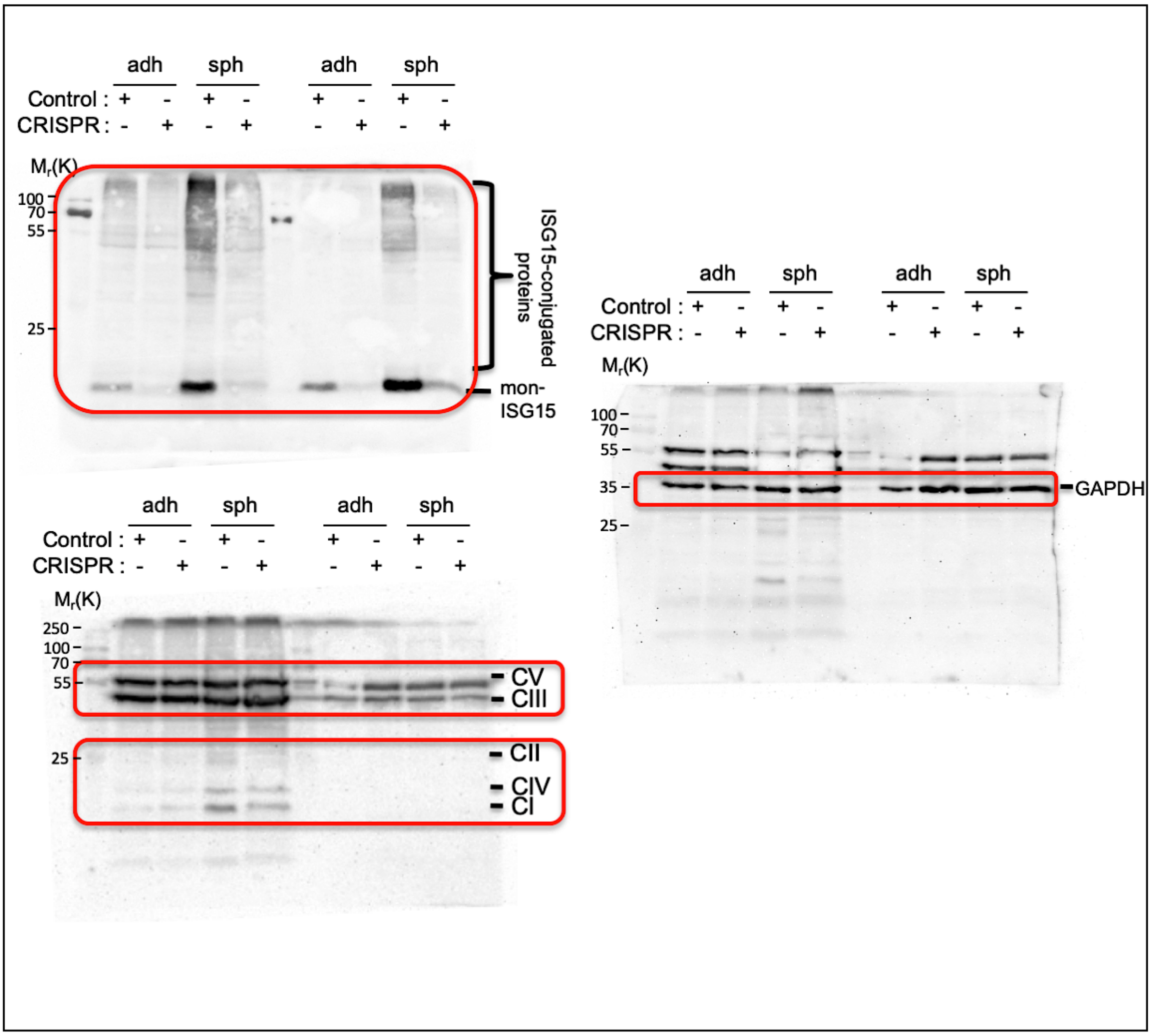

Supplementary Figure 16. continued

Figure 3A

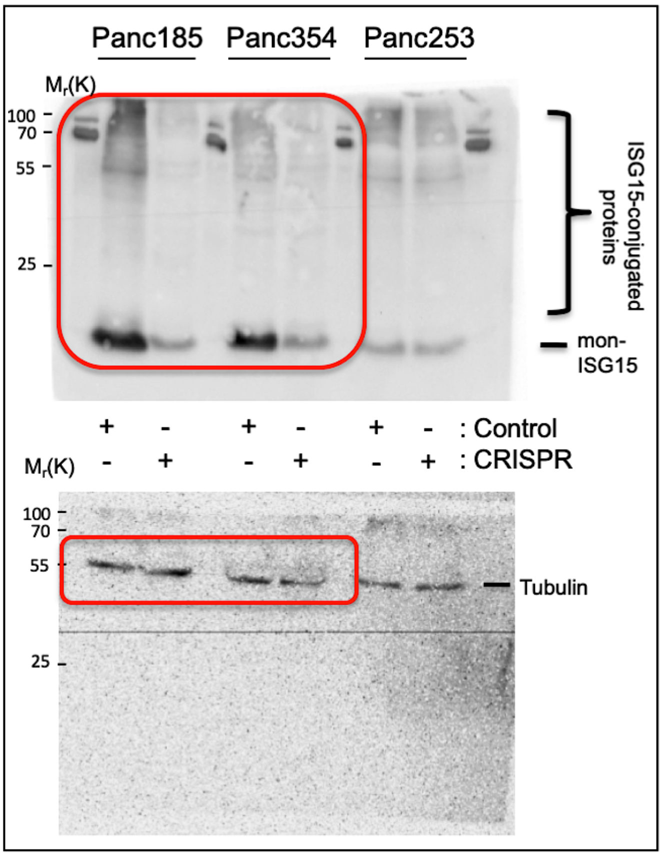

Figure 3G

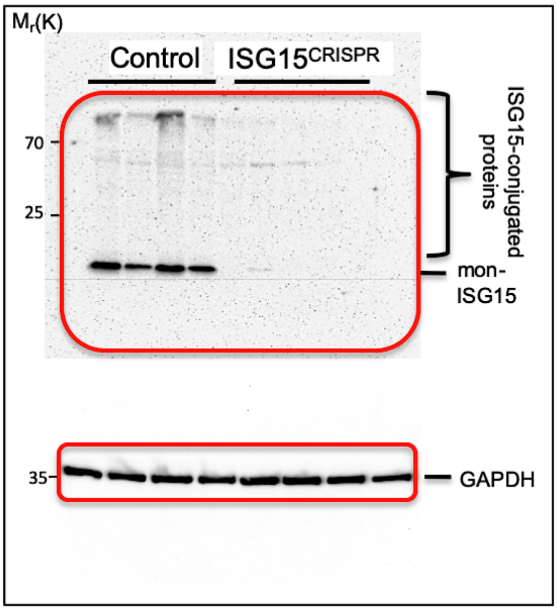

Figure 6C

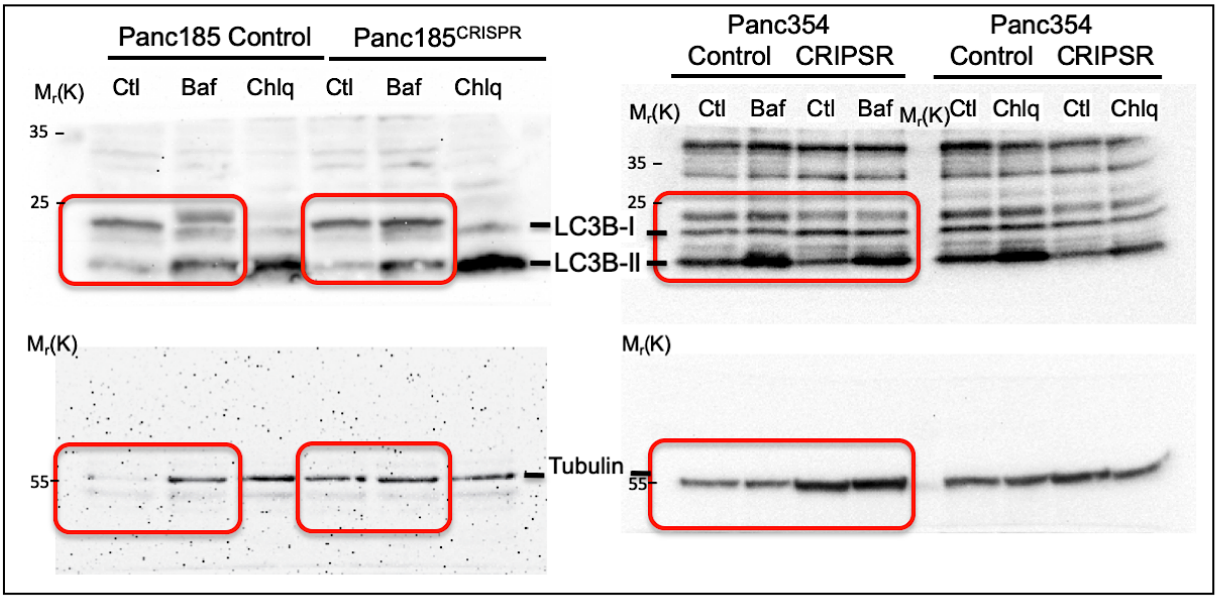

Supplementary Figure 16. continued

Supplementary Figure 1D

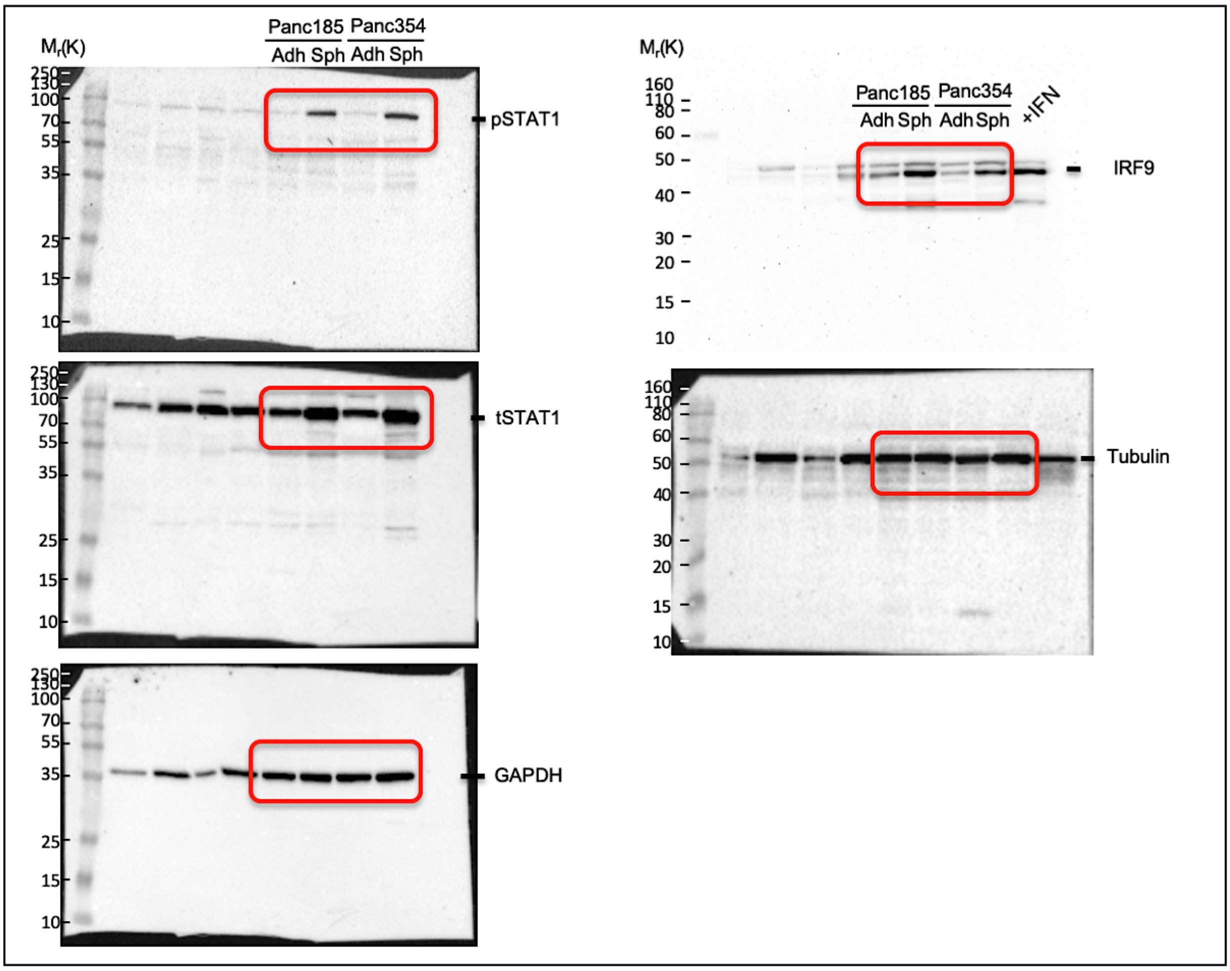

Supplementary Figure 4B

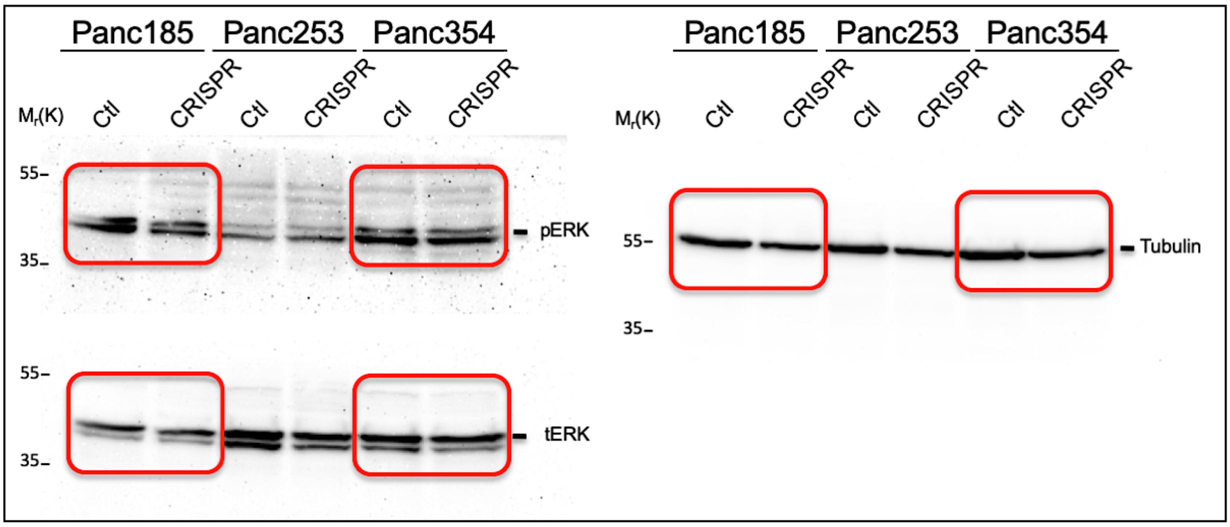

Supplementary Figure 16. continued

Supplementary Figure 5B

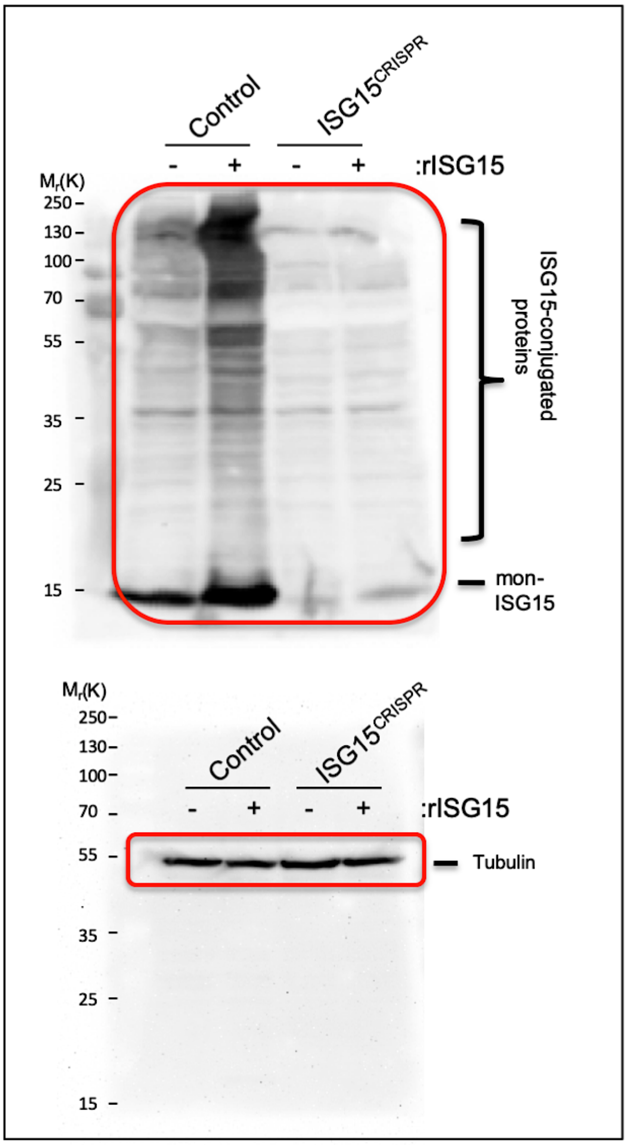

Supplementary Figure 5C

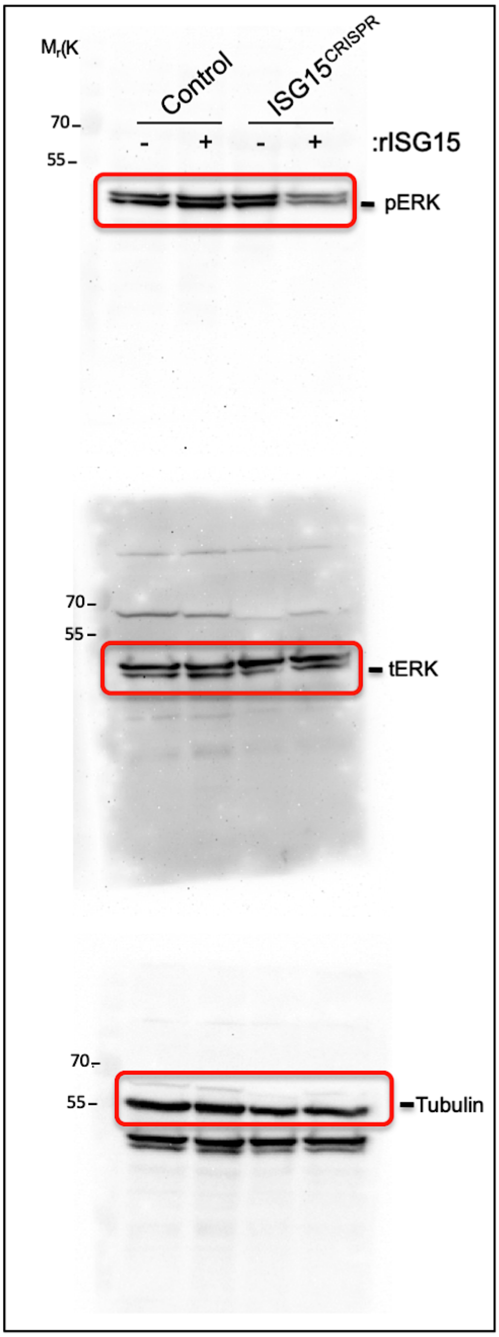

Supplementary Figure 16. continued

Supplementary Figure 6E

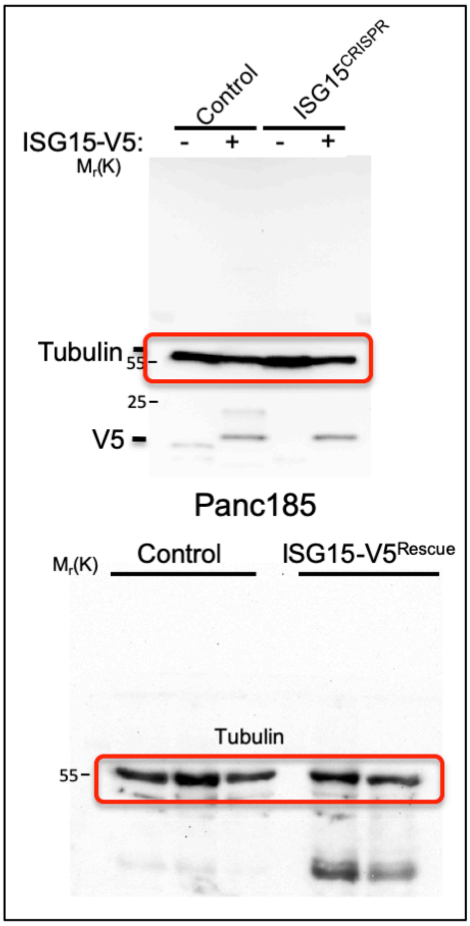

Supplementary Figure 14E

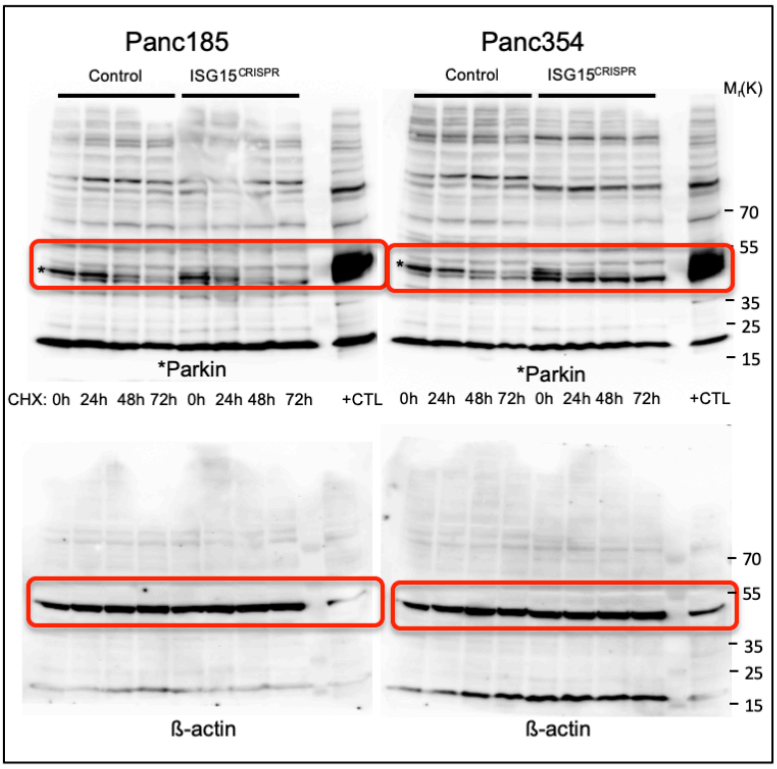

Supplementary Figure 15C

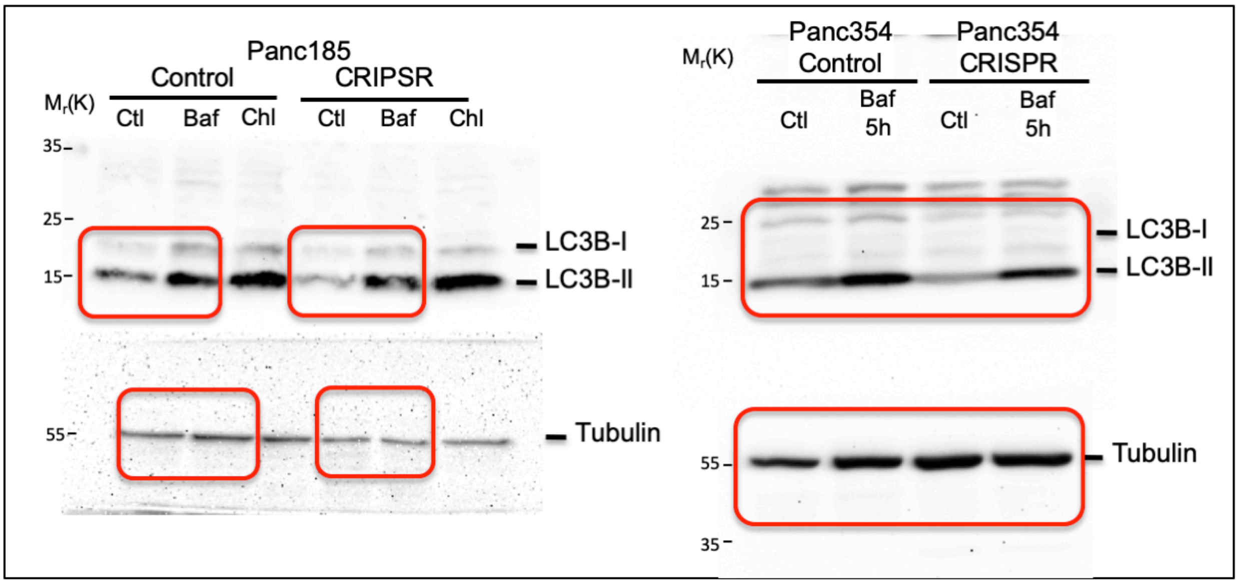

Supplementary Figure 16. continued

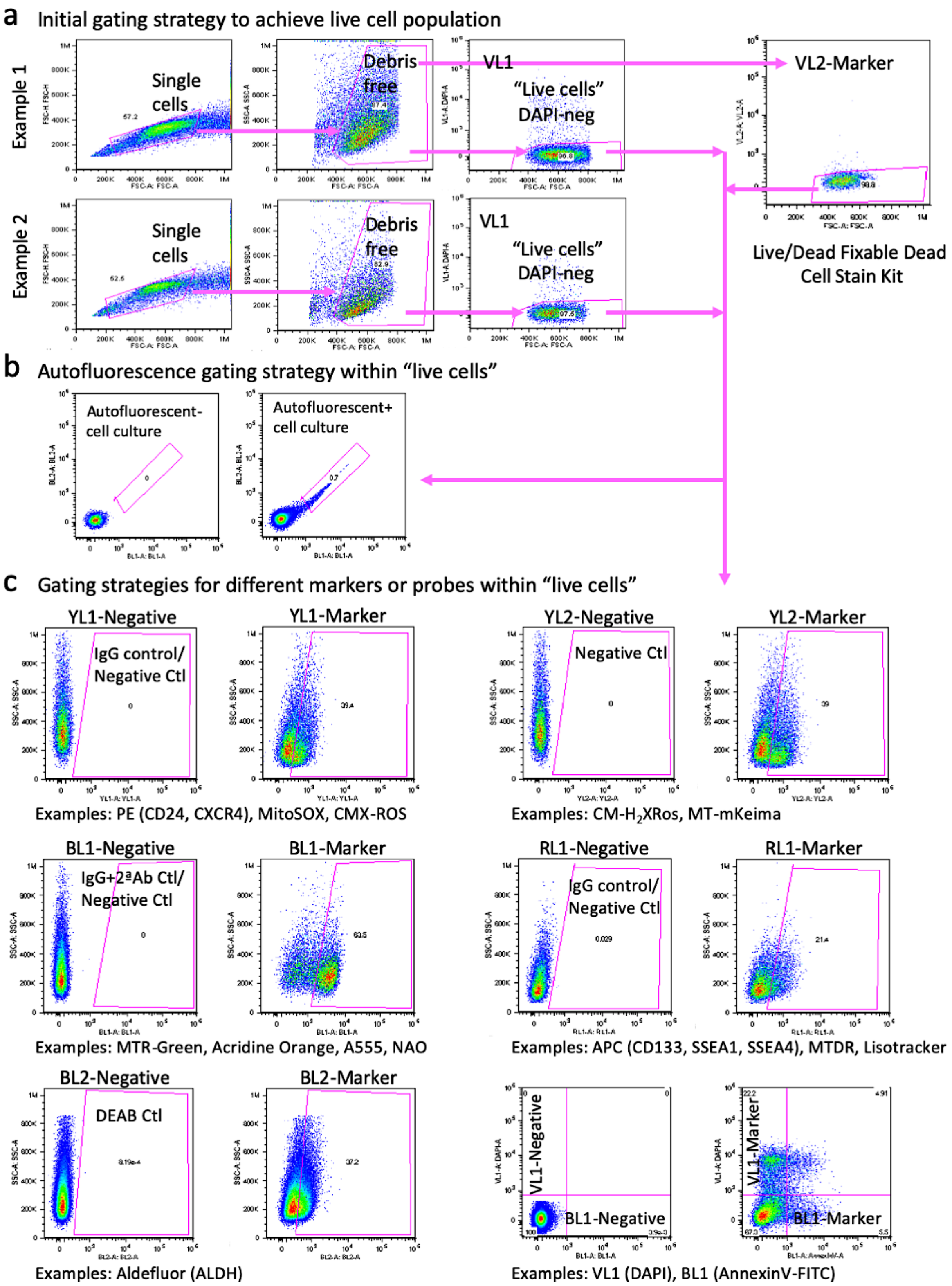

Supplementary Figure 17. Flow cytometry gating strategies. a Two examples showing initial gating strategies to acquire a single cell, debris free and DAPI-free ("live cell") population, applied to all analyses. Or an example using the Live/Dead Fixable Dead Cell Stain Kit (right). **b** Gating strategy for the detection of autofluorescent cells within the "live cell" population. **c** Gating strategy for the detection of specific antibody-labeled or probe-labeled cells within the "live cell" population. Shown are the IgG, negative or unstained controls, the corresponding gates and a positive staining example with indicated filter(s) and laser(s). Indicated below are the specific markers or probes for which the indicated gating strategy was applied. Additional details can be found in the Methods section.
