# Supplementary material for: ISG15 and ISGylation is required for pancreatic cancer stem cell mitophagy and metabolic plasticity
Source: Nat Commun. 2020 May 29;11:2682. doi: 10.1038/s41467-020-16395-2 (PMC7260233; doi:10.1038/s41467-020-16395-2)
Supplement: Supplementary file 2 — Reporting Summary [file 41467_2020_16395_MOESM2_ESM.pdf]

nature research

Corresponding author(s): Bruno Sainz, Jr.

Last updated by author(s): Apr 16, 2020

Reporting Summary

Nature Research wishes to improve the reproducibility of the work that we publish. This form provides structure for consistency and transparency in reporting. For further information on Nature Research policies, see Authors & Referees and the Editorial Policy Checklist.

Statistics

For all statistical analyses, confirm that the following items are present in the figure legend, table legend, main text, or Methods section.

n/a	Confirmed	
☐	☒	The exact sample size (*n*) for each experimental group/condition, given as a discrete number and unit of measurement
☐	☒	A statement on whether measurements were taken from distinct samples or whether the same sample was measured repeatedly
☐	☒	The statistical test(s) used AND whether they are one- or two-sided<br>*Only common tests should be described solely by name; describe more complex techniques in the Methods section.*
☒	☐	A description of all covariates tested
☒	☐	A description of any assumptions or corrections, such as tests of normality and adjustment for multiple comparisons
☐	☒	A full description of the statistical parameters including central tendency (e.g. means) or other basic estimates (e.g. regression coefficient) AND variation (e.g. standard deviation) or associated estimates of uncertainty (e.g. confidence intervals)
☐	☒	For null hypothesis testing, the test statistic (e.g. *F*, *t*, *r*) with confidence intervals, effect sizes, degrees of freedom and *P* value noted<br>*Give P values as exact values whenever suitable.*
☒	☐	For Bayesian analysis, information on the choice of priors and Markov chain Monte Carlo settings
☒	☐	For hierarchical and complex designs, identification of the appropriate level for tests and full reporting of outcomes
☒	☐	Estimates of effect sizes (e.g. Cohen's *d*, Pearson's *r*), indicating how they were calculated

Our web collection on statistics for biologists contains articles on many of the points above.

Software and code

Policy information about availability of computer code

Data collection	Invitrogen™ Attune™ NxT software, version 3.1.1 was used for cytometry data collection, unless specified<br>For side population cytometry data collection, CytExpert Version 2.3 was used (Beckman Coulter)<br>XF96 1.4.2 Software (Agilent) for Seahorse data collection
Data analysis	For image preparation and analysis: Fiji package of ImageJ V2.0.0-rc-64/1.51s and Adobe Photoshop CS6<br>For flow cytometry image preparation and analysis: FlowJo 9.3 software (Tree Star Inc., Ashland, OR.)<br>For pathway enrichment plots and NES graphs: GSEA version 3.0, Broad Institute<br>For graphs preparation and analysis: Prism 6.0 for Mac, GraphPad Software, Inc<br>To generate Box and Whisker plots and Kaplan–Meier survival plots: R Studio version 1.1.442 and R version 3.5.1 (RStudio, Boston, MA)<br>For OCR and ECAR analsyses: XF96 1.4.2 Software (Agilent)<br>Complete analysis of RNA-seq data was done with the pipeline: Nextpresso 1.9.2.2 (CNIO, Madrid, Spain).

For manuscripts utilizing custom algorithms or software that are central to the research but not yet described in published literature, software must be made available to editors/reviewers. We strongly encourage code deposition in a community repository (e.g. GitHub). See the Nature Research guidelines for submitting code & software for further information.

Data

Policy information about availability of data

All manuscripts must include a data availability statement. This statement should provide the following information, where applicable:

- Accession codes, unique identifiers, or web links for publicly available datasets
- A list of figures that have associated raw data
- A description of any restrictions on data availability

RNAseq data from Control and ISG15CRISPR Panc185 cells, generated in this study, have been deposited in the ArrayExpress database (ref 58) at EMBL-EBI (www.ebi.ac.uk/arrayexpress) under accession number E-MTAB-8984. Unique identifiers for the publicly available datasets used are indicated, and source data

other than those provided in the Article or Supplementary Information are available from the corresponding author upon reasonable request.

Field-specific reporting

Please select the one below that is the best fit for your research. If you are not sure, read the appropriate sections before making your selection.

☒ Life sciences ☐ Behavioural & social sciences ☐ Ecological, evolutionary & environmental sciences

For a reference copy of the document with all sections, see nature.com/documents/nr-reporting-summary-flat.pdf

Life sciences study design

All studies must disclose on these points even when the disclosure is negative.

Sample size	No statistical methods were used to predetermine the sample size. Sample sizes for experiments were estimated based on previous experience with a similar setup that showed significance. Published examples include (Sancho P, et al. MYC/PGC-1α Balance Determines the Metabolic Phenotype and Plasticity of Pancreatic Cancer Stem Cells. Cell Metab. 2015 Oct 6;22(4):590-605. PMID: 26365176; and Hermann PC et al. Multimodal Treatment Eliminates Cancer Stem Cells and Leads to Long-Term Survival in Primary Human Pancreatic Cancer Tissue Xenografts. PLoS One. 2013 Jun 18;8(6):e66371. PMID: 23825539). Experiments involved mice were performed two to three times (as indicated) and 5-6 animals where analyzed for each experiments/ time point. Each study was designed to use the minimum number of mice required to obtain informative results (that is, quantitative data amenable to statistical analysis).
Data exclusions	No data were excluded
Replication	Experiments were repeated and experimental findings were reproducible. Specifically, we have included in the manuscript the following reproducibility statement "The number of biologically independent samples are indicated in the Figure legends. Repeated independent experiments per each panel with similar results are shown below. n=1 (Figs. 1f, 2d-f, 3b, 3d, 1h, 4a, 5a, 6a-b, 7a, 7c, Supplementary Figs. 1d-e, 4b, 5c-d, 6a-b, 6e, 13a, 15a); n=2 (Figs. 1b, 1c, 3e, 3g, 4b-e, 5f-g, 7d-e, Supplementary Figs. 1a-c, 5a, 6c-d, 8e, 10a-b, 11a-b, 14b, 14d, 15b); n= 3 (Figs. 3a, 5b-c, 5d-e, 6c-d, Supplementary Figs. 4a, 5b, 8a-d, 9a-f, 13b-c, 14a, 14c, 14e, 15c), n=5 (Figs. 4f-g, Supplementary Figs. 11c, 12a-b)."
Randomization	Mice were allocated randomly to each treatment group.
Blinding	No formal blinding was used. Tumor measurements were conducted by an independent technician who was unaware of the hypothesis.

Reporting for specific materials, systems and methods

We require information from authors about some types of materials, experimental systems and methods used in many studies. Here, indicate whether each material, system or method listed is relevant to your study. If you are not sure if a list item applies to your research, read the appropriate section before selecting a response.

Materials & experimental systems

n/a	Involved in the study	
☐	☒	Antibodies
☐	☒	Eukaryotic cell lines
☒	☐	Palaeontology
☐	☒	Animals and other organisms
☐	☒	Human research participants
☒	☐	Clinical data

Methods

n/a	Involved in the study	
☒	☐	ChIP-seq
☐	☒	Flow cytometry
☒	☐	MRI-based neuroimaging

Antibodies

Antibodies used

Primary Antibodies:
α-hu-CD133/1-APC, Mouse monoclonal, FACS 1:10, Miltenyi Biotec,(Cat no. 130-090-826)
α-hu-ISG15, Rabbit 1:500, WB ProteinTech (Cat no. 15981-1-AP)
α-beta-ACTIN, Mouse monoclonal, 1:5000 WB ThermoFisher (Cat no.MA1-140)
α-GAPDH, Mouse monoclonal, 1:5000 WB ThermoFisher (Cat no.MA5-15738)
α-hu-LC3BI/II, Rabbit 1:500/1:100 WB/IF Sigma (Cat no. L7543)
α-hu-LAMP1 (H43A), Mouse monoclonal 1:100 IF Santa Cruz (Cat no. sc-20011)
α-hu-PARKIN Rabbit, 1:500/1:200/100 WB/IF/FC ThermoFisher (Cat no.PA5-13398)
α-hu-phospho-ERK1/2, Rabbit 1:500 WB Cell Signalling (Cat no. 9101S)
α-hu-ERK1/2 Rabbit, 1:500 WB Cell Signalling (Cat no. 9102S)
α-TUBULIN, Mouse monoclonal 1:5000 WB Elabscience (E-AB-20033)
α-VINCULIN, Mouse monoclonal 1:1000 WB Sigma (SAB4200080)
α-hu-SSEA1-APC, Mouse monoclonal 1:10 FC Miltenyi Biotec (Cat no.130-104-990)
α-hu-SSEA4-APC, Mouse monoclonal 1:50 FC BioLegend (Cat no. 330418)

α-hu-CD24-PE, Mouse monoclonal 1:10 FC Miltenyi Biotec (Cat no.130-108-381)
α-V5 Mouse monoclonal, 1:1000 WB Invitrogen (Cat no. R960-25)
α-hu-phospho-STAT1, Rabbit 1:1000 WB Cell Signalling (Cat no. 9177)
α-hu-STAT1, Rabbit 1:1000 WB Cell Signalling (Cat no. 9172)
α-hu-IRF9, Mouse monoclonal 1:500 WB Santa Cruz (Cat no. sc-365893)
TOM20, Mouse monoclonal 1:200 IF Santa Cruz (Cat no. sc-17764)
α-HIS Tag, Mouse monoclonal 1:1000 WB ThermoFisher (Cat no. MA1-21315)
Total OXPHOS Human WB Antibody Cocktail Mouse monoclonal 1:500 WB Abcam (Cat no. ab110411)
Secondary Antibodies
α-mouse-HRP, Sheep 1:5,000 WB Amersham (Cat no. NA9310-1ML)
α-rabbit-HRP Donkey 1:5,000 WB Amersham (Cat no. NA9340-1ML)
α-mouse Alexa 647 Goat 1:500 IF Invitrogen (Cat. no. A32728)
α-rabbit Alexa 555 Goat 1:500 IF/FC Invitrogen (Cat no. A32732)

Validation

All antibodies were validated by the manufacturers. Please refer to the commercial website of each antibody for more details. For the anti-Parkin antibody, we validated its use for WB and Flow Cytometry using 293T transiently transfected with a plasmid (pCDN3.1-Parkin) expressing the full coding sequence of Parkin (NM_004562.3), kindly provided by Drs. Raúl Sánchez Lanzas and José González Castaño, Universidad Autónoma de Madrid, Madrid, Spain. These cells were used to determine the specificity of the antibody, as shown in Supplementary Figure 14A, comparing the signal in the transfected sample to the signal in the untransfected sample.

Eukaryotic cell lines

Policy information about cell lines

Cell line source(s)

HPDE cells were kindly provided by Dr. Francisco Real from the Spanish National Cancer Centre (CNIO)
HEK 293T cells were obtained from ATCC
PDAC patient-derived xenografts (PDAC PDX) were obtained from Dr. Manuel Hidalgo under a Material Transfer Agreement with the Spanish National Cancer Centre (CNIO), Madrid, Spain (Reference no. I409181220BSMH). PDX-derived cultures are referred to by a random number designation (e.g. Panc185, Panc253 or Panc354, etc).

Authentication

Cells were periodically authenticated by microsatellite analysis

Mycoplasma contamination

All cells were tested for mycoplasma every 4 weeks and were confirmed to be mycoplasma negative

Commonly misidentified lines
(See ICLAC register)

No commonly misidentified cell lines were used in the study.

Animals and other organisms

Policy information about studies involving animals; ARRIVE guidelines recommended for reporting animal research

Laboratory animals

Female 6- to 8-week-old NU-Foxn1nu nude mice (Envigo, Spain). Mice were housed according to the following guidelines: a 12 h light/12 h dark cycle, with no access during the dark cycle; temperatures of 65-75°F (~18-23°C) with 40-60% humidity; a standard diet with fat content ranging from 4% to 11; sterilized water was accessible at all times; for handling, mice were manipulated gently and as little as possible; noises, vibrations and odors were minimized to prevent stress and decreased breeding performance; and enrichment was always used per the facility's guidelines to help alleviate stress and improve breeding. These details are also included in the article.

Wild animals

The study did not involve wild animals.

Field-collected samples

The study did not involve samples collected from field.

Ethics oversight

Mice were housed according to institutional guidelines and all experimental procedures were performed in compliance with the institutional guidelines for the welfare of experimental animals approved by the Universidad Autónoma de Madrid Ethics Committee (CEI 60-1057-A068) and La Comunidad de Madrid (PROEX 335/14) and in accordance with the guidelines for Ethical Conduct in the Care and Use of Animals as stated in The International Guiding Principles for Biomedical Research involving Animals, developed by the Council for International Organizations of Medical Sciences (CIOMS).

Note that full information on the approval of the study protocol must also be provided in the manuscript.

Human research participants

Policy information about studies involving human research participants

Population characteristics

For the human serum samples used in this study, no identifying information was obtained or used. The only information provided and taken into consideration was the tumor stage. Patients with Resectable tumors (n = 14), locally-advanced tumors (n = 17) and metastasis (met) (n = 19) patients. Also included was serum from healthy individuals (n = 21) that were neither ago or sex matched.

Recruitment

Patients were not recruited for this specific study, but rather samples were retrospectively obtained from collections already established by the the BioBank Hospital Ramón y Cajal-IRYCIS (PT13/0010/0002, ISCIII Biobank Register No. B.0000678) or by Dr.

Alfredo Carrato from the “Collection of samples of the Familial Pancreas Cancer Registry”. Samples used are non-identifiable, as detailed above.

Ethics oversight	Serum samples were provided by the BioBank Hospital Ramón y Cajal-IRYCIS (PT13/0010/0002), integrated in the Spanish National Biobanks Network (ISCIII Biobank Register No. B.0000678) and by Dr. Alfredo Carrato from the “Collection of samples of the Familial Pancreas Cancer Registry” of the Carlos III Institute (ISCIII ref nº:C.0003953). Samples were processed following standard operating procedures with the appropriate approval of the Ethical and Scientific Committees (Control no. No. Control: DE-BIOB-73 AC65, RG.BIOB-57, and RG.BIOB-54), with informed consent and according to Declaration of Helsinki principles.

Note that full information on the approval of the study protocol must also be provided in the manuscript.

Flow Cytometry

Plots

Confirm that:

☒ The axis labels state the marker and fluorochrome used (e.g. CD4-FITC).

☒ The axis scales are clearly visible. Include numbers along axes only for bottom left plot of group (a 'group' is an analysis of identical markers).

☒ All plots are contour plots with outliers or pseudocolor plots.

☒ A numerical value for number of cells or percentage (with statistics) is provided.

Methodology

Sample preparation	Cells were trypsinzed, blocked with Flebogamma and resuspended in Flow buffer [1X PBS; 3% FBS (v/v); 3mM EDTA (v/v)] with or without the appropriate diluted fluorescently-tagged antibody against the antigen of choice or with an IgG control.
Instrument	Invitrogen™ Attune™ NxT (cytometry), FACS Vantage SE Flow Cytometer (sorting)
Software	Invitrogen™ Attune™ NxT software, version 3.1.1 was used for cytometry data collection, BD FACSDiVa software was used for sorting data collection, and FlowJo 9.3 software (Tree Star Inc., Ashland, OR) was used for flow cytometry image preparation and analysis.
Cell population abundance	Cancer stem cells represent a small percentage of the total cell population (<10%) and often times purity is not confirmed due to low cell numbers recovered post sorting. When confirmed using the Invitrogen™ Attune™ NxT, purity is typically between 80-90%.
Gating strategy	Generally, cells was first gated on FSC-Area/FSC-Height to remove aggregates. Dead cells were removed by gating in DAPI-negative cells versus FSC-Area. Debris free, live, single cells were gated using FSC-Area and SSC-Area. Surface antigen gating was performed on the live, single, debris free cell population. Gates were determined based on negative controls, unstained controls or IgG controls. A figure exemplifying all of the gating strategies used and controls is provided in Supplementary Figure 17.

☒ Tick this box to confirm that a figure exemplifying the gating strategy is provided in the Supplementary Information.
